# Functional variants in a TTTG microsatellite on 15q26.1 cause familial nonautoimmune thyroid abnormalities

**DOI:** 10.1038/s41588-024-01735-5

**Published:** 2024-05-07

**Authors:** Satoshi Narumi, Keisuke Nagasaki, Mitsuo Kiriya, Erika Uehara, Kazuhisa Akiba, Kanako Tanase-Nakao, Kazuhiro Shimura, Kiyomi Abe, Chiho Sugisawa, Tomohiro Ishii, Kenichi Miyako, Yukihiro Hasegawa, Yoshihiro Maruo, Koji Muroya, Natsuko Watanabe, Eijun Nishihara, Yuka Ito, Takahiko Kogai, Kaori Kameyama, Kazuhiko Nakabayashi, Kenichiro Hata, Maki Fukami, Hirohito Shima, Atsuo Kikuchi, Jun Takayama, Gen Tamiya, Tomonobu Hasegawa

**Affiliations:** 1 Department of Pediatrics, Keio University School of Medicine, Tokyo, Japan; 2 Department of Molecular Endocrinology, National Research Institute for Child Health and Development, Tokyo, Japan; 3 Division of Pediatrics, Department of Homeostatic Regulation and Development, Niigata University Graduate School of Medical and Dental Sciences, Niigata, Japan; 4 Department of Clinical Laboratory Science, Faculty of Medical Technology, Teikyo University, Tokyo, Japan; 5 Division of Endocrinology and Metabolism, Tokyo Metropolitan Children’s Medical Center, Tokyo, Japan; 6 Department of Internal Medicine, Ito Hospital, Tokyo, Japan; 7 Department of Endocrinology and Metabolism, Fukuoka Children’s Hospital, Fukuoka, Japan; 8 Department of Pediatrics, Shiga University of Medical Science, Otsu, Japan; 9 Department of Endocrinology and Metabolism, Kanagawa Children’s Medical Center, Yokohama, Japan; 10 Center for Excellence in Thyroid Care, Kuma Hospital, Kobe, Japan; 11 Department of Genetic Diagnosis and Laboratory Medicine, Dokkyo Medical University, Mibu, Japan; 12 Department of Pathology, Showa University Northern Yokohama Hospital, Yokohama, Japan; 13 Department of Maternal-Fetal Biology, National Research Institute for Child Health and Development, Tokyo, Japan; 14 Department of Human Molecular Genetics, Gunma University Graduate School of Medicine, Maebashi, Japan; 15 Department of Pediatrics, Tohoku University Graduate School of Medicine, Sendai, Japan; 16 Department of AI and Innovative Medicine, Tohoku University Graduate School of Medicine, Sendai, Japan; 17 Department of Integrative Genomics, Tohoku Medical Megabank Organization (ToMMo) Tohoku University, Sendai, Japan; 18 Statistical Genetics Team, RIKEN Center for Advanced Intelligence Project, Tokyo, Japan

**Keywords:** Thyroid diseases, Genetics research

## Abstract

Insufficient thyroid hormone production in newborns is referred to as congenital hypothyroidism. Multinodular goiter (MNG), characterized by an enlarged thyroid gland with multiple nodules, is usually seen in adults and is recognized as a separate disorder from congenital hypothyroidism. Here we performed a linkage analysis of a family with both nongoitrous congenital hypothyroidism and MNG and identified a signal at 15q26.1. Follow-up analyses with whole-genome sequencing and genetic screening in congenital hypothyroidism and MNG cohorts showed that changes in a noncoding TTTG microsatellite on 15q26.1 were frequently observed in congenital hypothyroidism (137 in 989) and MNG (3 in 33) compared with controls (3 in 38,722). Characterization of the noncoding variants with epigenomic data and in vitro experiments suggested that the microsatellite is located in a thyroid-specific transcriptional repressor, and its activity is disrupted by the variants. Collectively, we presented genetic evidence linking nongoitrous congenital hypothyroidism and MNG, providing unique insights into thyroid abnormalities.

## Abstract (web summary)

Noncoding variants in a TTTG microsatellite on 15q26.1 are identified in Japanese patients with childhood and adult-onset thyroid abnormalities. Functional analyses suggest that these variants affect the role of the microsatellite as a potential regulator of thyroid cell growth.

## Main

Thyroid hormones, triiodothyronine and thyroxine (T_4_), regulate the metabolic activity of virtually all human tissues and cause, when depleted in children, growth restriction, intellectual disability and various symptoms. The thyroid gland, an organ producing thyroid hormones, shows great size variation in humans from aplastic (<1 ml), normal (10–20 ml in adults) to enlarged (goiter; maximum >1,000 ml).

Congenital hypothyroidism occurs in about 1 in 2,000‒3,000 births worldwide^1^ and is recognized as one of the major preventable causes of intellectual disability. In developed countries, universal newborn screening for congenital hypothyroidism was started in 1970s^2^, and most patients with congenital hypothyroidism have received early diagnosis and treatment. Congenital hypothyroidism cases can be divided into the following two major categories: goitrous congenital hypothyroidism with an enlarged thyroid gland and nongoitrous congenital hypothyroidism with a small- or normal-sized thyroid. From a clinical genetic standpoint, distinction between the two is important because goitrous congenital hypothyroidism is mostly due to autosomal recessive Mendelian diseases such as the thyroglobulin defect^3^, the thyroid peroxidase defect^4^ and dual oxidase 2 defect^5^, whereas only small fraction (<10%) of nongoitrous congenital hypothyroidism is explained by Mendelian diseases, including the thyroid-stimulating hormone (TSH) receptor defect and the PAX8 defect^6,7^. Five families presenting autosomal dominant nongoitrous congenital hypothyroidism with a linkage region on the long arm of chromosome (chr) 15 were reported^8^, but the disease-causing variant(s) have not been clarified.

Multinodular goiter (MNG) is a disease characterized by the development of multiple thyroid nodules, which can be fluid-filled (cystic) or solid. Although MNG is usually benign, symptoms associated with local compression may occur if left untreated. The exact cause of MNG is not well understood, although several risk factors such as age, female sex and iodine deficiency are known^9^.

In this study, we performed a linkage analysis of a Japanese family in which both nongoitrous congenital hypothyroidism and MNG were observed. Follow-up analyses with whole-genome sequencing (WGS) of additional families and large-scale genetic screening led us to identify the nucleotide-level changes affecting a TTTG microsatellite that are responsible for the peculiar age-dependent thyroid abnormalities. Marked expansions of microsatellite repeats have been known to cause neurodegenerative diseases such as Huntington’s disease^10^, but subtle changes in microsatellites very rarely cause human diseases, including thyroid diseases.

## Results

### Linkage analysis

**Fig. 1:**
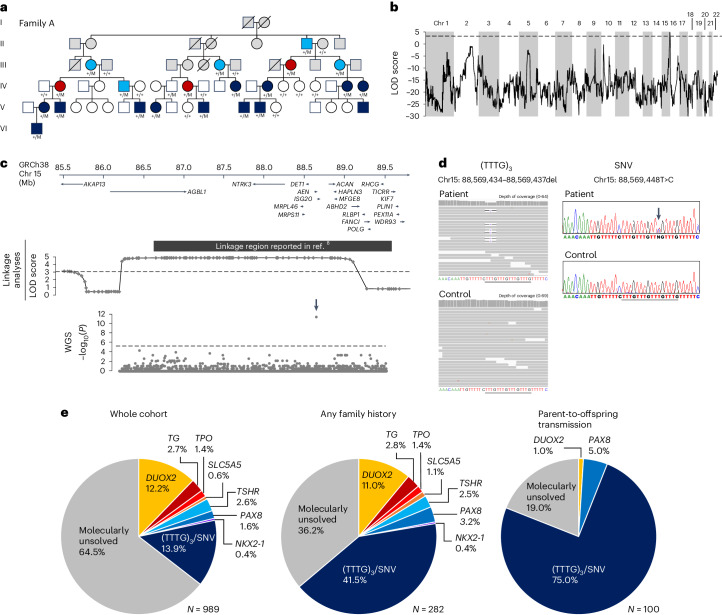
Genetic analyses. **a**, Pedigree of family A. Dark blue, light blue, red, white and gray symbols indicate individuals with childhood-onset compensated hypothyroidism, subclinical hypothyroidism diagnosed in adulthood, suspected MNG, normal thyroid function and unknown thyroid function, respectively. M indicates (TTTG)_3_. **b**, Results of linkage analysis for family A: multipoint logarithm of odds (LOD) score plot for 22 autosomes. The broken line indicates the significance threshold. **c**, Gene locus map of 15q26.1 (GRCh38 chr15: 85.5–89.7 Mb), aligned with the multipoint LOD score graph, and plots showing the difference in the density of rare variants between patients and controls with unsolved familial congenital hypothyroidism. The difference of each 500-bp region (*n* = 6,413) is expressed as −log_10_(*P*) based on calculations by one-sided Fisher’s exact test. The arrow indicates the significant region. Broken lines indicate the significance threshold. **d**, Integrative Genomics Viewer images and partial electropherograms showing the (TTTG)_3_ and SNV (indicated by an arrow). The position of (TTTG)_4_ is underlined. **e**, Frequencies of (TTTG)_3_, SNV and other genetic defects among the congenital hypothyroidism patient cohort. The data are shown as a whole (left), a subgroup with family history (middle) and a subgroup with parent-to-offspring transmission of congenital hypothyroidism (right). Source data

**Extended Data Fig. 1:**
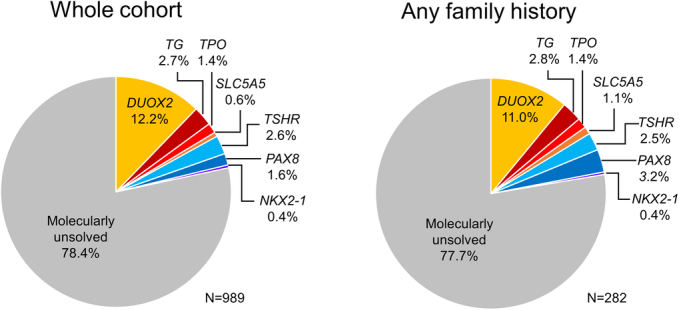
Molecular diagnosis of Japanese congenital hypothyroidism. Results of targeted next-generation sequencing of known causative genes of congenital hypothyroidism are shown. Even when restricted to patients with family history, about three-quarters of patients were not molecularly resolved. Source data

In the last 16 years, we have conducted targeted sequencing in 989 patients with congenital hypothyroidism and determined the molecular diagnoses in 214 cases. Of the 282 cases with family history, only 63 were molecularly solved (Extended Data Fig. 1). Among the unsolved familial cases, there was a large family (family A) with 13 individuals with nongoitrous congenital hypothyroidism, five with subclinical hypothyroidism and three with suspected MNG based on high serum thyroglobulin levels (Fig. 1a). Linkage analysis identified a 3.2-Mb critical region on chr 15q26.1 (GRCh38 chr15: 86,206,051‒89,412,131, maximum logarithm of odds score = 4.8; Fig. 1b). The linkage region overlapped with a previously reported region linked to nongoitrous congenital hypothyroidism (Fig. 1c)^8^. The region encompasses 11 protein-coding genes, but no candidate variant was found by exome sequencing (Supplementary Table 1).

### WGS of unsolved congenital hypothyroidism families

**Extended Data Fig. 2:**
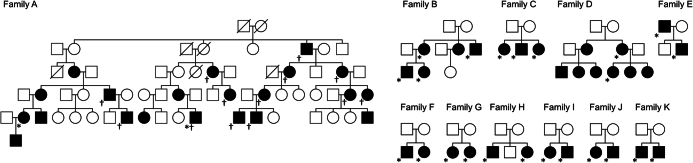
Patients analyzed by linkage analysis and whole-genome sequencing (WGS). Pedigrees of 11 unsolved congenital hypothyroidism families subject to whole-genome sequencing are shown. Black symbols indicate patients with congenital hypothyroidism, subclinical hypothyroidism or multinodular goiter (MNG). Daggers and asterisks denote individuals subject to linkage analysis and WGS, respectively.

**Extended Data Fig. 3:**
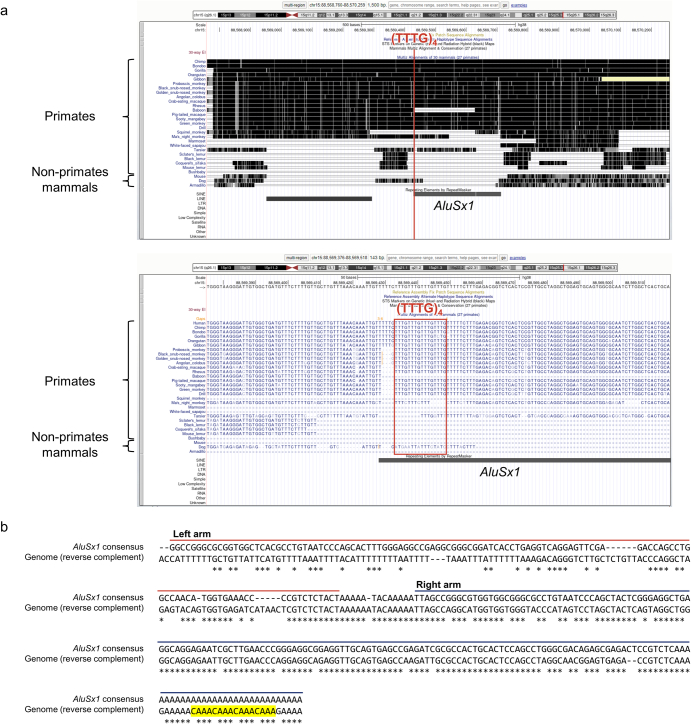
Genomic location of the disease-associated microsatellite. **a**, UCSC genome browser images showing the location of disease-associated microsatellite (TTTG)_4_, sequence conservation in 27 primates and three non-primate mammals and repetitive sequences. Note that the microsatellite is part of an Alu element conserved in a subset of primates. **b**, Comparison of the *AluSx1* consensus sequence with the genomic sequence containing disease-associated microsatellite; the location of (TTTG)_4_, which corresponds to the polyA tail of *AluSx1*, is highlighted in yellow.

We hypothesized that family A and a subset of the molecularly unsolved congenital hypothyroidism families would have common or similar genetic change(s) in noncoding sequences within the linkage region. We selected ten unsolved congenital hypothyroidism families (23 patients; Extended Data Fig. 2) for the next genetic investigation, which are as follows: three families with three or more affected members, one parent-offspring pair and six sibling pairs. These ten families and family A were subject to WGS. As a control, 56 healthy Japanese individuals were sequenced. In the 3.2 Mb linkage region, we identified 2,446 rare variants, defined by allele frequency <0.002 in Tohoku Medical Megabank Organization (ToMMo)^11^, in the 81 participants. The linkage region was divided into 6,413 regions of 500 bp in size, and for each region, the density of rare variants was compared between the congenital hypothyroidism and control groups. We identified a single 500-bp region with a high density of rare variants in the congenital hypothyroidism group (Fig. 1c). In the region, an identical heterozygous 4-bp deletion (GRCh38 chr15: 88,569,434–88,569,437del) was found in 8 of the 11 unsolved families. The variant affects a (TTTG)_4_ microsatellite, reducing the number of repeats from four to three times (designated as (TTTG)_3_; Fig. 1d). This microsatellite is located on the polyA tail of an *Alu* element, which is conserved in some primates but is not observed in nonprimate mammals (Extended Data Fig. 3). (TTTG)_3_ is an ultrarare variant observed in 3 of 38,722 healthy Japanese individuals registered in 38KJPN. The variant has been observed in non-Japanese populations, but at low frequencies in all of them (Supplementary Table 2). An increase in the number of TTTG repeat, namely (TTTG)_5_, is also registered in TOPMed Freeze 10 (ref. ^12^) and ChinaMAP^13^ (Supplementary Table 2).

### Screening in the congenital hypothyroidism cohort

To identify congenital hypothyroidism patients with (TTTG)_3_ in the entire patient cohort, we conducted a PCR-based screen in 964 patients who were not subject to WGS. Further, 121 patients (73 families) with (TTTG)_3_ and two patients with a previously unreported single-nucleotide variant (SNV; GRCh38 chr15: 88,569,448T>C, designated as SNV) affecting the TTTG microsatellite were identified (Fig. 1d and Supplementary Fig. 1). In the whole congenital hypothyroidism patient cohort (*n* = 989), (TTTG)_3_ and SNV accounted for 13.9% of cases (Fig. 1e), which was significantly higher than the general population (*P* < 10^−300^). In the subgroup of patients with family history, the proportion of variant carriers increased to 41.5%, and it reached 75.0% when limited to patients with parent-to-offspring transmission (Fig. 1e).

Of the 83 variant-carrying families, parental genotyping was performed in 51. In 50 of 51 analyzed families, transmission of the variant from either the father or the mother was confirmed. De novo acquisition of (TTTG)_3_ was observed in one family (family 67; Supplementary Fig. 1). To test whether inherited (TTTG)_3_ was derived from a common ancestor, we performed haplotype analysis. We found four distinct haplotypes segregated with (TTTG)_3_ (Supplementary Fig. 2), indicating that (TTTG)_3_ has been acquired on at least four chromosomes independently. Notably, it shows that (TTTG)_3_ is not one of the surrogate variants, but that (TTTG)_3_ itself is the disease-causing variant.

### Childhood phenotypes

**Fig. 2:**
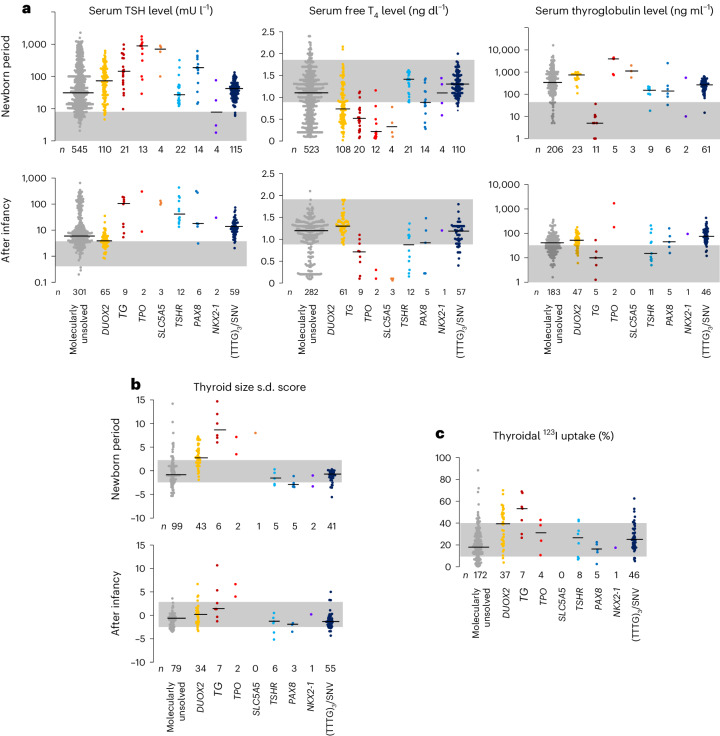
Childhood phenotypes. **a**, Results of thyroid function test (TSH, free T_4_ and thyroglobulin) for each molecular defect are shown as one-dimensional scatter plots. **b**, Ultrasonography-based thyroid size data for each molecular defect are shown. **c**, Levels of thyroidal radioiodine uptake for each molecular defect are shown. For **a‒c**, lines and shaded areas indicate medians and reference intervals, respectively. For thyroid function tests and thyroid size, plots were shown separately for newborn and postinfantile periods. Measurements were taken from distinct samples. Source data

**Extended Data Fig. 4:**
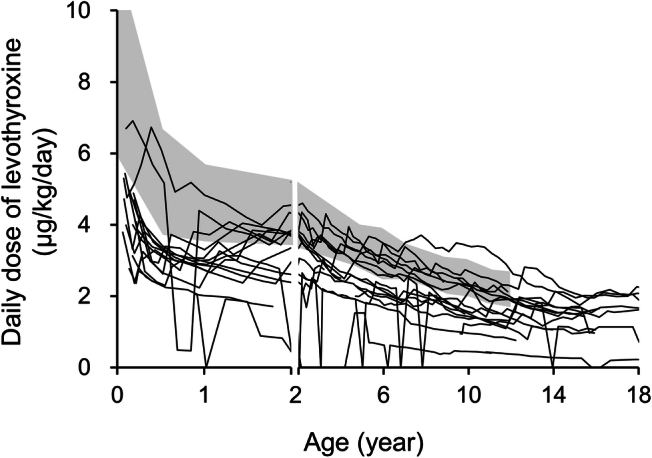
Chronological changes of daily levothyroxine doses in compensated hypothyroidism patients with (TTTG)_3_. The shaded area indicates the range (−1 to +1 standard deviation) of ‘complete’ replacement obtained from congenital hypothyroidism patients with thyroid aplasia (ref. ^11^). Source data

**Extended Data Fig. 5:**
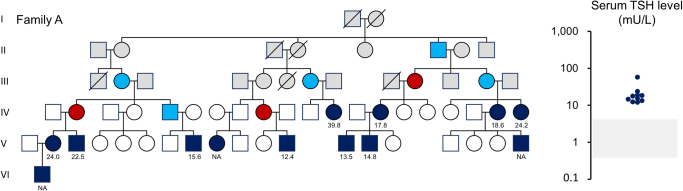
Intrafamilial variability of clinical phenotypes of congenital hypothyroidism patients with (TTTG)_3_. Left panel shows the pedigree of family A. Dark blue, light blue, red, white and gray symbols indicate individuals with childhood-onset compensated hypothyroidism, subclinical hypothyroidism diagnosed in adulthood, suspected multinodular goiter (MNG), normal thyroid function and unknown thyroid function, respectively. The numbers shown below the symbols indicate serum thyroid-stimulating hormone (TSH) levels evaluated after infancy with transient discontinuation of levothyroxine treatment. NA denotes not available. Right panel shows the relatively uniform distribution of the serum TSH levels of the congenital hypothyroidism patients in family A.

Clinical phenotypes of the variant-carrying patients were relatively uniform. Serum TSH levels were moderately elevated, but free T_4_ levels were usually within the reference interval (that is, compensated hypothyroidism; Fig. 2a). Elevated serum thyroglobulin levels were seen in almost all patients, but it was relatively mild compared to the thyroid peroxidase defect and the dual oxidase 2 defect. In most cases, the size of the thyroid was slightly small as compared with the age-matched mean (Fig. 2b). Levels of thyroidal iodine uptake were variable, including approximately 15% having higher levels than the upper limit of the reference interval (Fig. 2c). The patients usually require levothyroxine replacement permanently with doses of 1.5‒2.5 μg kg^−1^ d^−1^ in adulthood (Extended Data Fig. 4)^14^. No common extrathyroidal complications were observed in the 137 variant-carrying patients. Uncommon complications included intellectual disability with epilepsy (*n* = 2) or without epilepsy (*n* = 2), renal hypoplasia (*n* = 1), congenital hydronephrosis (*n* = 1), Gitelman syndrome (*n* = 1) and ventricular septal defect (*n* = 1). The clinical phenotypes of the variant carriers were relatively uniform within the families, as exemplified by the serum TSH levels of childhood-onset patients in family A (Extended Data Fig. 5).

Two variant-carrying childhood-onset patients were untreated or discontinued treatment. The untreated patient was diagnosed in the newborn period but was followed without treatment because free T_4_ levels were normal despite high serum TSH levels (14.0‒58.2 mU l^−1^; reference 0.5‒5.0). In this patient, palpation revealed goiter at age 12 years and then it progressed. Levothyroxine replacement therapy was initiated at the age of 16 years, and thyroid enlargement was controlled. The other treatment-discontinued case was found to have a high serum TSH level (TSH: 19.9 mU l^−1^) in the newborn period and was treated with levothyroxine until 3 years of age when the treatment was discontinued. Serum TSH levels during the untreated period ranged from 10.7 to 26.1 mU l^−1^. At age 5 years, goiter was noted on palpation. Ultrasonographic findings were consistent with MNG, with the largest nodule measuring 18 mm in diameter. Subsequently, the goiter worsened and the nodules increased in size. Levothyroxine replacement therapy was resumed at the age of 9 years, and then thyroid enlargement and nodule growth were controlled.

Of 137 variant-carrying patients, 6 had an additional heterozygous variant in autosomal recessive congenital hypothyroidism-associated genes, including *DUOX2*, *SLC26A4* and *TSHR* (Supplementary Table 3). Although there was no significant difference in serum TSH levels between the 6 patients and the remaining 131 patients, the highest thyroglobulin level (640 ng ml^−1^) in infancy was observed in the patient who was heterozygous for (TTTG)_3_ and *DUOX2* p.Leu1160del^15^.

### Adult phenotypes

**Fig. 3:**
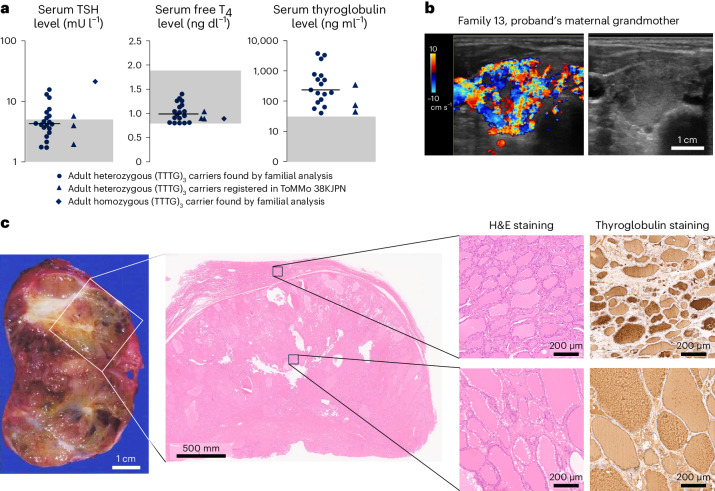
Adult phenotypes. **a**, Results of thyroid function test (TSH, free T_4_ and thyroglobulin) for adult (TTTG)_3_ carriers born before 1979 (circles) and (TTTG)_3_ carriers registered in ToMMo 38KJPN (triangles) are shown as one-dimensional scatter plots. Lines and shaded areas indicate medians and reference intervals, respectively. Measurements were taken from distinct samples. **b**, Representative ultrasonographic image of an untreated adult MNG patient with (TTTG)_3_. This 67-year-old female patient showed remarkable hypervascularization despite a normal serum TSH level (2.31 mU l^−1^). **c**, Resected MNG specimen of a 30-year-old male patient with (TTTG)_3_. Histological analysis of the adenoma showed heterogeneity of the size of follicles and increased resorptive vacuoles (right lower). The magnified images are representative of four independent observations with similar histological findings. H&E staining and thyroglobulin staining. H&E, hematoxylin and eosin. Source data

Through family analysis, additional 76 variant carriers were found, including 58 individuals born before 1979 when newborn screening for congenital hypothyroidism was implemented in Japan. Homozygous (TTTG)_3_ was found in one female patient who was diagnosed with hypothyroidism (TSH: 21.2 mU l^−1^, free T_4_: 0.89 ng dl^−1^) with MNG at the age of 58 years (family 32, the proband’s maternal grandmother; Fig. 3a and Supplementary Figs. 1 and 3). She underwent fine needle aspiration cytology on the largest nodule (size, 13 × 12 × 16 mm), and the result was benign. Except for the thyroid abnormalities, she had no relevant medical history.

Of the 58 adult variant carriers born before 1979, 16 were receiving levothyroxine treatment. In 20 of the 42 untreated individuals, thyroid function test showed high serum TSH in 35%, low serum free T_4_ in 0% and high serum thyroglobulin in 94% (Fig. 3a). We also analyzed serum samples, stored in the biobank, derived from three (TTTG)_3_ carriers registered in ToMMo JPN38K, and found similar biochemical characteristics in them (Fig. 3a). Fourteen of 15 untreated adults who underwent thyroid ultrasonography had MNG (Supplementary Fig. 3). A characteristic increase in blood flow was observed in most cases where blood flow was evaluated (Fig. 3b). Due to the large thyroid nodules, three patients received thyroidectomy, and we could obtain one specimen (Supplementary Fig. 1; family 47, the proband’s maternal uncle; Fig. 3c (left)). Histological analysis revealed a nodule clearly demarcated from the adjacent thyroid tissue by a well-formed sclerotic capsule (Fig. 3c (middle)). The follicles in the nodule were heterogeneous in size, and the number of resorptive vacuoles was increased (Fig. 3c (right). No evidence of malignancy was seen.

We tested whether (TTTG)_3_ and SNV were observed in an independent MNG cohort without a history of childhood-onset hypothyroidism. By sequencing 33 patients with MNG followed at Kuma Hospital, Kobe, we identified two patients with (TTTG)_3_ and one with SNV (Supplementary Fig. 1). This frequency (3 in 33) was significantly higher than the general Japanese population (*P* < 1.2 × 10^−8^).

### Functional analyses

**Fig. 4:**
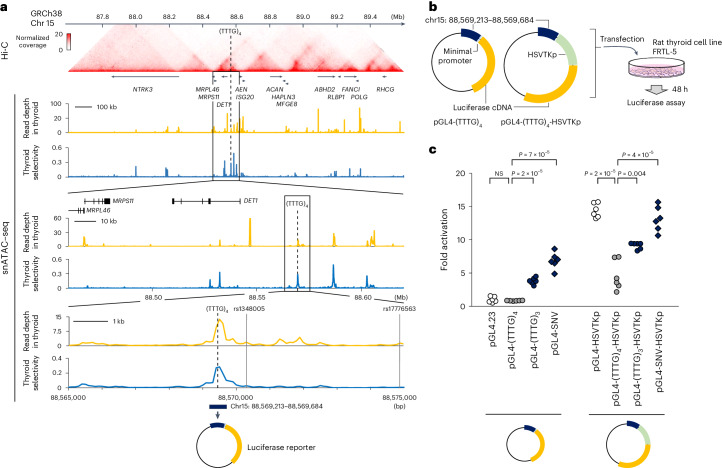
Functional analyses. **a**, Epigenomic analysis of the region surrounding the noncoding microsatellite (TTTG)_4_. Hi-C data analysis showed that three genes (*MRPL46*, *MRPS11* and *DET1*) are located in the chromatin domain to which (TTTG)_4_ belongs. Analysis of snATAC–seq count data revealed that (TTTG)_4_ is located in a region of open chromatin in thyroid follicular cells (yellow lines). Comparison of the count data among 154 cell types revealed that this open chromatin configuration is selective to thyroid follicular cells (blue lines). Two top-hit SNPs of GWAS for thyroid function (rs1348005 and rs17776563) are shown^29,30^. The 472-bp sequence corresponding to the snATAC–seq peak was used to create a luciferase reporter. **b**, Schematic diagram showing cell-based experiments to test the ability of 472-bp sequence to activate or repress gene expression. HSVTKp was used to recapitulate the activation of gene expression. **c**, Results of luciferase assays. The 472-bp sequence exhibited an ability to repress HSVTKp-mediated transcription, while this repression was partially lost by the introduction of (TTTG)_3_ or SNV. Two-sided Welch’s *t* test was used for statistical comparisons. Data distribution was assumed to be normal, but this was not formally tested. NS, not significant. Source data

**Extended Data Fig. 6:**
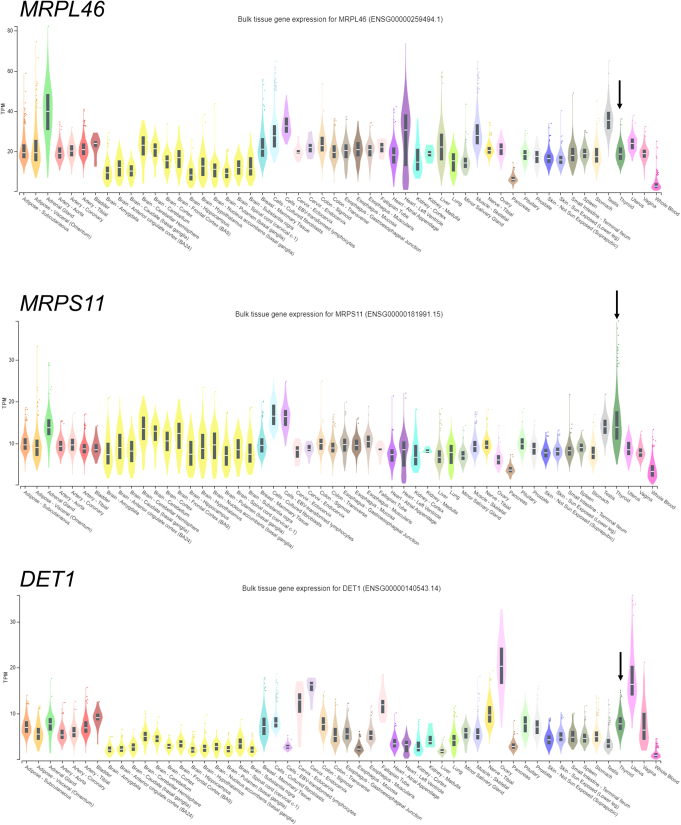
RNA expression levels of *MRPL46*, *MRPS11* and *DET1* by organs. RNA expression values are shown in TPM (transcripts per million). Box plots are shown as median and 25th and 75th percentiles; points are displayed as outliers if they are above or below 1.5 times the interquartile range. Data were obtained from GTEx (https://gtexportal.org/). Each RNA expression data was obtained from 4 to 803 donors (median 246). Arrows indicate thyroid. The three genes show ubiquitous gene expression.

**Extended Data Fig. 7:**
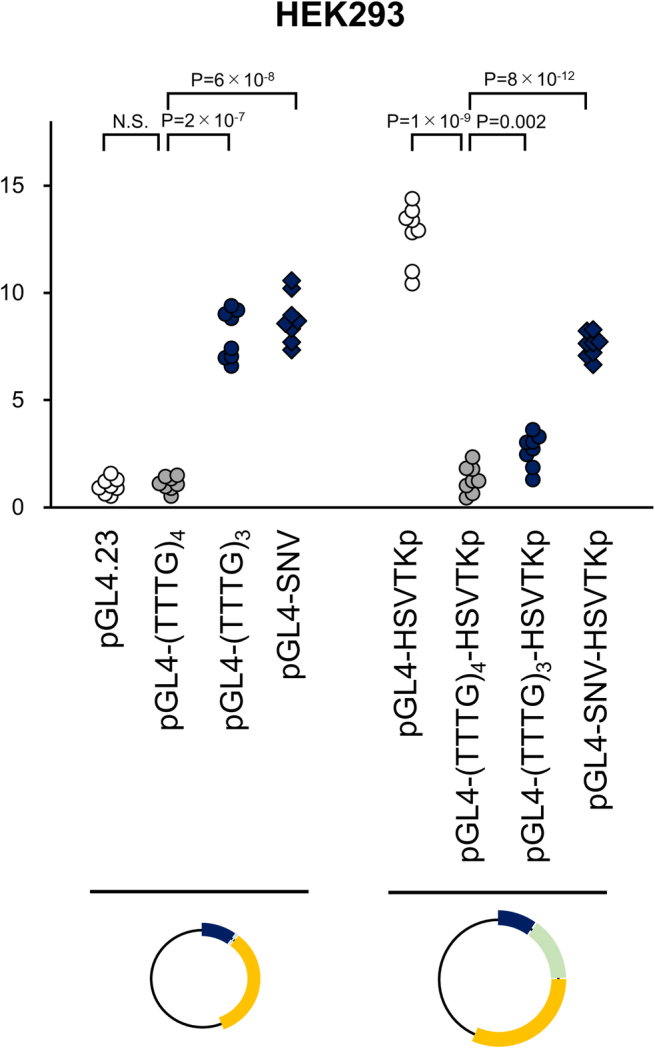
Results of luciferase assays using HEK293 cells. The 472 bp sequence exhibited an ability to repress herpes simplex virus thymidine kinase promoter (HSVTKp)-mediated transcription, while this repression was partially lost by introduction of (TTTG)_3_ or the single nucleotide variant (SNV). Two-sided Welch’s t-test was used for statistical comparisons. Source data

There are >600,000 microsatellites in the human genome^16,17^. Variation in the number of microsatellite repeats is often observed, and most of them are considered to have negligible biological effects. We first questioned whether the region containing the disease-associated microsatellite (designated as (TTTG)_4_) has epigenomic signatures as gene regulatory regions. Analysis of single-nucleus assay for transposase-accessible chromatin (snATAC)–seq count data showed that the (TTTG)_4_-containing region is in an open chromatin configuration in the thyroid (Fig. 4a (yellow lines)). Comparison of snATAC–seq count data derived from 154 cell types indicated that this configuration is highly selective for the thyroid (Fig. 4a (blue lines)). High-throughput chromosome conformation capture (Hi-C) experiment performed in H1-hESC differentiated to definitive endoderm^18^ showed that the (TTTG)_4_ shares the same chromatin domain with three protein-coding genes (*MRPL46*, *MRPS11* and *DET1*; Fig. 4a), all of which are expressed ubiquitously (Extended Data Fig. 6). Altogether these experiments suggest that (TTTG)_4_-containing region could have the capacity to modulate gene expression, functioning as a regulatory element. To this end, we performed a luciferase activity experiment including pGL4-(TTTG)_4_ reporters with or without the herpes simplex virus thymidine kinase promoter (HSVTKp) and measured the luciferase activity in transiently transfected cells (rat thyroid cell line FRTL-5; Fig. 4b). Without the viral promoter, the pGL4-(TTTG)_4_ reporter showed activity comparable to the empty vector. However, the reporter with the viral promoter (pGL4-(TTTG)_4_-HSVTKp) showed reduced HSVTKp-mediated expression of luciferase by 32 ± 6%, suggesting a repressor activity (Fig. 4c). The variant reporters without HSVTKp (pGL4-(TTTG)_3_ and pGL4-SNV) showed substantial basal activities. However, these activities were not additive for HSVTKp; rather, (TTTG)_3_ repressed the activity of HSVTKp. The degree of repression was attenuated as compared to that of the repression by (TTTG)_4_ (Fig. 4c). Similar results were confirmed in experiments using human embryonic kidney 293 cells (Extended Data Fig. 7). Taken together, these bioinformatic and experimental data suggest that the (TTTG)_4_-containing region is a thyroid-specific repressor, and sequence changes affecting the TTTG microsatellite cause loss of the repressor activity.

## Discussion

Here we showed that about three-quarters of cases of dominantly inherited Japanese congenital hypothyroidism are caused by nucleotide changes affecting a noncoding microsatellite in 15q26.1. The untreated or treatment-discontinued cases showed a characteristic transition of the clinical manifestation from nongoitrous congenital hypothyroidism to MNG, linking the two thyroid abnormalities that had been thought to be distinct. This genetic disease has been registered in the Online Mendelian Inheritance in Man as congenital hypothyroidism, nongoitrous, 3 (CHNG3; %609893), also referred to as resistance to TSH^8^. However, we observed no clinical evidence of defective TSH signaling in variant carriers, but rather overcompensation of the signal was presumed—goiter, increased blood flow and increased thyroidal iodine uptake. In the future, more appropriate disease names would be assigned based on precise molecular pathogenesis.

CHNG3 accounted for 13.9% (95% confidence interval, 11.9‒16.1) of our Japanese congenital hypothyroidism cohort and is one of the leading genetic causes of congenital hypothyroidism in Japan (Fig. 1e). This study further showed that approximately 9% of Japanese MNG were attributed to CHNG3, but given the small size of the patient cohort (*n* = 33), the frequency should be validated in a larger cohort. Universal genetic screening for CHNG3 in patients with MNG may be considered if significant clinical characteristics, such as risk of malignant transformation, are identified. Based on the ToMMo 38KJPN data and analysis of stored samples, we estimated the prevalence of CHNG3 in Japan as 1/12,900 (95% confidence interval, 1/5,400 to 1/35,500).

**Extended Data Fig. 8:**
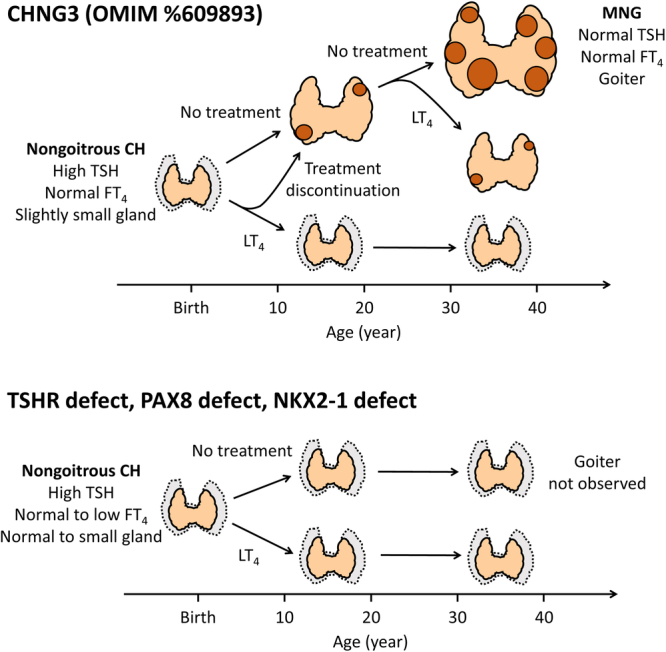
Schematic diagram showing age-dependent changes in the clinical phenotype of CHNG3 (OMIM %609893). Patients with CHNG3 diagnosed through newborn screening, have mild thyroid hypoplasia and do not develop thyroid nodules if treated with levothyroxine (LT_4_). If thyroid abnormalities are not diagnosed in infancy, thyroid hormone production is compensated via thyroid cell proliferation (that is, goiter), eventually leading to multinodular goiter (MNG). LT_4_ treatment can prevent progression of goiter and further formation of nodules. MNG is also observed in childhood-onset patients who stop treatment. This age-dependent transition of phenotypes has not been documented in other Mendelian forms of nongoitrous congenital hypothyroidism (CH), including TSHR defect, PAX8 defect and NKX2-1 defect, and thus is considered a clinical characteristic of CHNG3.

The absence of sequelae of congenital hypothyroidism such as intellectual disability and short stature in untreated adult patients with CHNG3 is likely due to thyroid hormone production via increase of thyroid tissues. In other Mendelian forms of nongoitrous congenital hypothyroidism, including *TSHR* defect^19^, *PAX8* defect^20^ and *NKX2-1* defect^21^, goiter is not usually observed in untreated patients with high serum TSH levels (Extended Data Fig. 8). Therefore, factor(s) other than compensatory TSH stimulation may be involved in the development of goiter and MNG in untreated patients with CHNG3. Although there are no obvious sequelae of congenital hypothyroidism in untreated adult patients with CHNG3, levothyroxine therapy from infancy may have a role in preventing the transition from nongoitrous congenital hypothyroidism to MNG and the resultant need for a fine needle aspiration biopsy and surgery.

The adult untreated individual with homozygous (TTTG)_3_ had a higher serum TSH level than heterozygous variant carriers and had a normal-sized thyroid gland despite the presence of multiple nodules. These observations suggest that the two (TTTG)_3_ alleles cause a more severe reduction in the proliferation capacity of thyroid follicular cells than one allele but do not have catastrophic effects on thyroid development and physiology.

In terms of methods to identify Mendelian disease-causing nucleotide changes in noncoding regions, one or more genetic mapping methods such as analysis of structural variants^22–25^ and linkage analysis (this study and refs. ^24,25^) were used. Because exome sequencing has been popularized, most genetic studies no longer require these mapping methods to identify disease-causing variants in exons. However, given the vastness of the noncoding regions (50 times larger than the coding regions), genetic mapping methods still have an indispensable role in identifying disease-causing noncoding variants.

The disease-causing noncoding variants identified in this study were located inside an *Alu* element. Recent studies have shown that *Alu* elements are involved in epigenomic modifications^26^ and 3D genome conformations^27^ in vitro. Genetic variants of *Alu* associated with altered gene expression levels have been reported^28^, and there is growing interest in the impact of these primate-specific repetitive sequences on the human genome. In our in vitro experiments, the variant luciferase reporters (pGL4-(TTTG)_3_ and pGL4-SNV) had enhancer-like activities, while nonmutated reporter with the viral promoter (pGL4-(TTTG)_4_-HSVTKp) showed a repressor-like activity. In ref. ^28^, a comprehensive analysis of the effects of mobile elements, including *Alu*, on gene expression regulation was performed to observe both positive and negative effects, with negative effects being more common. We assume that the *Alu* element with (TTTG)_4_ was originally acquired as a repressor during primate evolution to optimize thyroid gland anatomy and physiology, but its repressor activity was altered by the shortening of the TTTG repeat.

Two large meta-genome-wide association studies (GWAS) have reported single-nucleotide polymorphisms (SNPs) near (TTTG)_4_ associated with thyroid function—rs17776563 (distance, 6.5 kb)^29^ and rs1348005 (distance, 0.8 kb)^30^ (Fig. 4a). According to the Genotype-Tissue Expression (GTEx) project V8 dataset, these two SNPs are expression quantitative trait loci that affect thyroidal expression of *DET1* (Supplementary Fig. 4), encoding a protein involved in proteasomal degradation of c-Jun^31^. Carriers of the low thyroid function-associated genotypes (rs17776563 A and rs1348005 G) showed high thyroidal *DET1* expression (Supplementary Fig. 4), suggesting that increased expression of *DET1* and resultant decrease of c-Jun would negatively affect the hormone-producing capacity of the thyroid. This assumption agrees well with the known role of the IGF1-Ras-MAPK/c-Jun signaling pathway in thyroid cell growth^32,33^. At present, we cannot pinpoint which gene(s) are repressed by the (TTTG)_4_-containing region, but *DET1* would be the most likely target. Further studies, such as RNA-seq of patient-derived thyroid tissues, are needed to clarify the molecular mechanism(s) linking the noncoding changes and CHNG3.

In summary, through linkage analysis of a large family and genetic screens in patient cohorts, we have shown that nucleotide changes in a microsatellite located in the noncoding region of chr 15q26.1 are responsible for childhood nongoitrous congenital hypothyroidism and adult MNG, which have been considered distinct disease entities. These findings provide unique insights into the relationship between the anatomy and physiology of the thyroid gland.

## Methods

### Ethical consideration

This study was approved by the Ethics Committees of Keio University School of Medicine (approval: 20140289 and 20170130), the National Center for Child Health and Development (approval: 553) and ToMMo (approval: 2022-4-186). This study was conducted in accordance with the Declaration of Helsinki. All participants or their parents provided written informed consent for the molecular studies.

### Study participants and genetic screening

In this study, congenital hypothyroidism was defined as patients with a positive newborn screening result and at least one high serum TSH level by 2 months of age. Cases due to maternal Graves’ disease, exposure to antithyroid drug or exposure to excessive iodine were excluded. Family history was considered positive if first- to third-degree relatives had hypothyroidism of any type. Patients were clinically characterized at each institution. Goitrous congenital hypothyroidism was defined as cases with in situ thyroid gland of which size s.d. score^34^ was equal to or more than +2.0.

From 2006 to 2022, 989 peripheral blood samples derived from congenital hypothyroidism patients (female, 53%) were collected from 119 institutions across Japan. Eleven congenital hypothyroidism-associated genes (*DUOX2, DUOXA2, FOXE1, IYD, NKX2-1, PAX8, SLC5A5, SLC26A4, TG, TPO* and *TSHR*) were sequenced as previously described^35,36^. Detected variants were evaluated based on frequencies in the patient cohort (*n* = 989) versus the general Japanese population (ToMMo 38KJPN, *n* = 38,722), presumed functional impact and/or the results of cell-based experiments as previously described^6,7,15^. For the genetic diagnoses, the mode of inheritance and results of parental genotyping, if available, were considered.

Patients with MNG (*n* = 33, female = 70%, median age = 48 years and interquartile range = 35–63 years) were clinically characterized and enrolled at Kuma Hospital, Kobe city. The definition of MNG was based on the following ultrasonographic findings: (i) enlarged thyroid gland based on ultrasonographic measurements and (ii) multiple thyroid nodules.

### Thyroid ultrasonography

Thyroid ultrasonography was performed for 16 individuals with (TTTG)_3_ at each institution to evaluate the morphology of the thyroid gland. Color-flow Doppler imaging, a noninvasive technique to visualize blood flow, was conducted in 14 of the 16 individuals.

### Thyroid histology and immunohistochemistry

A thyroid tissue sample was obtained from a (TTTG)_3_-carrying individual undergoing thyroidectomy (Supplementary Fig. 1; family 47, the proband’s maternal uncle). The sample was fixed in 10% formalin and embedded in paraffin. Tissue sections were cut at 5 μm thickness, deparaffinized and stained with hematoxylin and eosin using standard procedures.

For immunohistochemical staining of thyroglobulin, 5-μm sections of the formalin-fixed paraffin-embedded thyroid tissue were deparaffinized, and endogenous peroxidase activity was blocked by incubation in 1% H_2_O_2_ to unmask antigens. Then, the slides were microwaved in 10 mM citrate buffer (pH 6) for 15 min. After blocking nonspecific staining with horse serum, the slides were incubated with rabbit antithyroglobulin polyclonal antibody (Dako) at 1:5,000 dilution. Sections were then incubated with biotin-labeled secondary antibody (dilution, 1:500). Subsequent reactions with the ENVISION system (Dako) and diaminobenzidine (Sigma-Aldrich) were followed by counterstaining with hematoxylin.

### Linkage analysis

SNPs of 13 individuals belonging to family A were genotyped with GeneChip Human Mapping 250 K Nsp Array (Thermo Fisher Scientific). Linkage analysis was performed with Superlink Online SNP version 1.1 (ref. ^37^) to calculate multipoint logarithm of the odds scores. We performed affected-only analysis (see Supplementary Fig. 2 for analyzed participants) with a 100% penetrant autosomal dominant model and an assumption of no phenocopies. Disease allele frequency was set to 0.0001.

### WGS

We performed WGS on 25 patients with unsolved familial congenital hypothyroidism. We also sequenced 56 ancestry-matched controls from the National Center Biobank Network resource^38^ using the identical analytical pipeline. DNA libraries were constructed with TruSeq DNA PCR-Free (Illumina) and sequenced with NovaSeq6000 (Illumina) or DNB-SEQ (MGI). Read mapping to GRCh38 and variant calling were conducted with DRAGEN v3.9.5 (Illumina).

In this study, rare variants were defined as allele frequency <0.002 in 38KJPN. The 3.2-Mb linkage region (GRCh38 chr15: 86,206,001–89,412,500) was divided into 6,413 regions with a size of 500 bp, and rare variants were counted for the patient and control groups. Fisher’s exact test was used to compare the density of rare variants in each region with the Bonferroni-corrected significance threshold at *P* = 7.8 × 10^−6^ (=0.05/6,413).

### PCR-based screening

A 455-bp region (GRCh38 chr15: 88,569,231–88,569,685) was subject to PCR-based Sanger sequencing in patients with congenital hypothyroidism and MNG. Genetic and biochemical analyses for family members of the variant-carrying patients were performed if consent for the study was obtained. The sequences of primers are shown in Supplementary Table 4.

### Haplotype analysis

(TTTG)_3_-carrying patients and their relatives were genotyped for five short-tandem repeat markers (D15S0299i, D15S199, D15S979, D15S0407i and D15S0496i) and four SNPs (rs201709422, rs1348002, rs61650474 and rs191942900) with use of the fragment analysis method and PCR-based next-generation sequencing, respectively (Supplementary Table 5).

### Biobank sample analysis

ToMMo conducted WGS in 38,722 healthy Japanese individuals, and the summary statistics is publicly available as 38KJPN (https://jmorp.megabank.tohoku.ac.jp/). In the biobank cohort, (TTTG)_3_ was observed in three individuals. Using the frozen serum samples derived from the three individuals, we measured levels of TSH (Lumipulse Presto TSH IFCC; Fujirebio), free T_4_ (Lumipulse Presto FT4; Fujirebio), thyroglobulin (Lumipulse Presto iTACT Tg; Fujirebio), antithyroglobulin antibody (Lumipulse Presto TgAb; Fujirebio) and antithyroid peroxidase antibody (Lumipulse Presto TPOAb; Fujirebio).

### Hi-C

We used Hi-C data of H1-hESC differentiated to definitive endoderm (accession: 4DNESCOQ5YRS; 4D Nucleome Data Portal) that was generated in the 4D nucleome project^18^. Using the Hi-C data, 5-kb resolution contact matrices corresponding to chr15: 87,600,000–89,600,000 were visualized using Juicebox Web App (v2.3.5)^39^.

### snATAC–seq

snATAC–seq read count data of 154 human cell types, generated in ref. ^40^, were downloaded from Human Enhancer Atlas (http://catlas.org/humanenhancer/). The obtained data (bigWig format) were analyzed with Megadepth (version 1.2.0)^41^ to calculate regional mean read depth in 100-bp bins for each of the 154 cell types. Thyroid selectivity of snATAC–seq was defined by (median read depth of thyroid follicular cell)/(sum of median read depth of 154 cell types). Read depth in the thyroid and thyroid selectivity were visualized with Microsoft Excel 2019.

### Luciferase reporter assay

A 472-bp genomic region (GRCh38 chr15: 88,569,213–88,569,684) containing the disease-associated microsatellite was PCR amplified. We cloned the PCR product into pGL4.23 (Promega), which contains the minimal promoter and firefly luciferase sequences (pGL4-(TTTG)_4_) using the Gibson assembly technique (NEBuilder HiFi DNA Assembly Master Mix; New England Biolabs). Two disease-associated variants were introduced with a standard site-directed mutagenesis technique (pGL4-(TTTG)_3_ and pGL4-SNV). These reporter vectors were used to evaluate the ability of the 472-bp sequence to enhance gene expression. We also created vectors in which the minimal promoter was replaced by HSVTKp (pGL4-(TTTG)_4_-HSVTKp; Supplementary Methods), which were used to evaluate the ability of the 472-bp sequence to repress gene expression. The complete sequences of the luciferase reporter vectors are shown in Supplementary Methods.

Rat thyroid FRTL-5 cells were cultured in Coon’s modified Ham’s F-12 medium supplemented with 5% bovine serum and a mixture of six hormones (10 μg ml^−1^ insulin, 0.36 ng ml^−1^ hydrocortisone, 5 μg ml^−1^ transferrin, 10 ng ml^−1^ somatostatin, 2 ng ml^−1^ glycyl-l-histidyl-l-lysine acetate and 1 mU ml^−1^ TSH) as previously described^42^. Cells seeded into six-well plates were transfected with 1 μg of each luciferase reporter plasmid using FuGENE HD Transfection Reagent (Promega). Forty-eight hours after the transfection, luciferase assays were performed with cells lysed in Glo Lysis Buffer (Promega) and Bright-Glo Luciferase Assay System (Promega). Protein concentrations of each cell lysate were measured with the DC Protein Assay Kit (Bio-Rad Laboratories), and the luminescence levels were normalized. The luciferase activities are reported relative to the activity of the empty pGL4.23 vector (that is, fold activation). The data are representative of three independent transfection experiments each performed as hexaplicate (FRTL-5) or octuplicate (HEK293). Two-sided Welch’s *t* test was used for statistical comparisons.

### Statistics and reproducibility

This is a cross-sectional observational study. No statistical method was used to predetermine sample size as this study involved a rare congenital disease. Histological studies were performed on a resected thyroid sample from one individual. The study was conducted once due to limited sample availability. For the magnified view, representative images of four independent fields are shown. Luciferase assays were performed three times as independent transfection experiments, and similar results were reproduced. No data were excluded from any of the experiments described. Data collection and analysis were not performed blind to the conditions of the experiments.

### Reporting summary

Further information on research design is available in the Nature Portfolio Reporting Summary linked to this article.

## Online content

Any methods, additional references, Nature Portfolio reporting summaries, source data, extended data, supplementary information, acknowledgements, peer review information; details of author contributions and competing interests; and statements of data and code availability are available at 10.1038/s41588-024-01735-5.

### Supplementary information

Supplementary Information Supplementary Methods and Figs. 1–4.

Reporting Summary

Supplementary Tables Supplementary Tables 1–5.

### Source data

Source Data Fig. 1 Statistical source data.

Source Data Fig. 2 Statistical source data.

Source Data Fig. 3 Statistical source data.

Source Data Fig. 3 Unprocessed images of ultrasonography and histology.

Source Data Fig. 4 Statistical source data.

Source Data Extended Data Fig. 1 Statistical source data.

Source Data Extended Data Fig. 4 Statistical source data.

Source Data Extended Data Fig. 7 Statistical source data.

## Data Availability

Hi-C data of H1-hESC differentiated to definitive endoderm (accession 4DNESCOQ5YRS; 4D Nucleome Data Portal, https://data.4dnucleome.org/experiment-set-replicates/4DNESCOQ5YRS/) were visualized using Juicebox Web App (https://aidenlab.org/juicebox/). snATAC–seq read count data were retrieved from Human Enhancer Atlas (http://catlas.org/humanenhancer/)^40^. Frequency data of genetic variants in 38,722 healthy Japanese individuals (38KJPN) were obtained from jMorp (https://jmorp.megabank.tohoku.ac.jp/). Expression quantitative trait locus data (GTEx v8 dataset) were downloaded from the GTEx portal (https://gtexportal.org/home/datasets). To preserve the confidentiality of the study participants, restrictions apply to the use of the WGS data generated in this study. The corresponding author will, upon request, provide details of the restrictions and the conditions under which access to some of the data may be provided. Source data are provided with this paper.

## Code availability

All software used in the study are publicly available as described in Methods and the Reporting Summary.
