# Supplementary material for: Functional variants in a TTTG microsatellite on 15q26.1 cause familial nonautoimmune thyroid abnormalities
Source: Nat Genet. 2024 May 7;56(5):869–76. doi: 10.1038/s41588-024-01735-5 (PMC11096107; doi:10.1038/s41588-024-01735-5)

nature genetics

Article

https://doi.org/10.1038/s41588-024-01735-5

Functional variants in a TTTG microsatellite on 15q26.1 cause familial nonautoimmune thyroid abnormalities

In the format provided by the authors and unedited

Functional variants in a TTTG microsatellite on 15q26.1 cause familial non-autoimmune thyroid abnormalities

Satoshi Narumi, Keisuke Nagasaki, Mitsuo Kiriya, Erika Uehara, Kazuhisa Akiba, Kanako Tanase-Nakao, Kazuhiro Shimura, Kiyomi Abe, Chiho Sugisawa, Tomohiro Ishii, Kenichi Miyako, Yukihiro Hasegawa, Yoshihiro Maruo, Koji Muroya, Natsuko Watanabe, Eijun Nishihara, Yuka Ito, Takahiko Kogai, Kaori Kameyama, Kazuhiko Nakabayashi, Kenichiro Hata, Maki Fukami, Hirohito Shima, Atsuo Kikuchi, Jun Takayama, Gen Tamiya and Tomonobu Hasegawa

Supplementary Data

Supplementary Methods

Sequences of luciferase reporter vectors…. Page 2

Supplementary Figures

Supplementary Fig. 1…..…………….…... Page 6
Supplementary Fig. 2…..…………….…... Page 11
Supplementary Fig. 3…..…………….…... Page 13
Supplementary Fig. 4…..…………….…... Page 16
Source data…………….….………….…... Page 19

Sequences of luciferase reporter vectors

pGL4-(TTTG)$_4$

GGCCTAACTGGCCGGTACCTGAGCTCGCTAGCCTCGAGGATATCAAGATCTGGCCTCGGCGGCCACAATACAGCCCTGTGACACAAGTTTA

CCTACATAACGAACCTGCACATGTATCCCTGAACCTAAAATAAAAATTGAAAACAATAAATAAATAAATACAATATGTCAACAAGAACATA

TGGGAAGAACCATGCCCAAAATGAAATAGTACAAAATGGTCATGCTTGGGTAAGGGATTGTGGCTGATGTTTCTTTTGTTGCTTGTTTAAA

CAAATTGTTTTTC**TTTGTTTGTTTGTTTG**TTTTTCTTTGAGACGGTCTCACTCCGTTGCCTAGGCTGGAGTGCAGTGGCGCAATCTTGGCT

CACTGCAACCTCTGCCTCCTGGGTTCAAGCAATTCTCCTGCCCCAGCCTACTGAGTAGCTAGGACTATGGGTACCCACCACCATGCCTGGC

TAATTTTTGTATTTTTTAGTAGAGACGAGTTATGATCTCACCACTGTACTCTAGCCTGGGTAACAGAGCAAGACCCTGTCTTTAGACACTA

GAGGGTATATAATGGAAGCTCGACTTCCAGCTTGGCAATCCGGTACTGTTGGTAAAGCCACCATGGAAGATGCCAAAAACATTAAGAAGGG

CCCAGCGCCATTCTACCCACTCGAAGACGGGACCGCCGGCGAGCAGCTGCACAAAGCCATGAAGCGCTACGCCCTGGTGCCCGGCACCATC

GCCTTTACCGACGCACATATCGAGGTGGACATTACCTACGCCGAGTACTTCGAGATGAGCGTTCGGCTGGCAGAAGCTATGAAGCGCTATG

GGCTGAATACAAACCATCGGATCGTGGTGTGCAGCGAGAATAGCTTGCAGTTCTTCATGCCCGTGTTGGGTGCCCTGTTCATCGGTGTGGC

TGTGGCCCCAGCTAACGACATCTACAACGAGCGCGAGCTGCTGAACAGCATGGGCATCAGCCAGCCCACCGTCGTATTCGTGAGCAAGAAA

GGGCTGCAAAAGATCCTCAACGTGCAAAAGAAGCTACCGATCATACAAAAGATCATCATCATGGATAGCAAGACCGACTACCAGGGCTTCC

AAAGCATGTACACCTTCGTGACTTCCCATTTGCCACCCGGCTTCAACGAGTACGACTTCGTGCCCGAGAGCTTCGACCGGGACAAAACCAT

CGCCCTGATCATGAACAGTAGTGGCAGTACCGGATTGCCCAAGGGCGTAGCCCTACCGCACCGCACCGCTTGTGTCCGATTCAGTCATGCC

CGCGACCCCATCTTCGGCAACCAGATCATCCCCGACACCGCTATCCTCAGCGTGGTGCCATTTCACCACGGCTTCGGCATGTTCACCACGC

TGGGCTACTTGATCTGCGGCTTTCGGGTCGTGCTCATGTACCGCTTCGAGGAGGAGCTATTCTTGCGCAGCTTGCAAGACTATAAGATTCA

ATCTGCCCTGCTGGTGCCCACACTATTTAGCTTCTTCGCTAAGAGCACTCTCATCGACAAGTACGACCTAAGCAACTTGCACGAGATCGCC

AGCGGCGGGGCGCCGCTCAGCAAGGAGGTAGGTGAGGCCGTGGCCAAACGCTTCCACCTACCAGGCATCCGCCAGGGCTACGGCCTGACAG

AAACAACCAGCGCCATTCTGATCACCCCCGAAGGGGACGACAAGCCTGGCGCAGTAGGCAAGGTGGTGCCCTTCTTCGAGGCTAAGGTGGT

GGACTTGGACACCGGTAAGACACTGGGTGTGAACCAGCGCGGCGAGCTGTGCGTCCGTGGCCCCATGATCATGAGCGGCTACGTTAACAAC

CCCGAGGCTACAAACGCTCTCATCGACAAGGACGGCTGGCTGCACAGCGGCGACATCGCCTACTGGGACGAGGACGAGCACTTCTTCATCG

TGGACCGGCTGAAGAGCCTGATCAAATACAAGGGCTACCAGGTAGCCCCAGCCGAACTGGAGAGCATCCTGCTGCAACACCCCAACATCTT

CGACGCCGGGGTCGCCGGCCTGCCCGACGACGATGCCGGCGAGCTGCCCGCCGCAGTCGTCGTGCTGGAACACGGTAAAACCATGACCGAG

AAGGAGATCGTGGACTATGTGGCCAGCCAGGTTACAACCGCCAAGAAGCTGCGCGGTGGTGTTGTGTTCGTGGACGAGGTGCCTAAAGGAC

TGACCGGCAAGTTGGACGCCCGCAAGATCCGCGAGATTCTCATTAAGGCCAAGAAGGGCGGCAAGATCGCCGTGTAATAATTCTAGAGTCG

GGGCGGCCGGCCGCTTCGAGCAGACATGATAAGATACATTGATGAGTTTGGACAAACCACAACTAGAATGCAGTGAAAAAAATGCTTTATT

TGTGAAATTTGTGATGCTATTGCTTTATTTGTAACCATTATAAGCTGCAATAAACAAGTTAACAACAACAATTGCATTCATTTTATGTTTC

AGGTTCAGGGGGAGGTGTGGGAGGTTTTTTAAAGCAAGTAAAACCTCTACAAATGTGGTAAAATCGATAAGGATCCGTCGACCGATGCCCT

TGAGAGCCTTCAACCCAGTCAGCTCCTTCCGGTGGGCGCGGGGCATGACTATCGTCGCCGCACTTATGACTGTCTTCTTTATCATGCAACT

CGTAGGACAGGTGCCGGCAGCGCTCTTCCGCTTCCTCGCTCACTGACTCGCTGCGCTCGGTCGTTCGGCTGCGGCGAGCGGTATCAGCTCA

CTCAAAGGCGGTAATACGGTTATCCACAGAATCAGGGGATAACGCAGGAAAGAACATGTGAGCAAAAGGCCAGCAAAAGGCCAGGAACCGT

AAAAAGGCCGCGTTGCTGGCGTTTTTCCATAGGCTCCGCCCCCCTGACGAGCATCACAAAAATCGACGCTCAAGTCAGAGGTGGCGAAACC

CGACAGGACTATAAAGATACCAGGCGTTTCCCCCTGGAAGCTCCCTCGTGCGCTCTCCTGTTCCGACCCTGCCGCTTACCGGATACCTGTC

CGCCTTTCTCCCTTCGGGAAGCGTGGCGCTTTCTCATAGCTCACGCTGTAGGTATCTCAGTTCGGTGTAGGTCGTTCGCTCCAAGCTGGGC

TGTGTGCACGAACCCCCCGTTCAGCCCGACCGCTGCGCCTTATCCGGTAACTATCGTCTTGAGTCCAACCCGGTAAGACACGACTTATCGC

CACTGGCAGCAGCCACTGGTAACAGGATTAGCAGAGCGAGGTATGTAGGCGGTGCTACAGAGTTCTTGAAGTGGTGGCCTAACTACGGCTA

CACTAGAAGAACAGTATTTGGTATCTGCGCTCTGCTGAAGCCAGTTACCTTCGGAAAAAGAGTTGGTAGCTCTTGATCCGGCAAACAAACC

ACCGCTGGTAGCGGTGGTTTTTTTGTTTGCAAGCAGCAGATTACGCGCAGAAAAAAAGGATCTCAAGAAGATCCTTTGATCTTTTCTACGG

GGTCTGACGCTCAGTGGAACGAAAACTCACGTTAAGGGATTTTGGTCATGAGATTATCAAAAAGGATCTTCACCTAGATCCTTTTAAATTA

AAAATGAAGTTTTAAATCAATCTAAAGTATATATGAGTAAACTTGGTCTGACAGCGGCCGCAAATGCTAAACCACTGCAGTGGTTACCAGT

GCTTGATCAGTGAGGCACCGATCTCAGCGATCTGCCTATTTCGTTCGTCCATAGTGGCCTGACTCCCCGTCGTGTAGATCACTACGATTCG

TGAGGGCTTACCATCAGGCCCCAGCGCAGCAATGATGCCGCGAGAGCCGCGTTCACCGGCCCCCGATTTGTCAGCAATGAACCAGCCAGCA

GGGAGGGCCGAGCGAAGAAGTGGTCCTGCTACTTTGTCCGCCTCCATCCAGTCTATGAGCTGCTGTCGTGATGCTAGAGTAAGAAGTTCGC

CAGTGAGTAGTTTCCGAAGAGTTGTGGCCATTGCTACTGGCATCGTGGTATCACGCTCGTCGTTCGGTATGGCTTCGTTCAACTCTGGTTC

CCAGCGGTCAAGCCGGGTCACATGATCACCCATATTATGAAGAAATGCAGTCAGCTCCTTAGGGCCTCCGATCGTTGTCAGAAGTAAGTTG

GCCGCGGTGTTGTCGCTCATGGTAATGGCAGCACTACACAATTCTCTTACCGTCATGCCATCCGTAAGATGCTTTTCCGTGACCGGCGAGT

ACTCAACCAAGTCGTTTTGTGAGTAGTGTATACGGCGACCAAGCTGCTCTTGCCCGGCGTCTATACGGGACAACACCGCGCCACATAGCAG

TACTTTGAAAGTGCTCATCATCGGGAATCGTTCTTCGGGGCGGAAAGACTCAAGGATCTTGCCGCTATTGAGATCCAGTTCGATATAGCCC

ACTCTTGCACCCAGTTGATCTTCAGCATCTTTTACTTTCACCAGCGTTTCGGGGTGTGCAAAAACAGGCAAGCAAAATGCCGCAAAGAAGG

GAATGAGTGCGACACGAAAATGTTGGATGCTCATACTCGTCCTTTTTCAATATTATTGAAGCATTTATCAGGGTTACTAGTACGTCTCTCA

AGGATAAGTAAGTAATATTAAGGTACGGGAGGTATTGGACAGGCCGCAATAAAATATCTTTATTTTCATTACATCTGTGTGTTGGTTTTTT

GTGTGAATCGATAGTACTAACATACGCTCTCCATCAAAACAAAACGAAACAAAACAAACTAGCAAAATAGGCTGTCCCCAGTGCAAGTGCA

GGTGCCAGAACATTTCTCT

The sequence derived from GRCh38 chr15:88,569,212-88,569,685

The sequence of Luc2 cDNA

The location of $(TTTG)_4$ is underlined.

pGL4-$(TTTG)_4$-HSVTKp

GGCCTAACTGGCCGGTACCTGAGCTCGCTAGCCTCGAGGATATCAAGATCTGGCCTCGGCGGCCACAATACAGCCCTGTGACACAAGTTTA
CCTACATAACGAACCTGCACATGTATCCCTGAACCTAAAATAAAAATTGAAAACAATAAATAAATACAATATGTCAACAAGAACATATGGG
AAGAACCATGCCCAAAATGAAATAGTACAAAATGGTCATGCTTGGGTAAGGGATTGTGGCTGATGTTTCTTTTGTTGCTTGTTTAAACAAA
TTGT*TTTTC***TTTGTTTGTTTGTTTG**TTTTTCTTTGAGACGGTCTCACTCCGTTGCCTAGGCTGGAGTGCAGTGGCGCAATCTTGGCTCACT
GCAACCTCTGCCTCCTGGGTTCAAGCAATTCTCCTGCCCCAGCCTACTGAGTAGCTAGGACTATGGGTACCCACCACCATGCCTGGCTAAT
TTTTGTATTTTTTAGTAGAGACGAGTTATGATCTCACCACTGTACTCTAGCCTGGGTAACAGAGCAAGACCCTGTCTTAAATGAGTCTTCG
GACCTCGCGGGGGCCGCTTAAGCGGTGGTTAGGGTTTGTCTGACGCGGGGGGAGGGGGAAGGAACGAAACACTCTCATTCGGAGGCGGCTC
GGGGTTTGGTCTTGGTGGCCACGGGCACGCAGAAGAGCGCCGCGATCCTCTTAAGCACCCCCCCGCCCTCCGTGGAGGCGGGGGTTTGGTC
GGCGGGTGGTAACTGGCGGGCCGCTGACTCGGGCGGGTCGCGCGCCCCAGAGTGTGACCTTTTCGGTCTGCTCGCAGACCCCCGGGCGGCG
CCGCCGCGGCGGCGACGGGCTCGCTGGGTCCTAGGCTCCATGGGGACCGTATACGTGGACAGGCTCTGGAGCATCCGCACGACTGCGGTGA
TATTACCGGAGACCTTCTGCGGGACGAGCCGGGTCACGCGGCTGACGCGGAGCGTCCGTTGGGCGACAAACACCAGGACGGGGCACAGGTA
CACTATCTTGTCACCCGGAGGCGCGAGGGACTGCAGGAGCTTCAGGGAGTGGCGCAGCTGCTTCATCCCCGTGGCCCGTTGCTCGCGTTTG
CTGGCGGTGTCCCCGGAAGAAATATATTTGCATGTCTTTAGTTCTATGATGACACAAACCCCGCCCAGCGTCTTGTCATTGGCGAAGTCGA
ACACGCAGATGCAGTCGGGGCGGCGCGGTCCCAGGTCCACTTCGCATATTAAGGTGACGCGTGTGGCCTCGAACACCGAGCGACCCTGCAG
CGACCCGCTTAACTTGGCAATCCGGTACTGTTGGTAAAGCCACCATGGAAGATGCCAAAAACATTAAGAAGGGCCCAGCGCCATTCTACCC
ACTCGAAGACGGGACCGCCGGCGAGCAGCTGCACAAAGCCATGAAGCGCTACGCCCTGGTGCCCGGCACCATCGCCTTTACCGACGCACAT
ATCGAGGTGGACATTACCTACGCCGAGTACTTCGAGATGAGCGTTCGGCTGGCAGAAGCTATGAAGCGCTATGGGCTGAATACAAACCATC
GGATCGTGGTGTGCAGCGAGAATAGCTTGCAGTTCTTCATGCCCGTGTTGGGTGCCCTGTTCATCGGTGTGGCTGTGGCCCCAGCTAACGA
CATCTACAACGAGCGCGAGCTGCTGAACAGCATGGGCATCAGCCAGCCCACCGTCGTATTCGTGAGCAAGAAAGGGCTGCAAAAGATCCTC
AACGTGCAAAAGAAGCTACCGATCATACAAAAGATCATCATCATGGATAGCAAGACCGACTACCAGGGCTTCCAAAGCATGTACACCTTCG
TGACTTCCCATTTGCCACCCGGCTTCAACGAGTACGACTTCGTGCCCGAGAGCTTCGACCGGGACAAAACCATCGCCCTGATCATGAACAG
TAGTGGCAGTACCGGATTGCCCAAGGGCGTAGCCCTACCGCACCGCACCGCTTGTGTCCGATTCAGTCATGCCCGCGACCCCATCTTCGGC
AACCAGATCATCCCCGACACCGCTATCCTCAGCGTGGTGCCATTTCACCACGGCTTCGGCATGTTCACCACGCTGGGCTACTTGATCTGCG
GCTTTCGGGTCGTGCTCATGTACCGCTTCGAGGAGGAGCTATTCTTGCGCAGCTTGCAAGACTATAAGATTCAATCTGCCCTGCTGGTGCC
CACACTATTTAGCTTCTTCGCTAAGAGCACTCTCATCGACAAGTACGACCTAAGCAACTTGCACGAGATCGCCAGCGGCGGGGCGCCGCTC
AGCAAGGAGGTAGGTGAGGCCGTGGCCAAACGCTTCCACCTACCAGGCATCCGCCAGGGCTACGGCCTGACAGAAACAACCAGCGCCATTC
TGATCACCCCCGAAGGGGACGACAAGCCTGGCGCAGTAGGCAAGGTGGTGCCCTTCTTCGAGGCTAAGGTGGTGGACTTGGACACCGGTAA
GACACTGGGTGTGAACCAGCGCGGCGAGCTGTGCGTCCGTGGCCCCATGATCATGAGCGGCTACGTTAACAACCCCGAGGCTACAAACGCT
CTCATCGACAAGGACGGCTGGCTGCACAGCGGCGACATCGCCTACTGGGACGAGGACGAGCACTTCTTCATCGTGGACCGGCTGAAGAGCC
TGATCAAATACAAGGGCTACCAGGTAGCCCCAGCCGAACTGGAGAGCATCCTGCTGCAACACCCCAACATCTTCGACGCCGGGGTCGCCGG
CCTGCCCGACGACGATGCCGGCGAGCTGCCCGCCGCAGTCGTCGTGCTGGAACACGGTAAAACCATGACCGAGAAGGAGATCGTGGACTAT
GTGGCCAGCCAGGTTACAACCGCCAAGAAGCTGCGCGGTGGTGTTGTGTTCGTGGACGAGGTGCCTAAAGGACTGACCGGCAAGTTGGACG
CCCGCAAGATCCGCGAGATTCTCATTAAGGCCAAGAAGGGCGGCAAGATCGCCGTGTAATAATTCTAGAGTCGGGGCGGCCGGCCGCTTCG
AGCAGACATGATAAGATACATTGATGAGTTTGGACAAACCACAACTAGAATGCAGTGAAAAAAATGCTTTATTTGTGAAATTTGTGATGCT
ATTGCTTTATTTGTAACCATTATAAGCTGCAATAAACAAGTTAACAACAACAATTGCATTCATTTTATGTTTCAGGTTCAGGGGGAGGTGT
GGGAGGTTTTTTAAAGCAAGTAAAACCTCTACAAATGTGGTAAAATCGATAAGGATCCGTCGACCGATGCCCTTGAGAGCCTTCAACCCAG
TCAGCTCCTTCCGGTGGGCGCGGGGCATGACTATCGTCGCCGCACTTATGACTGTCTTCTTTATCATGCAACTCGTAGGACAGGTGCCGGC

AGCGCTCTTCCGCTTCCTCGCTCACTGACTCGCTGCGCTCGGTCGTTCGGCTGCGGCGAGCGGTATCAGCTCACTCAAAGGCGGTAATACG
GTTATCCACAGAATCAGGGGATAACGCAGGAAAGAACATGTGAGCAAAAGGCCAGCAAAAGGCCAGGAACCGTAAAAAGGCCGCGTTGCTG
GCGTTTTTCCATAGGCTCCGCCCCCCTGACGAGCATCACAAAAATCGACGCTCAAGTCAGAGGTGGCGAAACCCGACAGGACTATAAAGAT
ACCAGGCGTTTCCCCCTGGAAGCTCCCTCGTGCGCTCTCCTGTTCCGACCCTGCCGCTTACCGGATACCTGTCCGCCTTTCTCCCTTCGGG
AAGCGTGGCGCTTTCTCATAGCTCACGCTGTAGGTATCTCAGTTCGGTGTAGGTCGTTCGCTCCAAGCTGGGCTGTGTGCACGAACCCCCC
GTTCAGCCCGACCGCTGCGCCTTATCCGGTAACTATCGTCTTGAGTCCAACCCGGTAAGACACGACTTATCGCCACTGGCAGCAGCCACTG
GTAACAGGATTAGCAGAGCGAGGTATGTAGGCGGTGCTACAGAGTTCTTGAAGTGGTGGCCTAACTACGGCTACACTAGAAGAACAGTATT
TGGTATCTGCGCTCTGCTGAAGCCAGTTACCTTCGGAAAAAGAGTTGGTAGCTCTTGATCCGGCAAACAAACCACCGCTGGTAGCGGTGGT
TTTTTTGTTTGCAAGCAGCAGATTACGCGCAGAAAAAAAGGATCTCAAGAAGATCCTTTGATCTTTTCTACGGGGTCTGACGCTCAGTGGA
ACGAAAACTCACGTTAAGGGATTTTGGTCATGAGATTATCAAAAAGGATCTTCACCTAGATCCTTTTAAATTAAAAATGAAGTTTTAAATC
AATCTAAAGTATATATGAGTAAACTTGGTCTGACAGCGGCCGCAAATGCTAAACCACTGCAGTGGTTACCAGTGCTTGATCAGTGAGGCAC
CGATCTCAGCGATCTGCCTATTTCGTTCGTCCATAGTGGCCTGACTCCCCGTCGTGTAGATCACTACGATTCGTGAGGGCTTACCATCAGG
CCCCAGCGCAGCAATGATGCCGCGAGAGCCGCGTTCACCGGCCCCCGATTTGTCAGCAATGAACCAGCCAGCAGGGAGGGCCGAGCGAAGA
AGTGGTCCTGCTACTTTGTCCGCCTCCATCCAGTCTATGAGCTGCTGTCGTGATGCTAGAGTAAGAAGTTCGCCAGTGAGTAGTTTCCGAA
GAGTTGTGGCCATTGCTACTGGCATCGTGGTATCACGCTCGTCGTTCGGTATGGCTTCGTTCAACTCTGGTTCCCAGCGGTCAAGCCGGGT
CACATGATCACCCATATTATGAAGAAATGCAGTCAGCTCCTTAGGGCCTCCGATCGTTGTCAGAAGTAAGTTGGCCGCGGTGTTGTCGCTC
ATGGTAATGGCAGCACTACACAATTCTCTTACCGTCATGCCATCCGTAAGATGCTTTTCCGTGACCGGCGAGTACTCAACCAAGTCGTTTT
GTGAGTAGTGTATACGGCGACCAAGCTGCTCTTGCCCGGCGTCTATACGGGACAACACCGCGCCACATAGCAGTACTTTGAAAGTGCTCAT
CATCGGGAATCGTTCTTCGGGGCGGAAAGACTCAAGGATCTTGCCGCTATTGAGATCCAGTTCGATATAGCCCACTCTTGCACCCAGTTGA
TCTTCAGCATCTTTTACTTTCACCAGCGTTTCGGGGTGTGCAAAAACAGGCAAGCAAAATGCCGCAAAGAAGGGAATGAGTGCGACACGAA
AATGTTGGATGCTCATACTCGTCCTTTTTCAATATTATTGAAGCATTTATCAGGGTTACTAGTACGTCTCTCAAGGATAAGTAAGTAATAT
TAAGGTACGGGAGGTATTGGACAGGCCGCAATAAAATATCTTTATTTTCATTACATCTGTGTGTTGGTTTTTTGTGTGAATCGATAGTACT
AACATACGCTCTCCATCAAAACAAAACGAAACAAAACAAACTAGCAAAATAGGCTGTCCCCAGTGCAAGTGCAGGTGCCAGAACATTTCTC
T

The sequence derived from GRCh38 chr15:88,569,212-88,569,685

The sequence of HSVTK promoter

The sequence of Luc2 cDNA

The location of $(TTTG)_4$ is underlined.

Supplementary Fig. 1

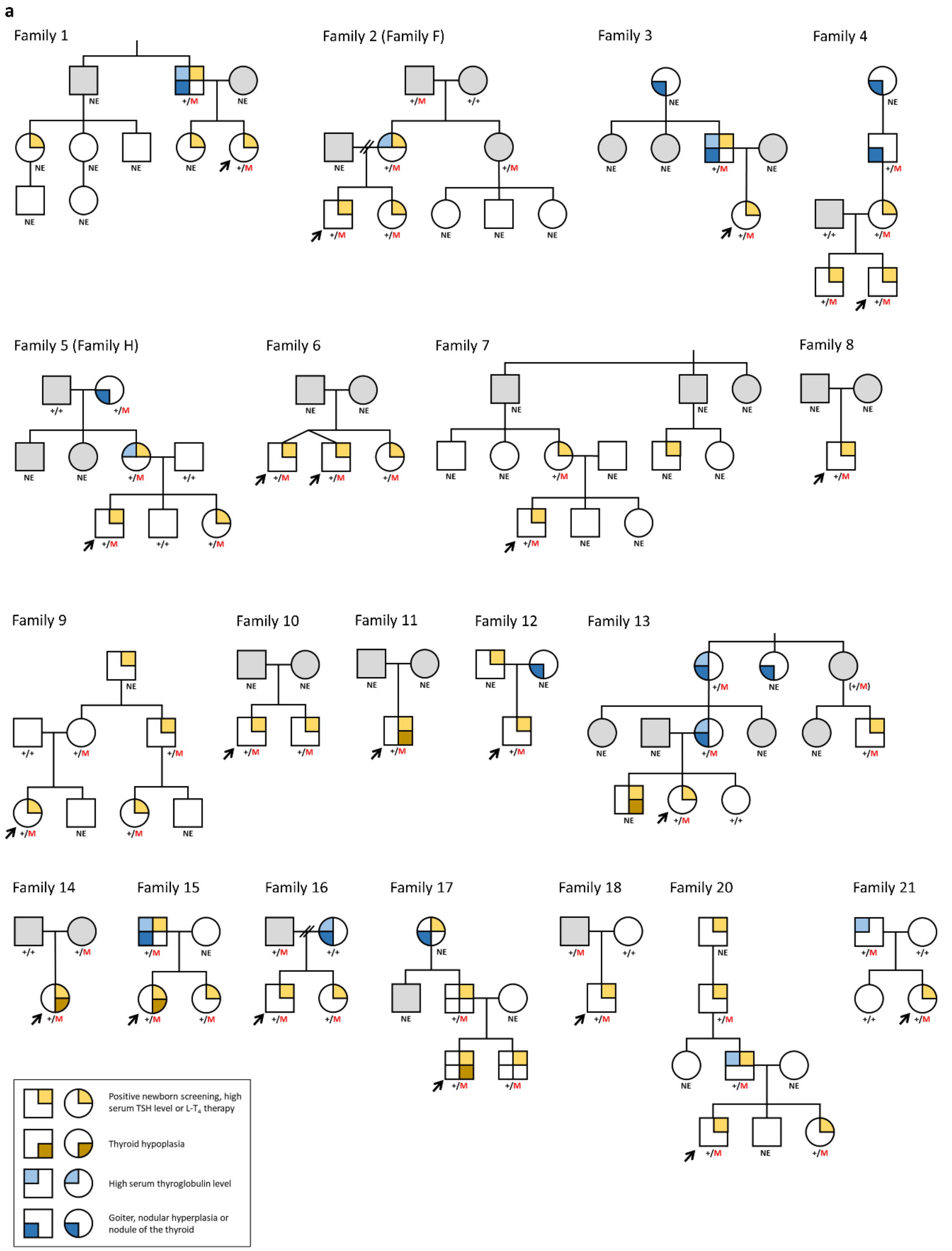

Supplementary Fig. 1 (continued)

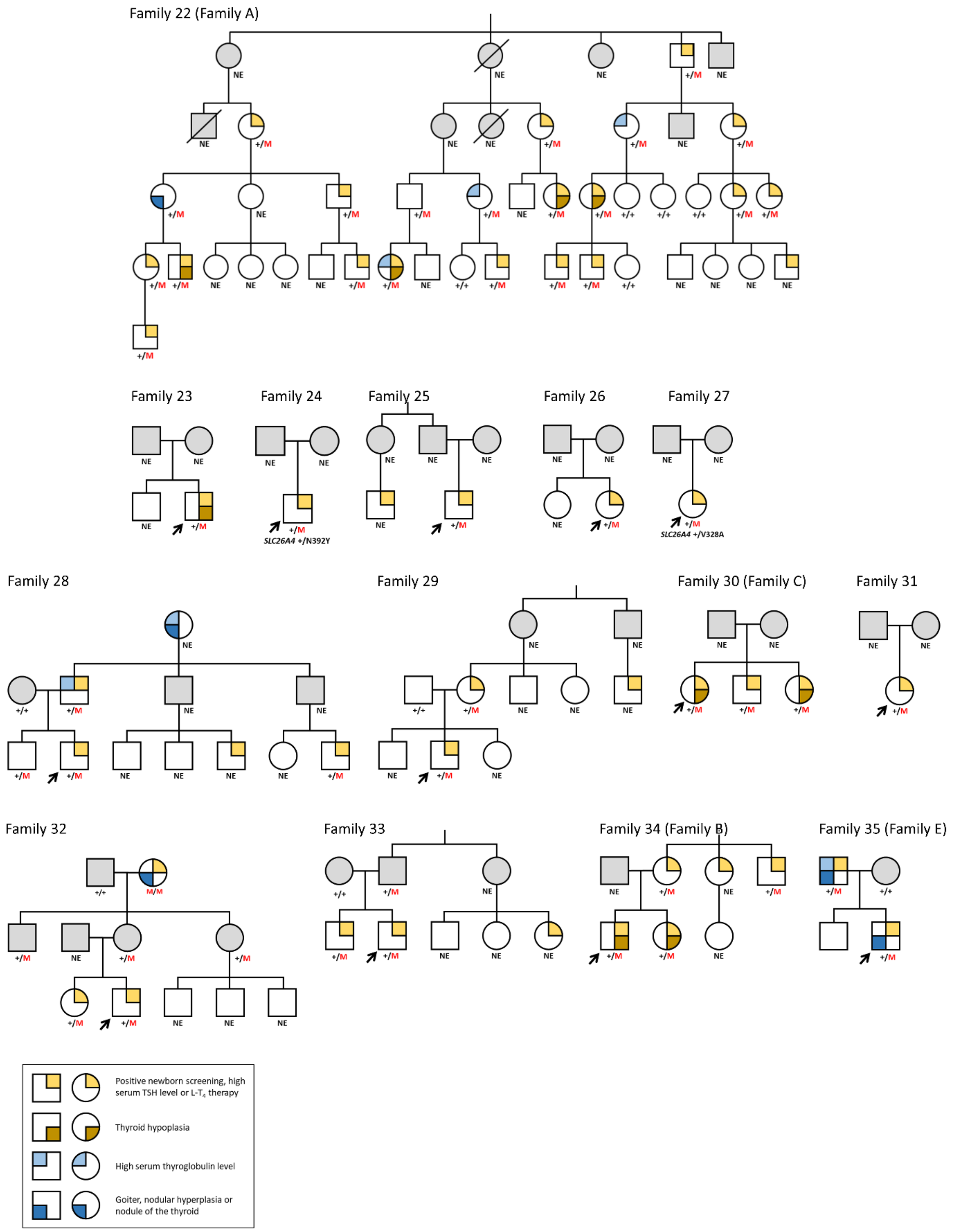

Supplementary Fig. 1 (continued)

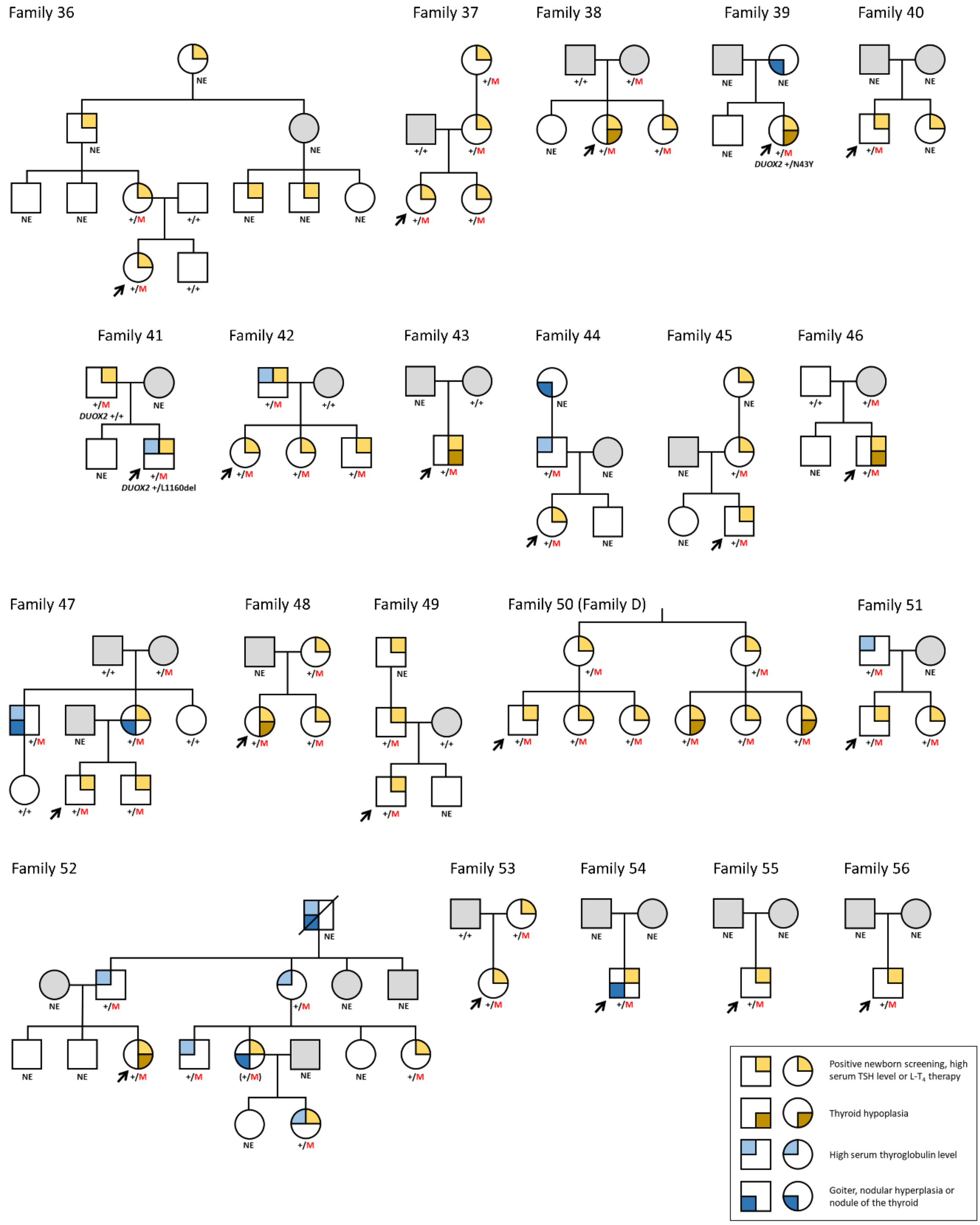

Supplementary Fig. 1 (continued)

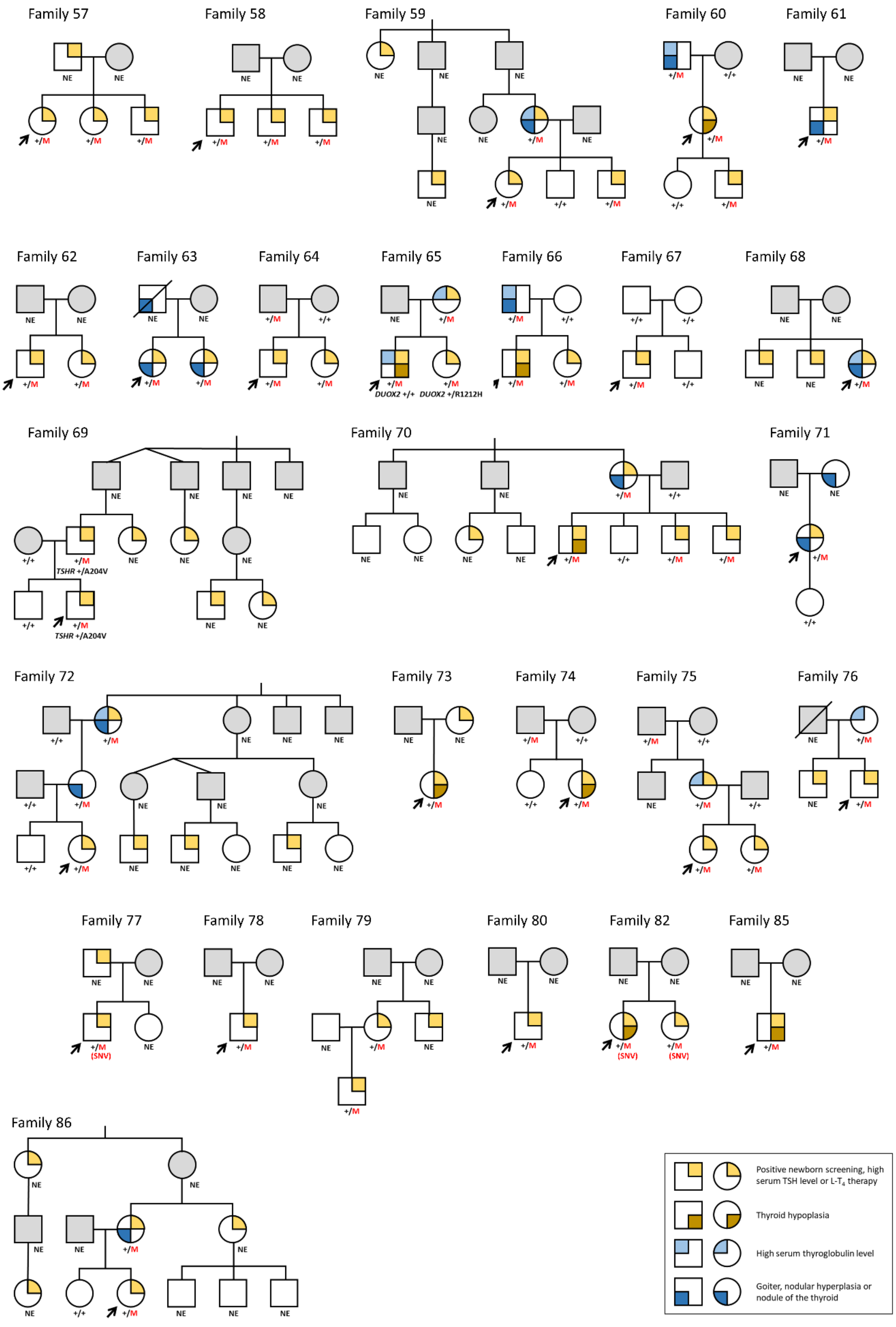

Supplementary Fig. 1 (continued)

b

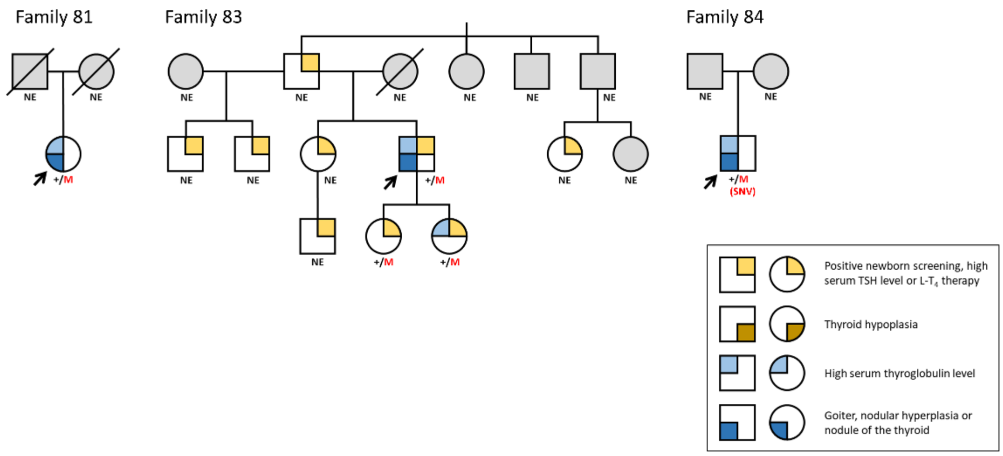

Pedigrees of families with (TTTG)₃ or single nucleotide variant (SNV). a, Families with (TTTG)₃ or SNV, whose probands were congenital hypothyroidism (CH) patients. **b,** Those whose probands were multinodular goiter (MNG) patients.

Supplementary Fig. 2

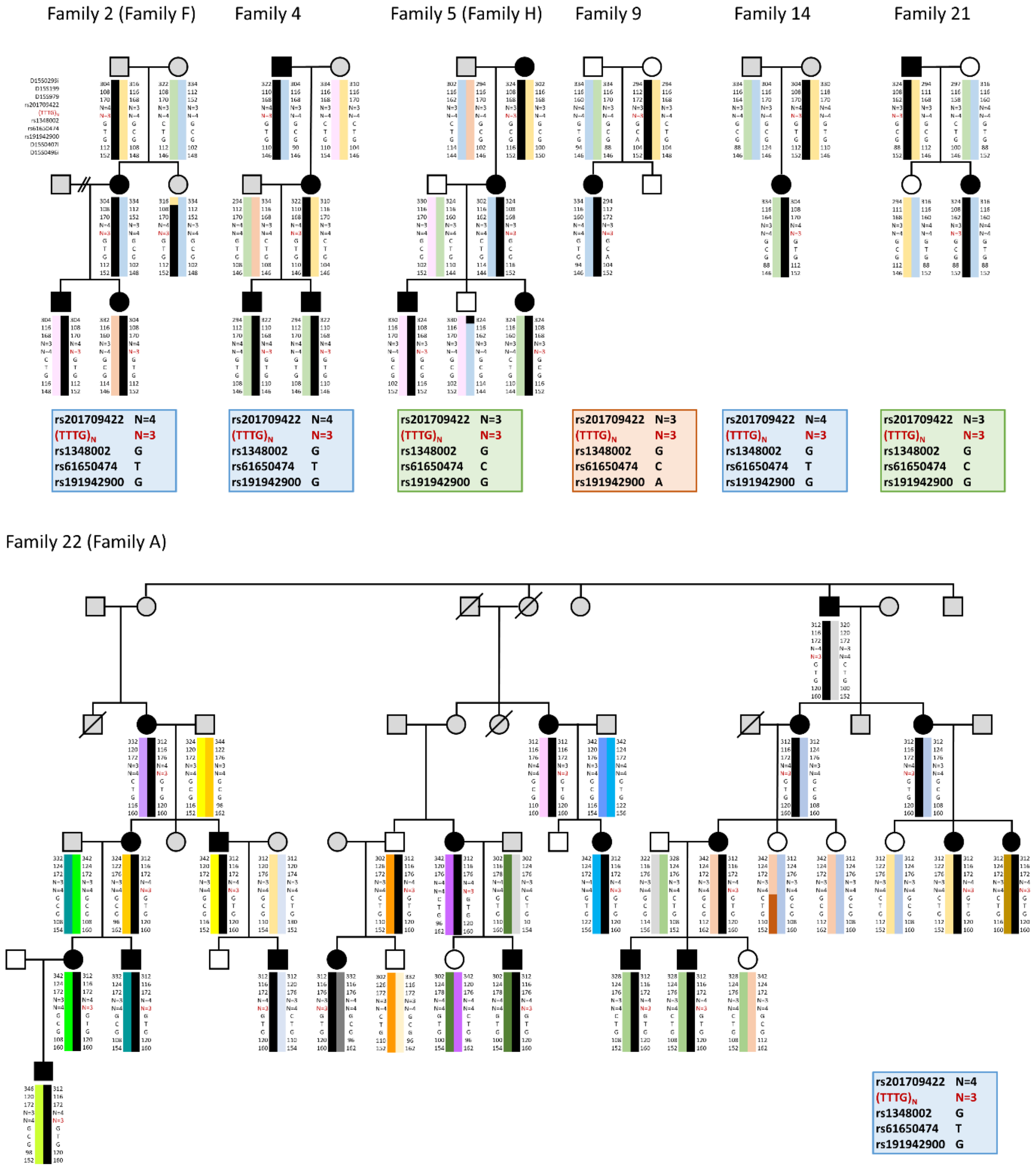

Supplementary Fig. 2 (continued)

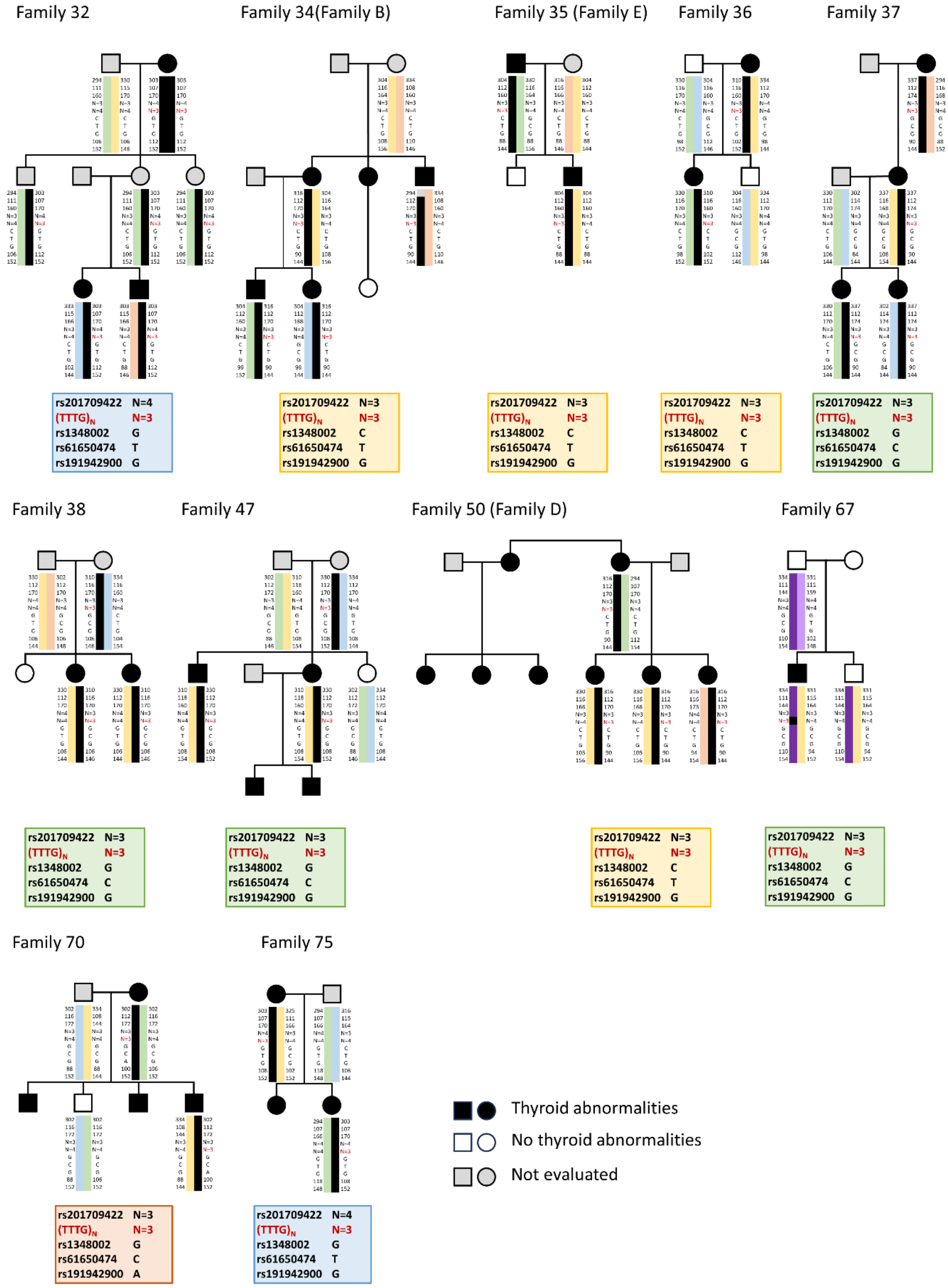

Haplotype analysis of families with $(TTTG)_3$. Genetic markers [short tandem repeats or single nucleotide polymorphisms (SNPs)] are ordered from centromere to telomere. The inherited portion of the disease-associated haplotype is indicated by blackened bars. Four distinct haplotypes define by four polymorphic markers (rs201709422, rs1348002, rs61650474 and rs191942900) that were segregated with $(TTTG)_3$ were identified (colored in blue, green, orange or yellow).

Supplementary Fig. 3

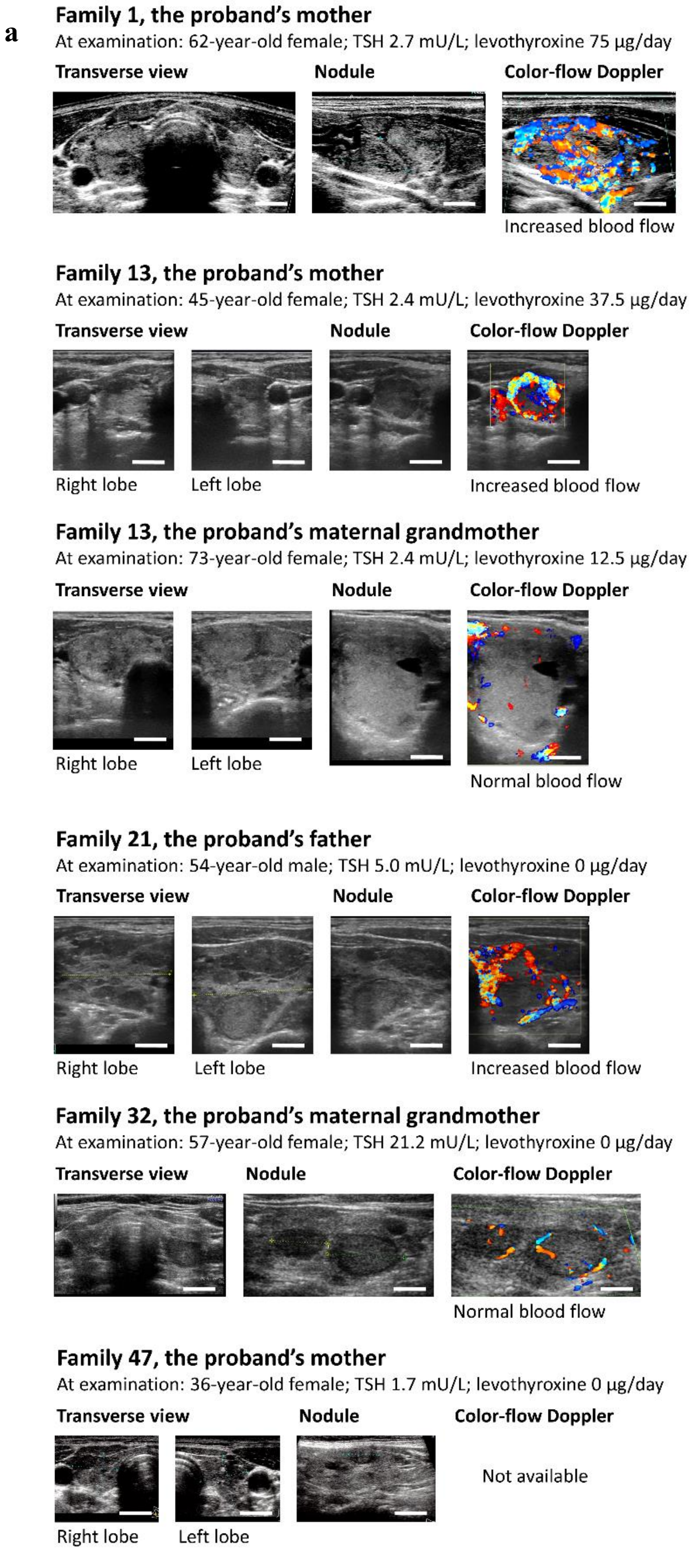

Supplementary Fig. 3 (continued)

Family 47, the proband's maternal uncle

At examination: 29-year-old male; TSH 3.0 mU/L; levothyroxine 0 μg/day

Transverse view **Nodule** **Color-flow Doppler**

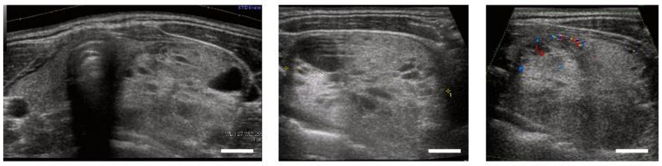

Normal blood flow

Family 59, the proband's mother

At examination: 32-year-old female; TSH 4.8 mU/L; levothyroxine 0 μg/day

Transverse view **Nodule** **Color-flow Doppler**

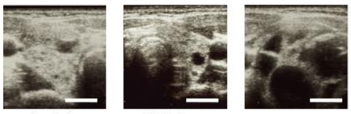

Not available

Right lobe Left lobe

Family 60, the proband's father

At examination: 64-year-old male; TSH 3.7 mU/L; levothyroxine 0 μg/day

Transverse view **Nodule** **Color-flow Doppler**

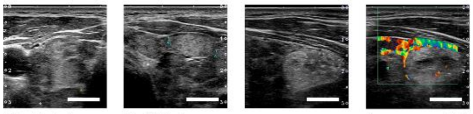

Right lobe Left lobe Increased blood flow

Family 66, the proband's father

At examination: 51-year-old male; TSH 3.6 mU/L; levothyroxine 0 μg/day

Transverse view **Nodule** **Color-flow Doppler**

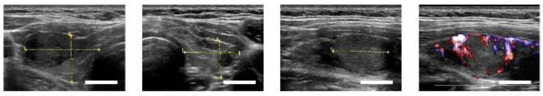

Right lobe Left lobe Increased blood flow

Family 72, the proband's maternal grandmother

At examination: 72-year-old female; TSH 5.8 mU/L; levothyroxine 12.5 μg/day

Transverse view **Nodule** **Color-flow Doppler**

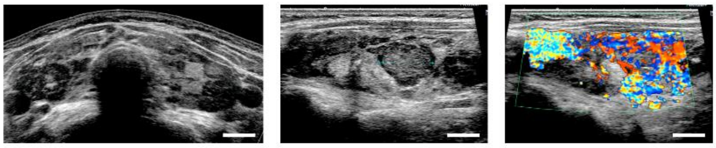

Increased blood flow

Family 76, the proband's mother

At examination: 57-year-old female; TSH 2.1 mU/L; levothyroxine 0 μg/day

Transverse view **Nodule** **Color-flow Doppler**

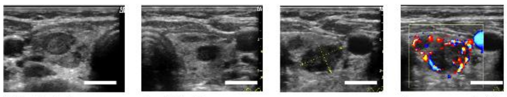

Right lobe Left lobe Increased blood flow

Family 86, the proband's mother

At examination: 40-year-old female; TSH 4.4 mU/L; levothyroxine 25 μg/day

Transverse view **Nodule** **Color-flow Doppler**

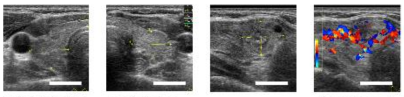

Right lobe Left lobe Increased blood flow

Supplementary Fig. 3 (continued)

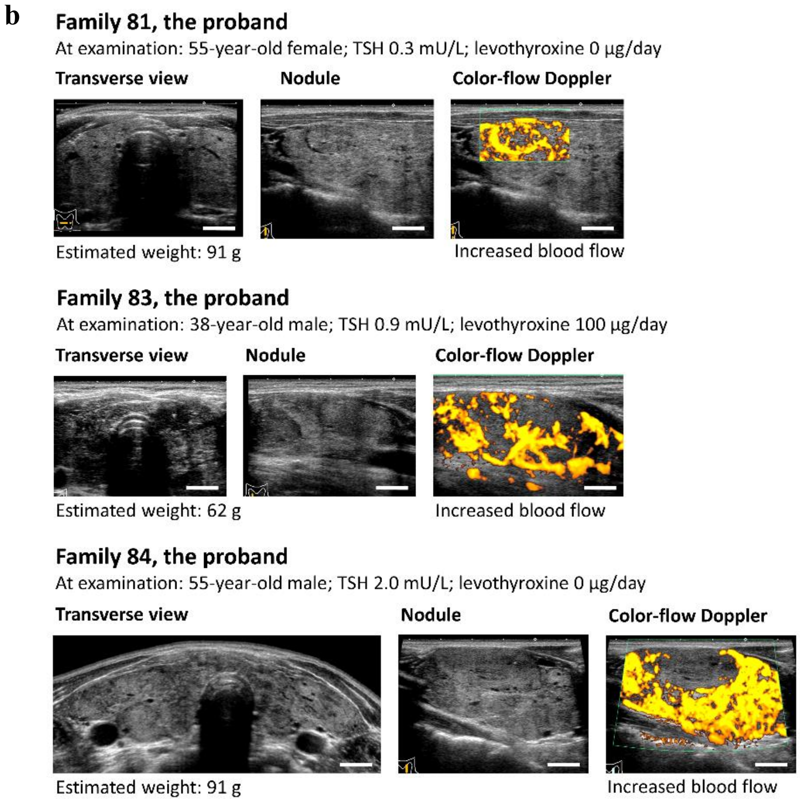

Ultrasonographic images of adult individuals born before the implementation of newborn screening. a, Adult individuals with $(TTTG)_3$, who were identified through family analysis of the congenital hypothyroidism (CH) probands. **b,** Adult individuals with $(TTTG)_3$ or single nucleotide variant (SNV), who were identified by a genetic screen in the multinodular goiter (MNG) patient cohort. Bars indicate 1 cm.

Supplementary Fig. 4

a

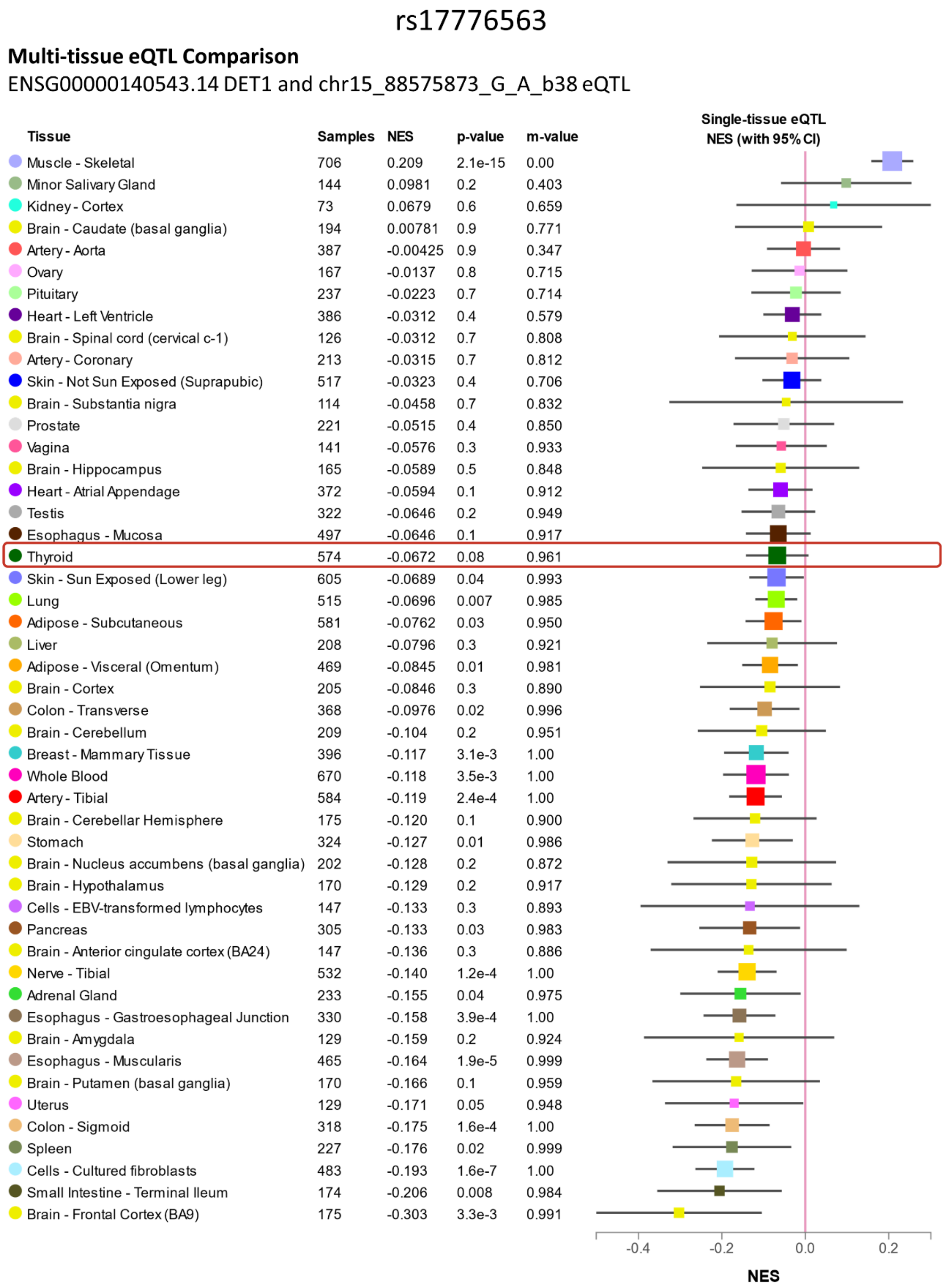

rs17776563

Multi-tissue eQTL Comparison

ENSG00000140543.14 DET1 and chr15_88575873_G_A_b38 eQTL

Tissue	Samples	NES	p-value	m-value
Muscle - Skeletal	706	0.209	2.1e-15	0.00
Minor Salivary Gland	144	0.0981	0.2	0.403
Kidney - Cortex	73	0.0679	0.6	0.659
Brain - Caudate (basal ganglia)	194	0.00781	0.9	0.771
Artery - Aorta	387	-0.00425	0.9	0.347
Ovary	167	-0.0137	0.8	0.715
Pituitary	237	-0.0223	0.7	0.714
Heart - Left Ventricle	386	-0.0312	0.4	0.579
Brain - Spinal cord (cervical c-1)	126	-0.0312	0.7	0.808
Artery - Coronary	213	-0.0315	0.7	0.812
Skin - Not Sun Exposed (Suprapubic)	517	-0.0323	0.4	0.706
Brain - Substantia nigra	114	-0.0458	0.7	0.832
Prostate	221	-0.0515	0.4	0.850
Vagina	141	-0.0576	0.3	0.933
Brain - Hippocampus	165	-0.0589	0.5	0.848
Heart - Atrial Appendage	372	-0.0594	0.1	0.912
Testis	322	-0.0646	0.2	0.949
Esophagus - Mucosa	497	-0.0646	0.1	0.917
Thyroid	574	-0.0672	0.08	0.961
Skin - Sun Exposed (Lower leg)	605	-0.0689	0.04	0.993
Lung	515	-0.0696	0.007	0.985
Adipose - Subcutaneous	581	-0.0762	0.03	0.950
Liver	208	-0.0796	0.3	0.921
Adipose - Visceral (Omentum)	469	-0.0845	0.01	0.981
Brain - Cortex	205	-0.0846	0.3	0.890
Colon - Transverse	368	-0.0976	0.02	0.996
Brain - Cerebellum	209	-0.104	0.2	0.951
Breast - Mammary Tissue	396	-0.117	3.1e-3	1.00
Whole Blood	670	-0.118	3.5e-3	1.00
Artery - Tibial	584	-0.119	2.4e-4	1.00
Brain - Cerebellar Hemisphere	175	-0.120	0.1	0.900
Stomach	324	-0.127	0.01	0.986
Brain - Nucleus accumbens (basal ganglia)	202	-0.128	0.2	0.872
Brain - Hypothalamus	170	-0.129	0.2	0.917
Cells - EBV-transformed lymphocytes	147	-0.133	0.3	0.893
Pancreas	305	-0.133	0.03	0.983
Brain - Anterior cingulate cortex (BA24)	147	-0.136	0.3	0.886
Nerve - Tibial	532	-0.140	1.2e-4	1.00
Adrenal Gland	233	-0.155	0.04	0.975
Esophagus - Gastroesophageal Junction	330	-0.158	3.9e-4	1.00
Brain - Amygdala	129	-0.159	0.2	0.924
Esophagus - Muscularis	465	-0.164	1.9e-5	0.999
Brain - Putamen (basal ganglia)	170	-0.166	0.1	0.959
Uterus	129	-0.171	0.05	0.948
Colon - Sigmoid	318	-0.175	1.6e-4	1.00
Spleen	227	-0.176	0.02	0.999
Cells - Cultured fibroblasts	483	-0.193	1.6e-7	1.00
Small Intestine - Terminal Ileum	174	-0.206	0.008	0.984
Brain - Frontal Cortex (BA9)	175	-0.303	3.3e-3	0.991

Supplementary Fig. 4 (continued)

b

rs1348005

Multi-tissue eQTL Comparison

ENSG00000140543.14 DET1 and chr15_88570251_A_G_b38 eQTL

Tissue	Samples	NES	p-value	m-value
Muscle - Skeletal	706	0.189	1.4e-14	0.00
Brain - Caudate (basal ganglia)	194	0.0530	0.5	0.762
Kidney - Cortex	73	0.0361	0.8	0.797
Minor Salivary Gland	144	0.0336	0.7	0.743
Artery - Aorta	387	0.00283	0.9	0.475
Pituitary	237	-0.0125	0.8	0.734
Testis	322	-0.0136	0.8	0.734
Ovary	167	-0.0151	0.8	0.781
Brain - Spinal cord (cervical c-1)	126	-0.0236	0.8	0.856
Heart - Left Ventricle	386	-0.0390	0.2	0.850
Prostate	221	-0.0413	0.5	0.917
Artery - Coronary	213	-0.0461	0.5	0.897
Brain - Substantia nigra	114	-0.0463	0.7	0.890
Brain - Hippocampus	165	-0.0497	0.6	0.891
Skin - Sun Exposed (Lower leg)	605	-0.0504	0.1	0.961
Heart - Atrial Appendage	372	-0.0533	0.2	0.956
Skin - Not Sun Exposed (Suprapubic)	517	-0.0550	0.1	0.941
Lung	515	-0.0567	0.02	1.00
Adipose - Subcutaneous	581	-0.0587	0.06	0.963
Vagina	141	-0.0589	0.3	0.956
Esophagus - Mucosa	497	-0.0676	0.06	0.993
Colon - Transverse	368	-0.0759	0.05	0.978
Thyroid	574	-0.0795	0.03	0.993
Liver	208	-0.0876	0.2	0.904
Brain - Cortex	205	-0.0910	0.3	0.978
Pancreas	305	-0.0948	0.09	0.980
Whole Blood	670	-0.101	0.007	0.999
Breast - Mammary Tissue	396	-0.103	0.005	1.00
Adipose - Visceral (Omentum)	469	-0.105	1.2e-3	0.991
Brain - Cerebellar Hemisphere	175	-0.109	0.1	0.980
Brain - Cerebellum	209	-0.110	0.2	0.969
Spleen	227	-0.110	0.1	0.941
Adrenal Gland	233	-0.111	0.09	0.932
Artery - Tibial	584	-0.116	1.1e-4	1.00
Stomach	324	-0.117	0.009	0.996
Brain - Hypothalamus	170	-0.119	0.2	0.912
Cells - EBV-transformed lymphocytes	147	-0.122	0.3	0.904
Nerve - Tibial	532	-0.127	1.0e-4	1.00
Brain - Nucleus accumbens (basal ganglia)	202	-0.129	0.2	0.946
Esophagus - Muscularis	465	-0.134	1.7e-4	1.00
Esophagus - Gastroesophageal Junction	330	-0.144	4.3e-4	1.00
Uterus	129	-0.147	0.08	0.983
Brain - Anterior cingulate cortex (BA24)	147	-0.154	0.2	0.937
Small Intestine - Terminal Ileum	174	-0.159	0.02	0.989
Cells - Cultured fibroblasts	483	-0.168	9.2e-7	1.00
Colon - Sigmoid	318	-0.168	9.9e-5	1.00
Brain - Amygdala	129	-0.193	0.08	0.955
Brain - Putamen (basal ganglia)	170	-0.206	0.03	0.943
Brain - Frontal Cortex (BA9)	175	-0.357	4.7e-4	0.973

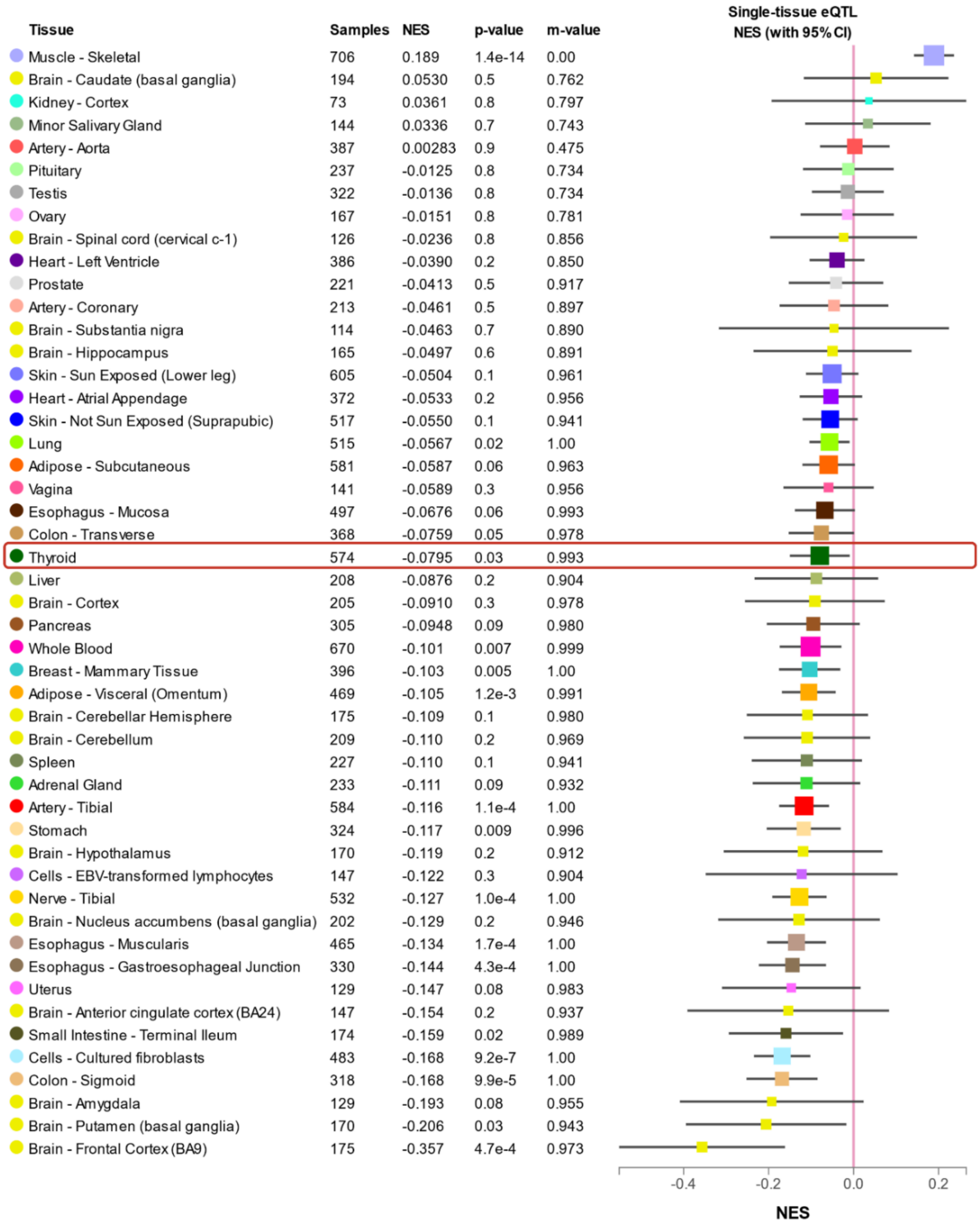

rs17776563 and rs1348005 as expression quantitative trait loci (eQTLs). Top-hit single nucleotide polymorphisms (SNPs) of genome-wide association studies (GWAS) on thyroid function (**a,** rs17776563; and **b,** rs1348005) are eQTLs affecting thyroidal *DET1* expression (shown in red boxes). Note that risk genotypes (rs17776563 G and rs1348005 A) for low thyroid function were associated with higher thyroidal *DET1* expression. Data are normalized effect size (boxes) and 95% confidence interval (bars). P values were calculated with two-sided t-test that compares observed normalized effect size (NES) from single-tissue eQTL analysis to a null NES of 0, without adjustment for multiple comparisons.

Source data

Family 1, the proband's mother

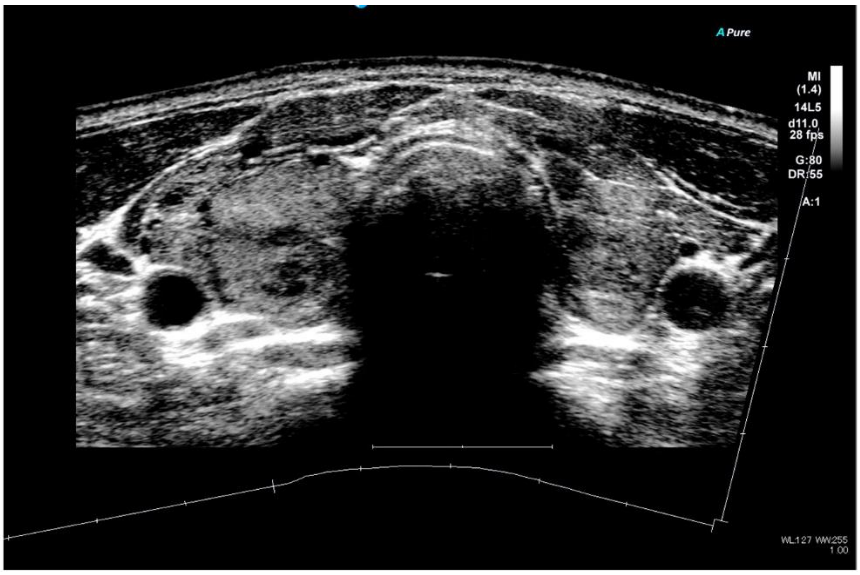

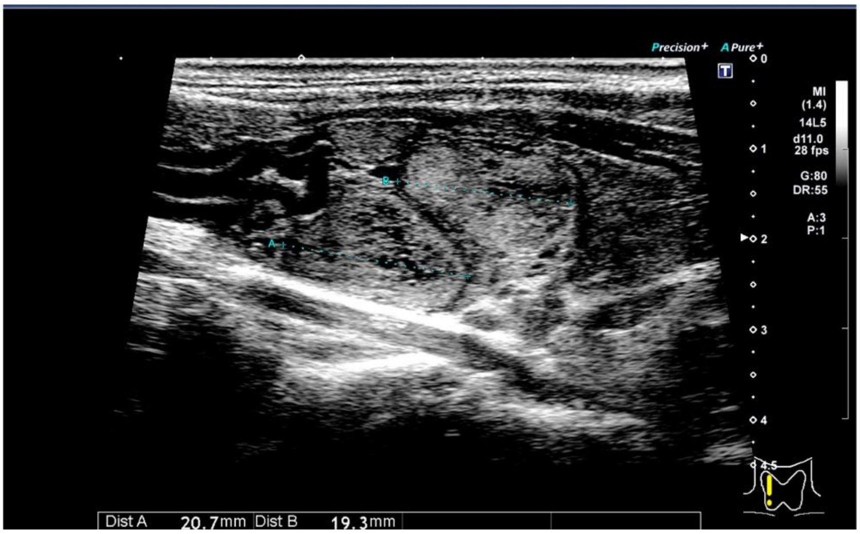

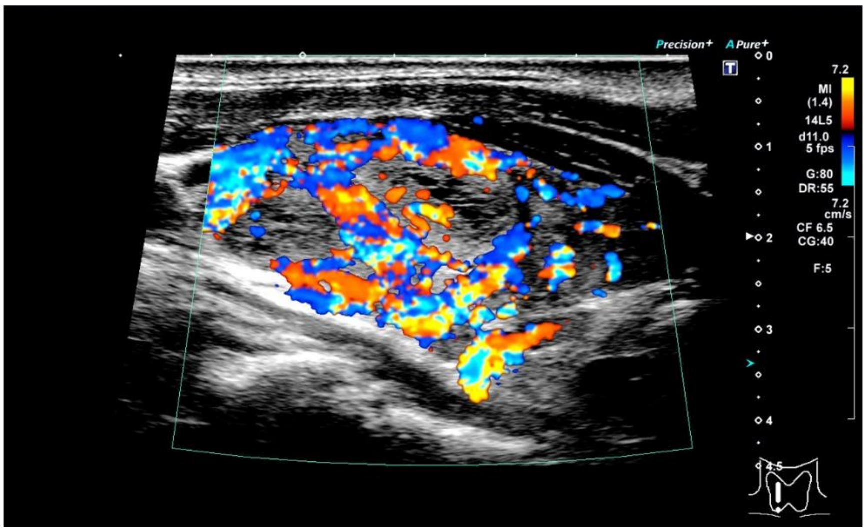

Family 13, the proband's mother

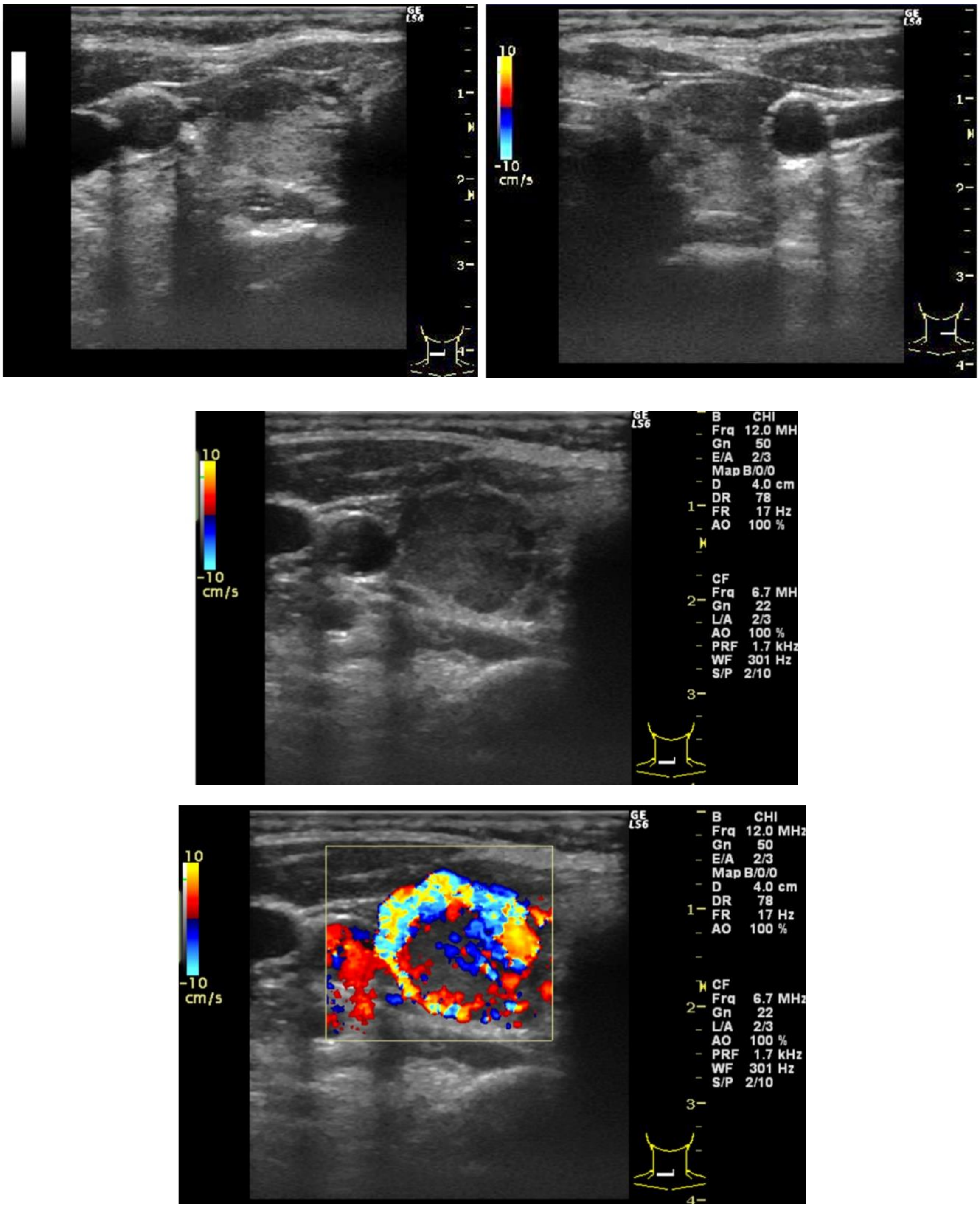

Family 13, the proband's grandmother

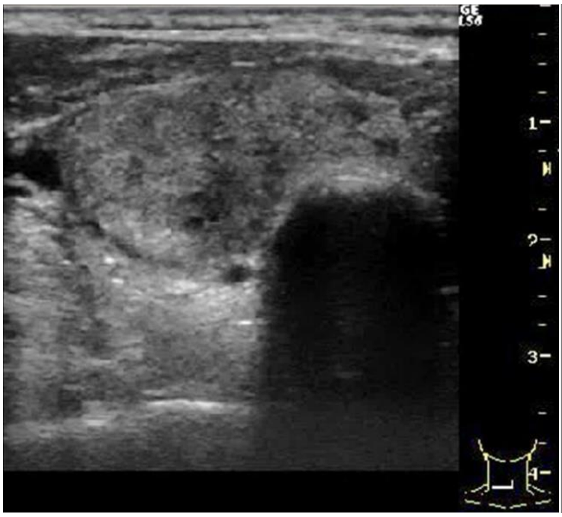

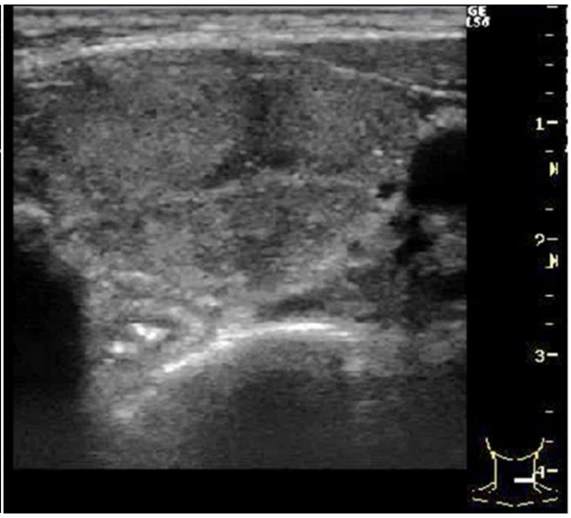

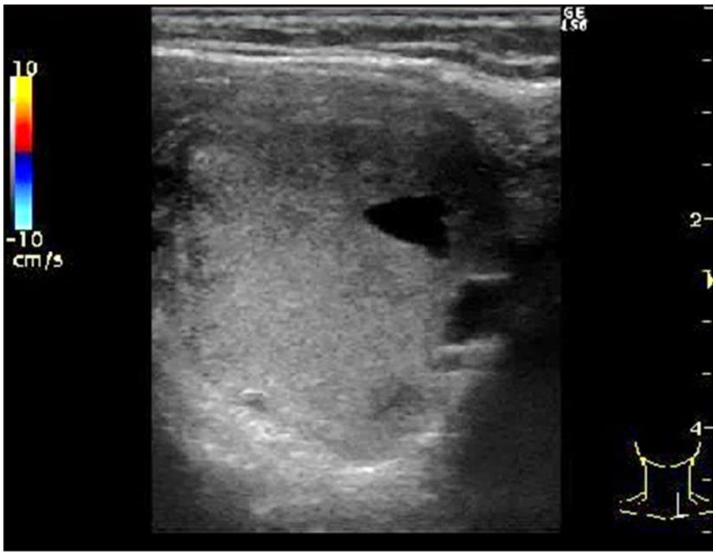

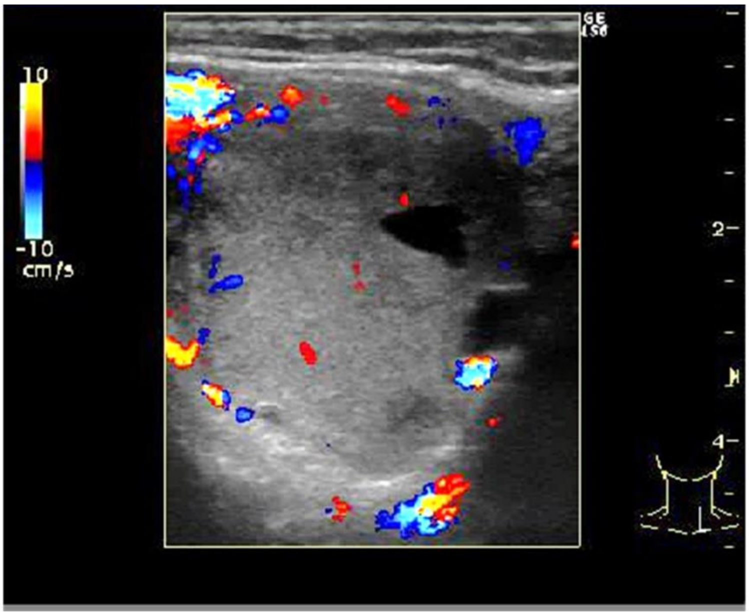

Family 21, the proband's father

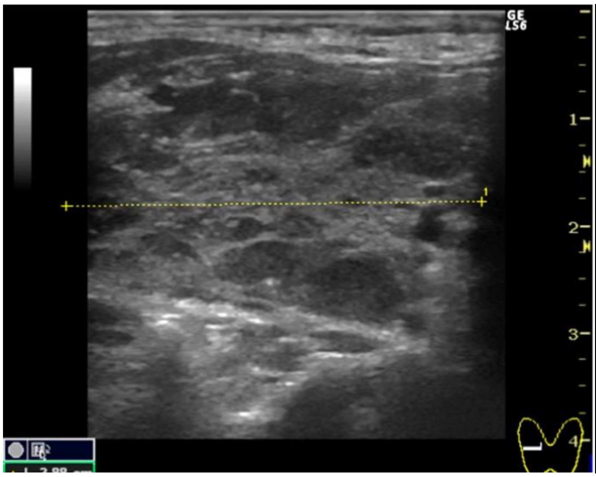

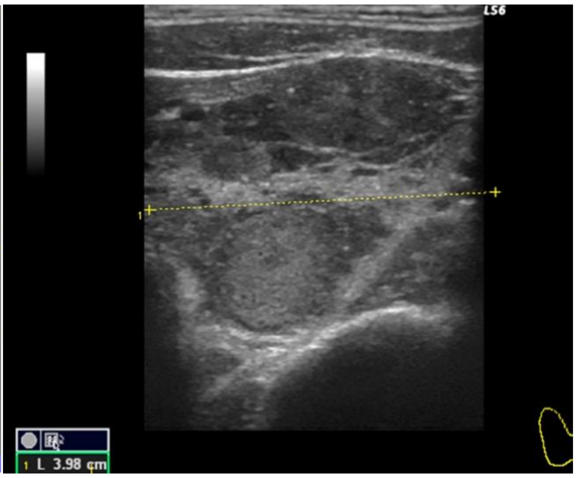

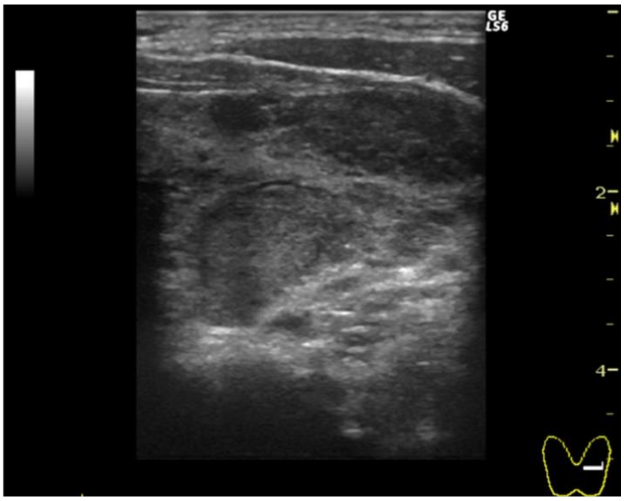

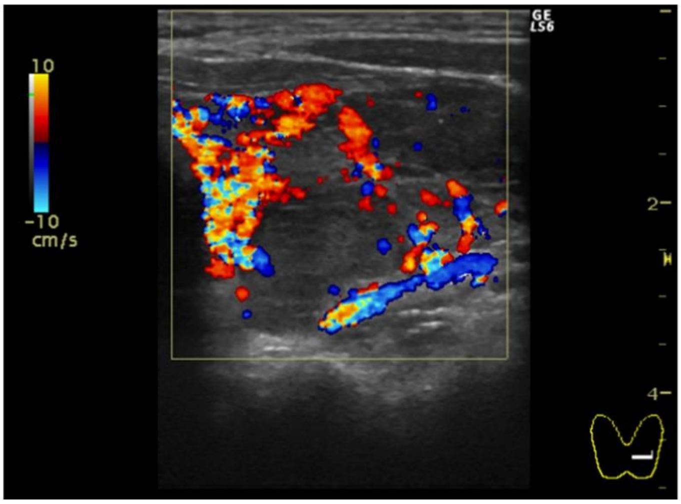

Family 32, the proband's grandmother

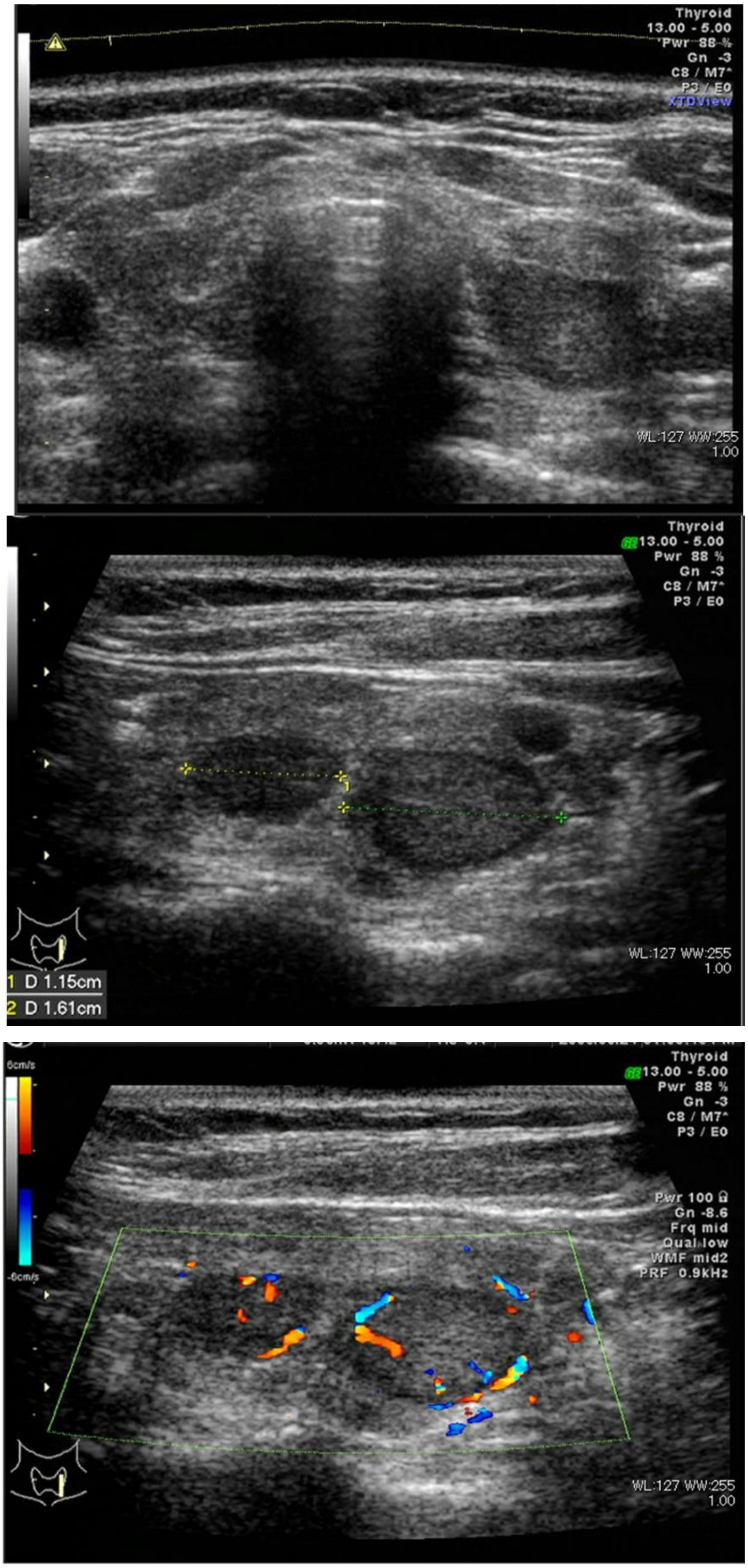

Family 47, the proband's mother

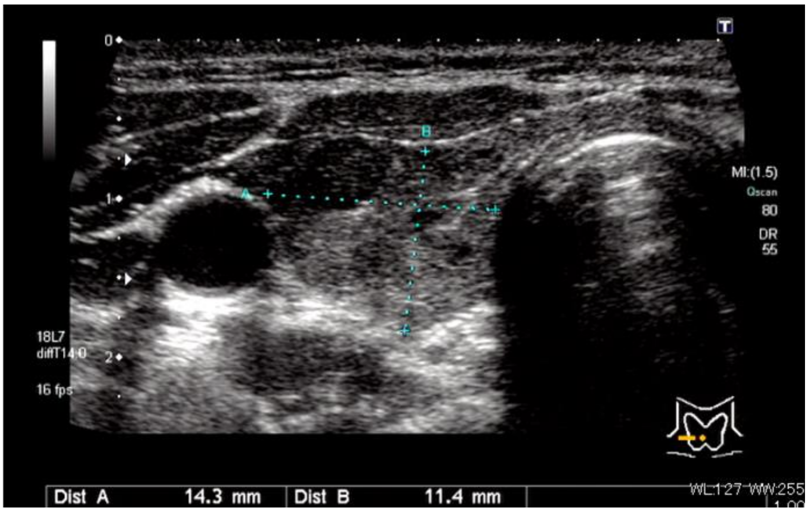

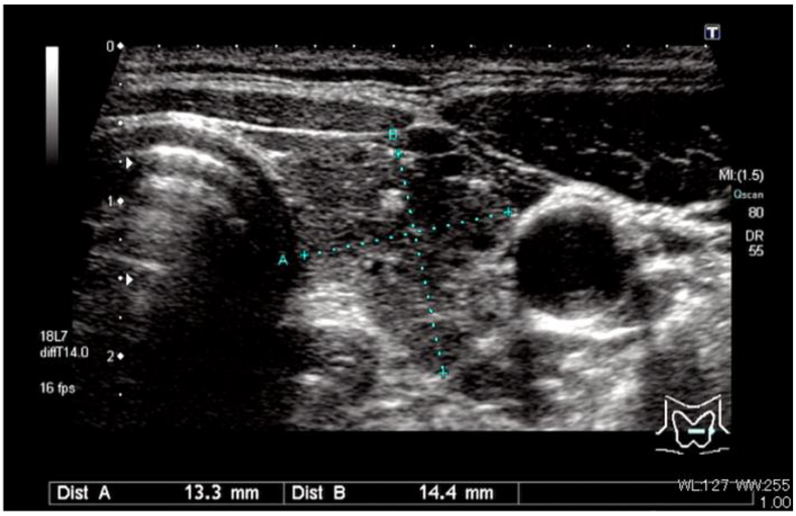

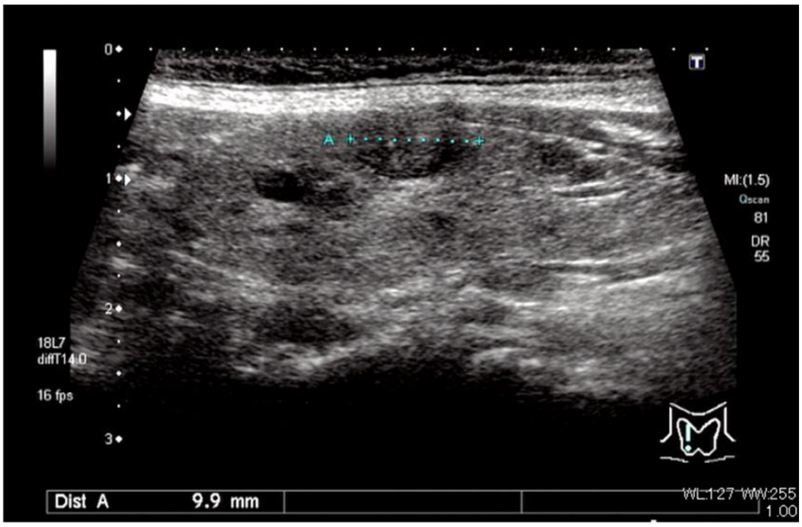

Family 47, the proband's uncle

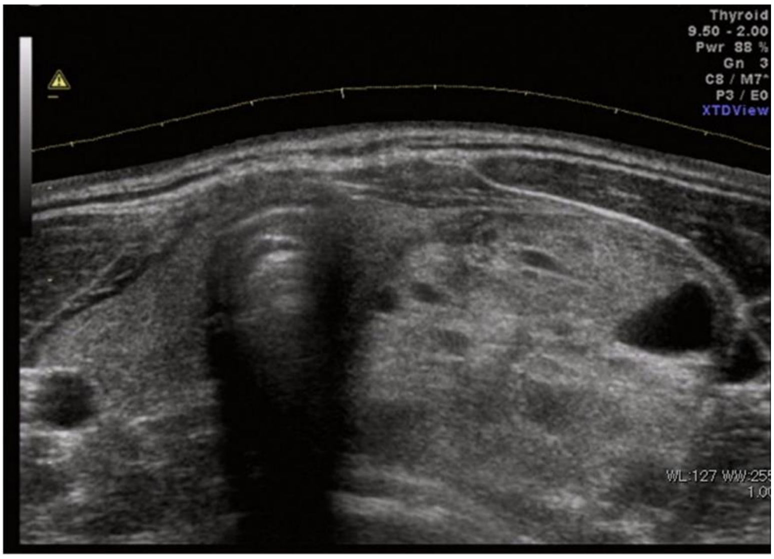

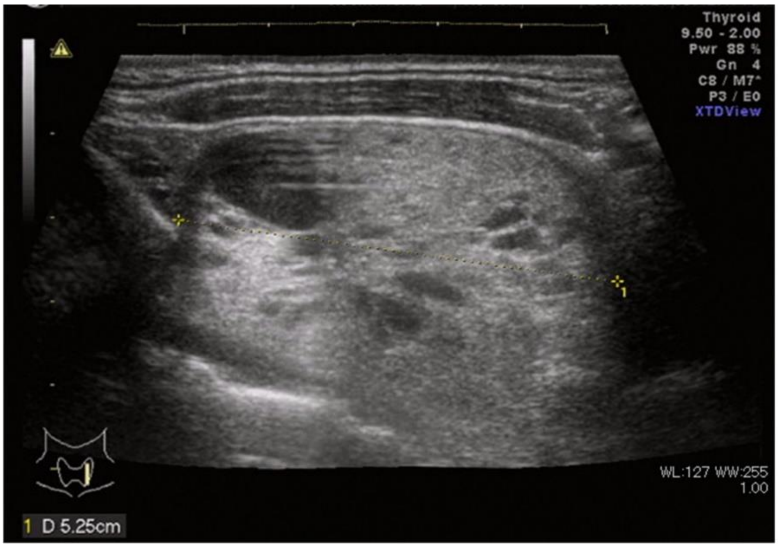

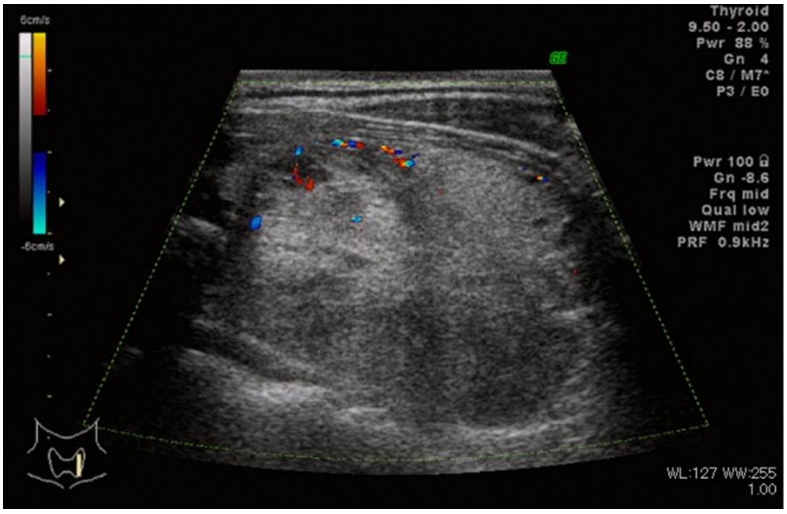

Family 59, the proband's mother

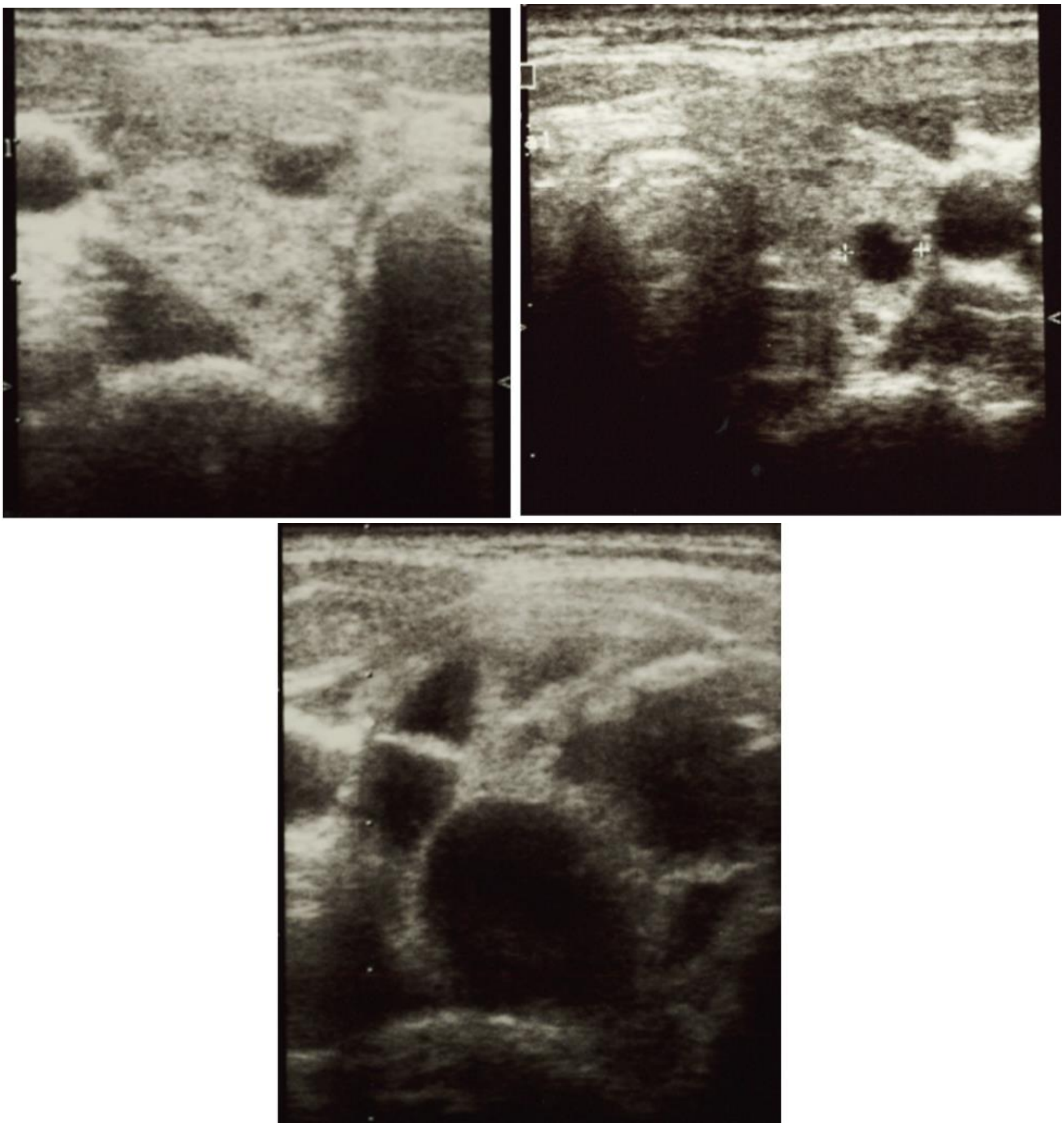

Family 60, the proband's father

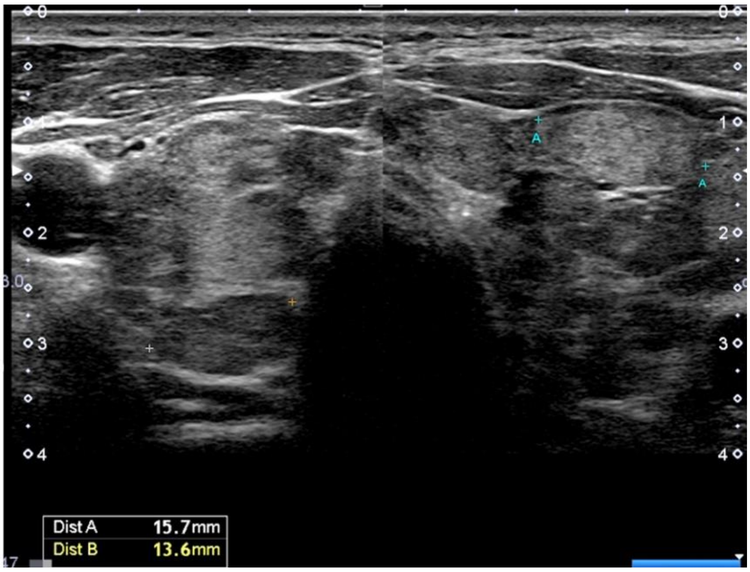

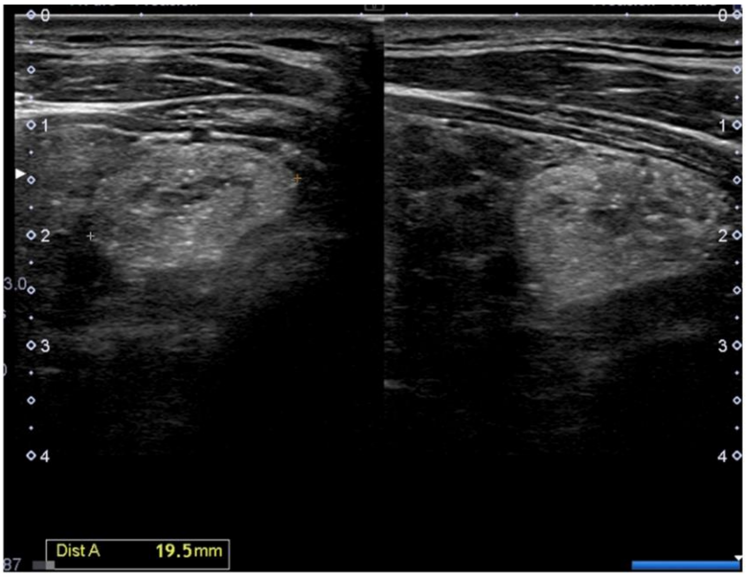

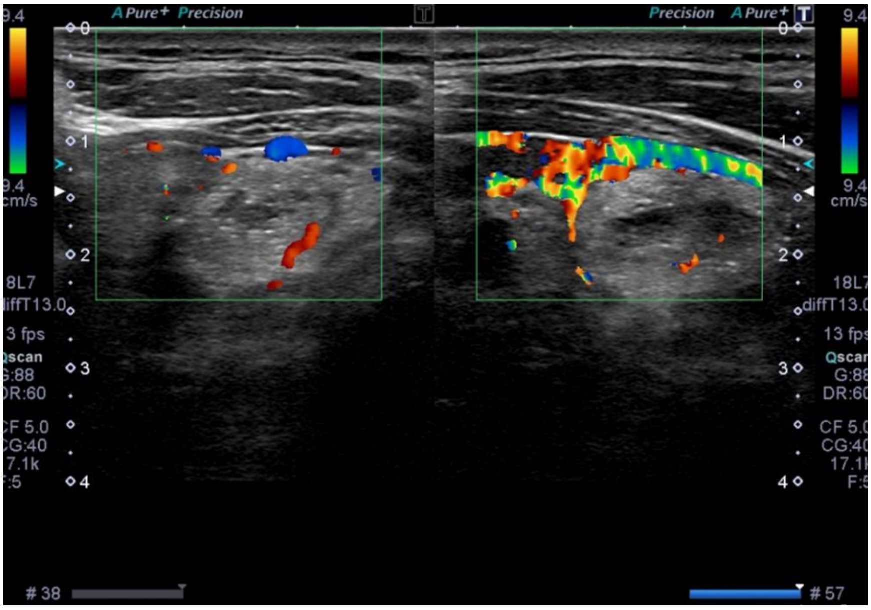

Family 66, the proband's father

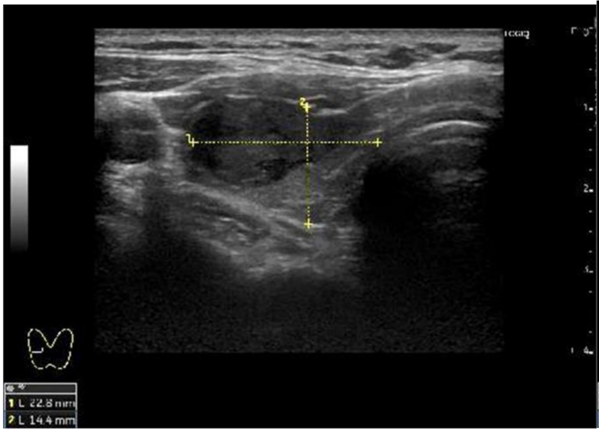

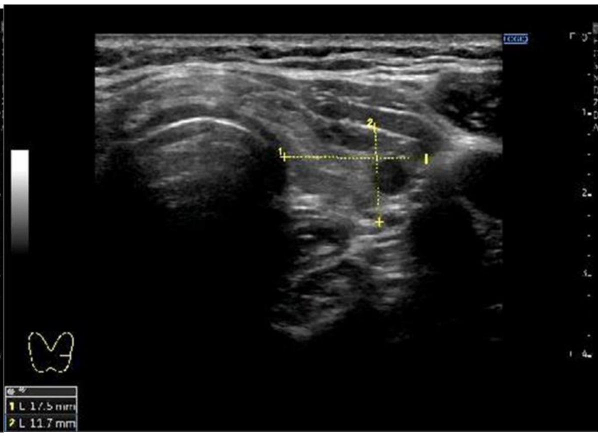

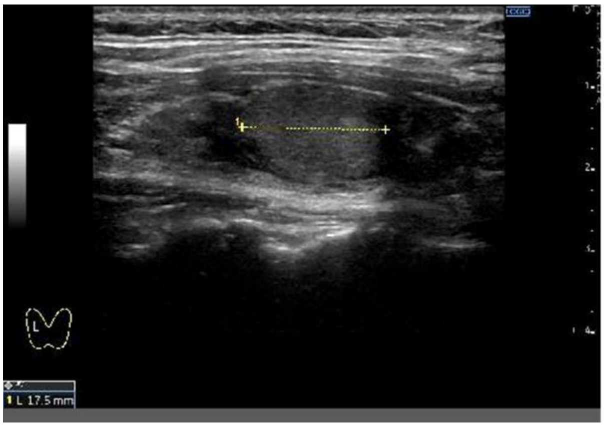

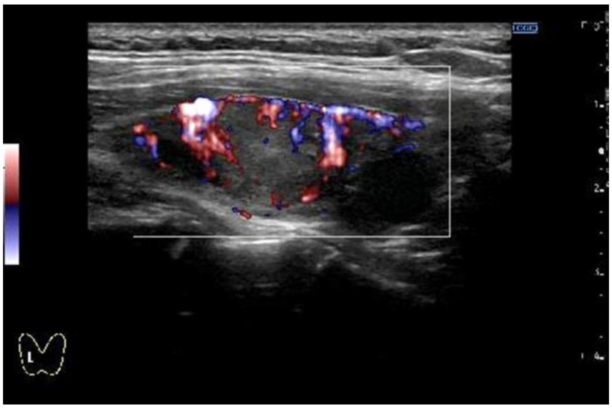

Family 72, the proband's grandmother

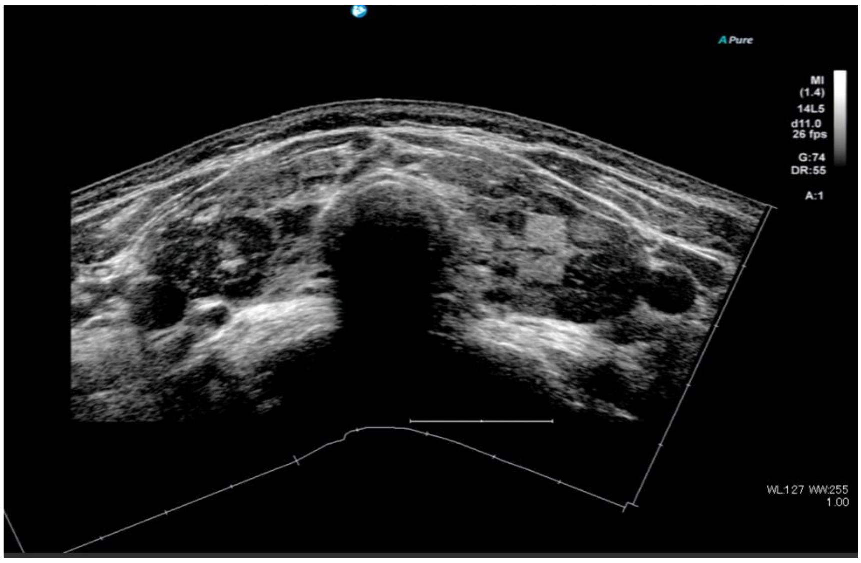

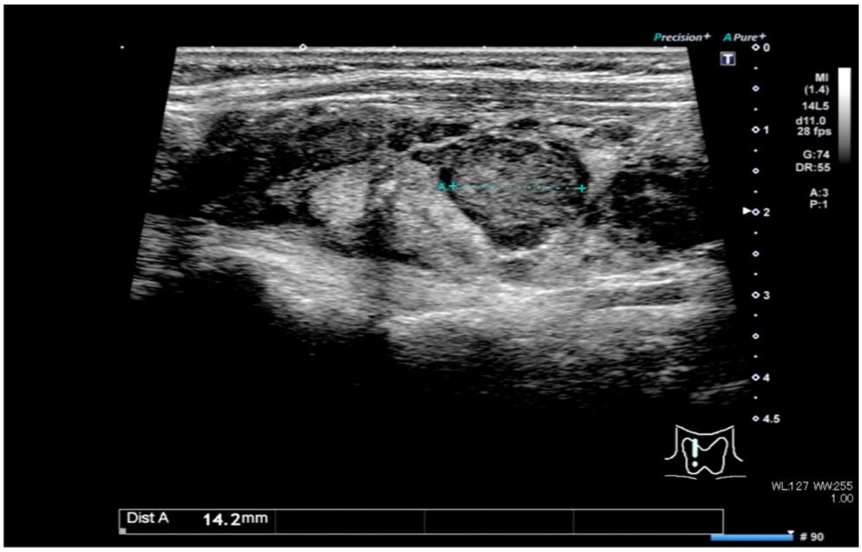

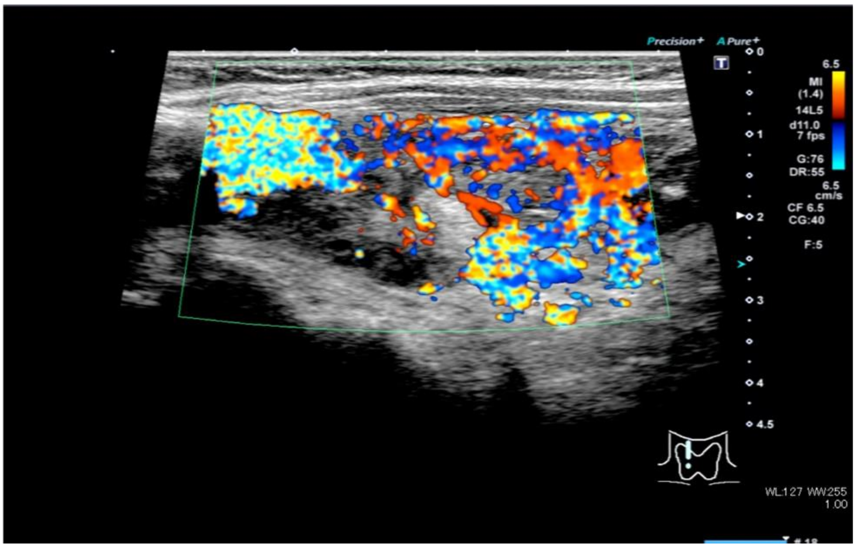

Family 76, the proband's mother

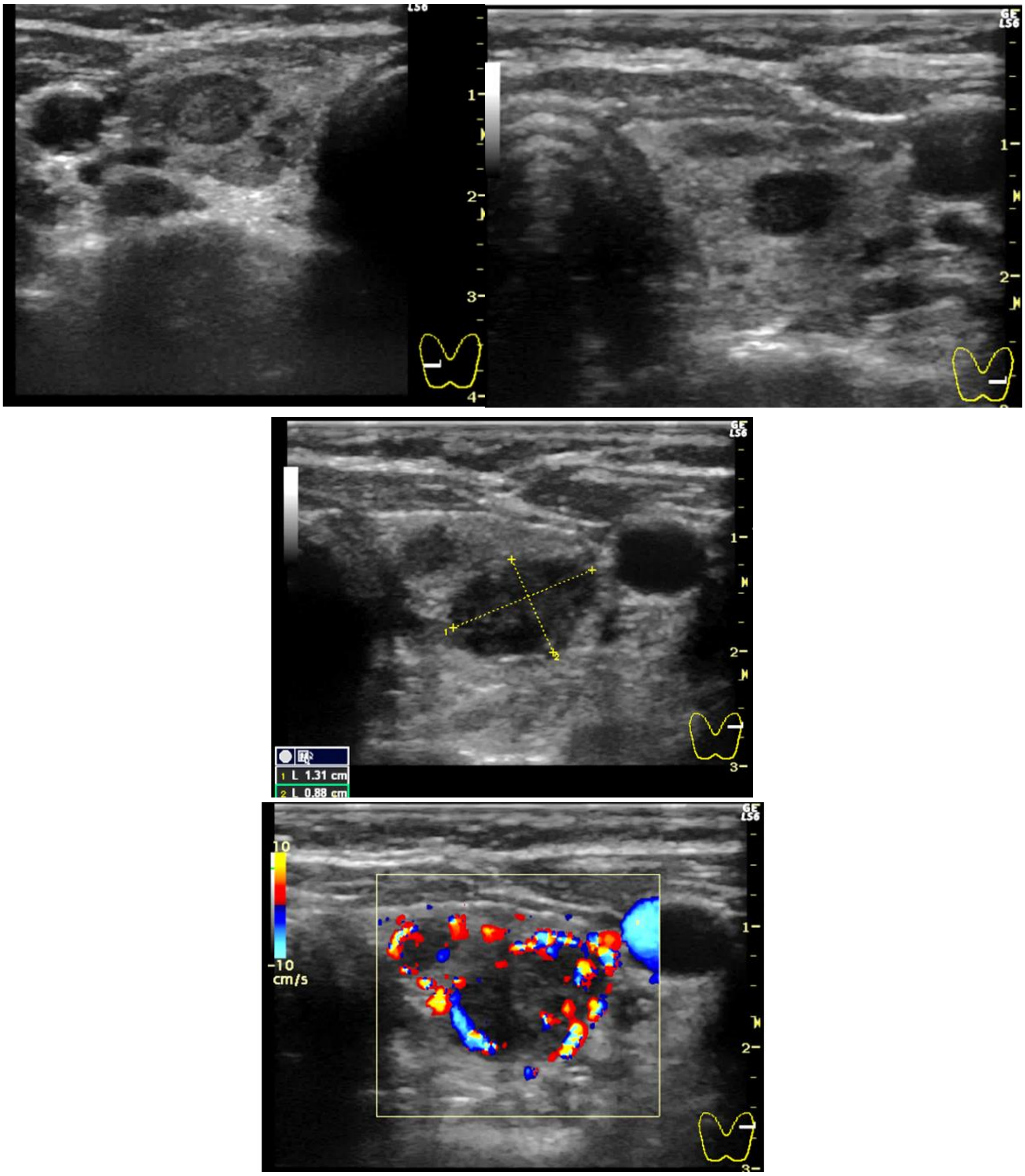

Family 86, the proband's mother

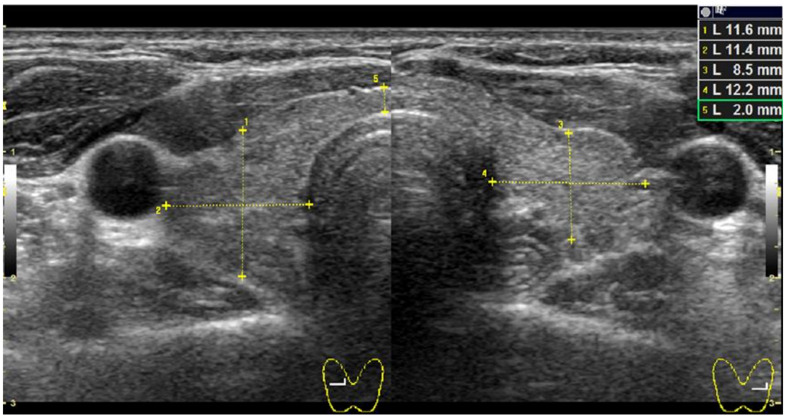

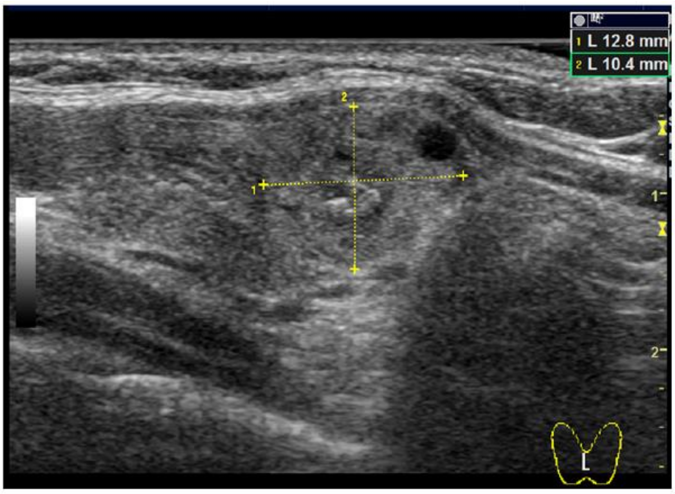

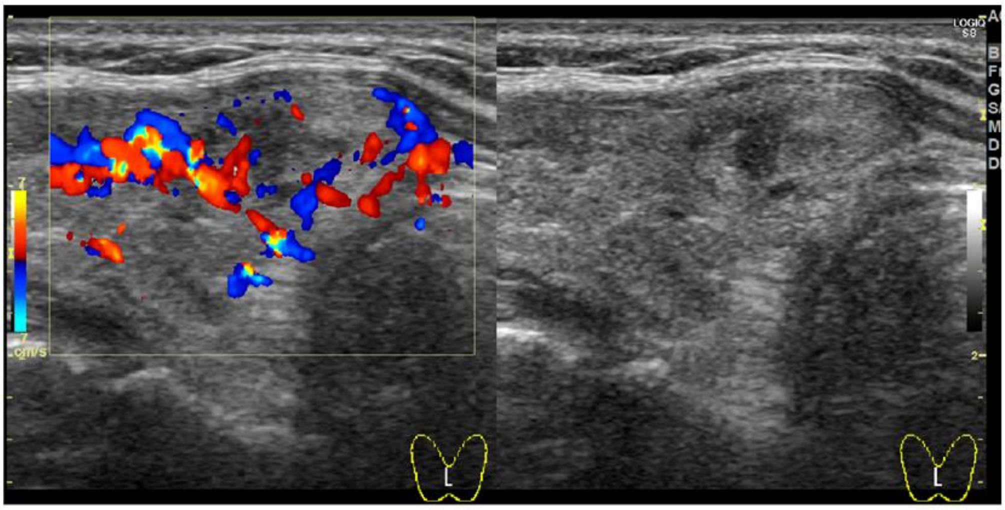

Family 81, the proband

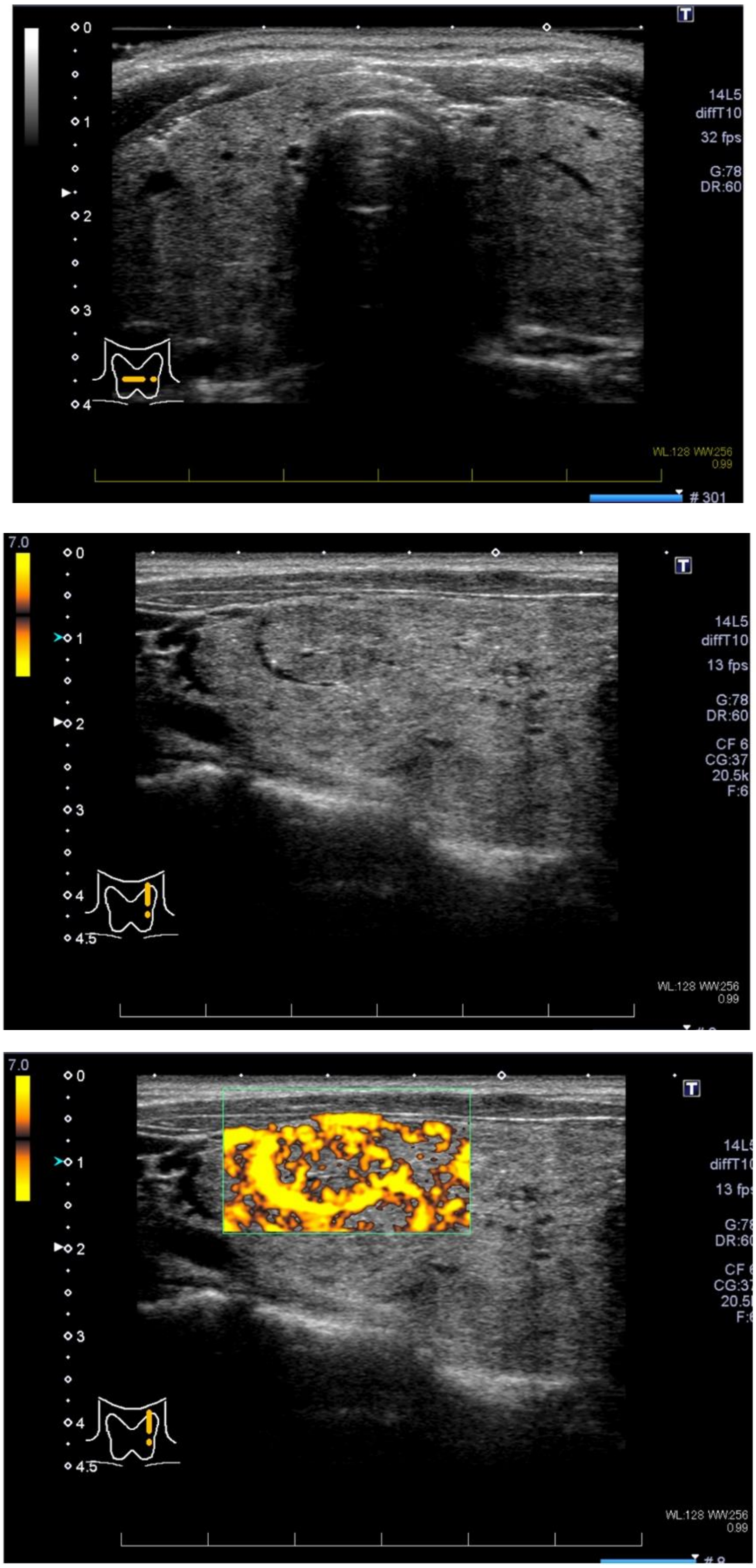

Family 83, the proband

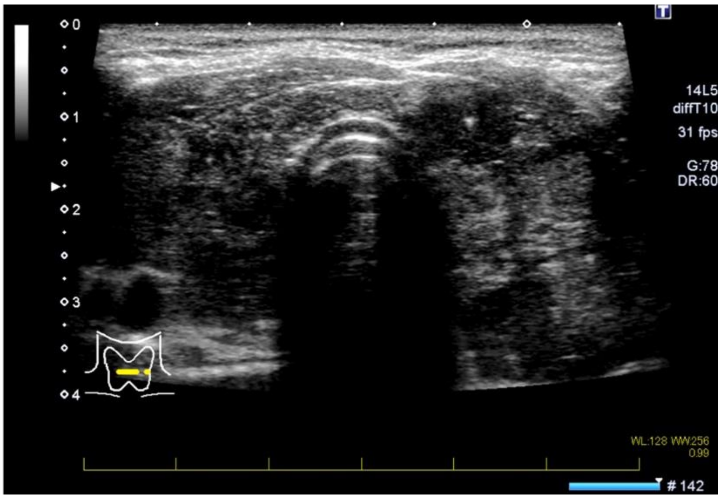

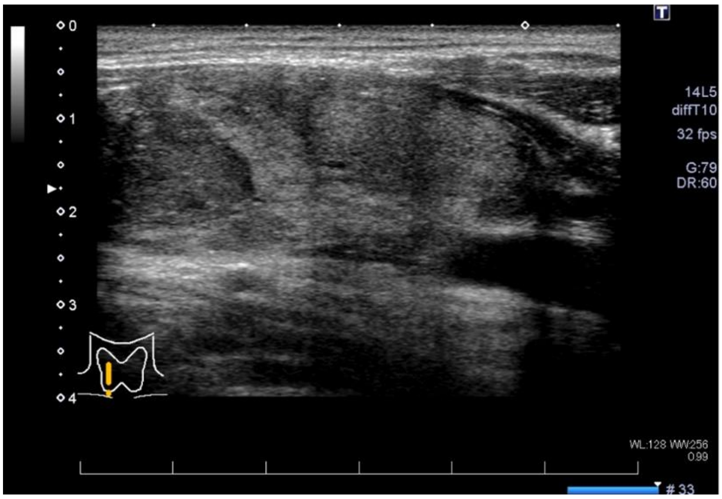

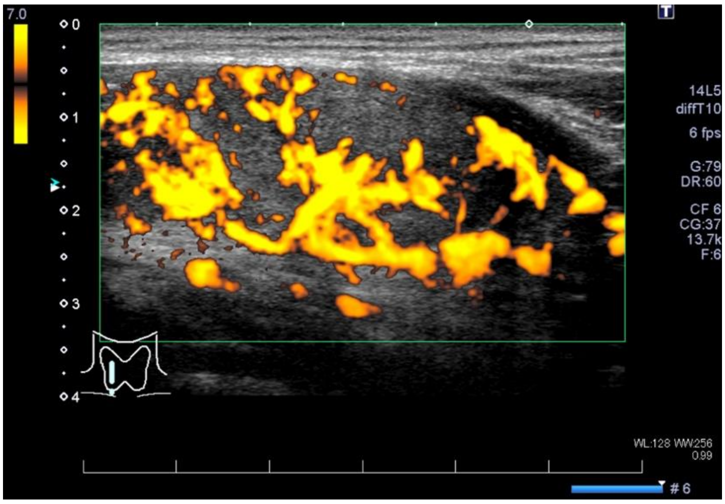

Family 84, the proband

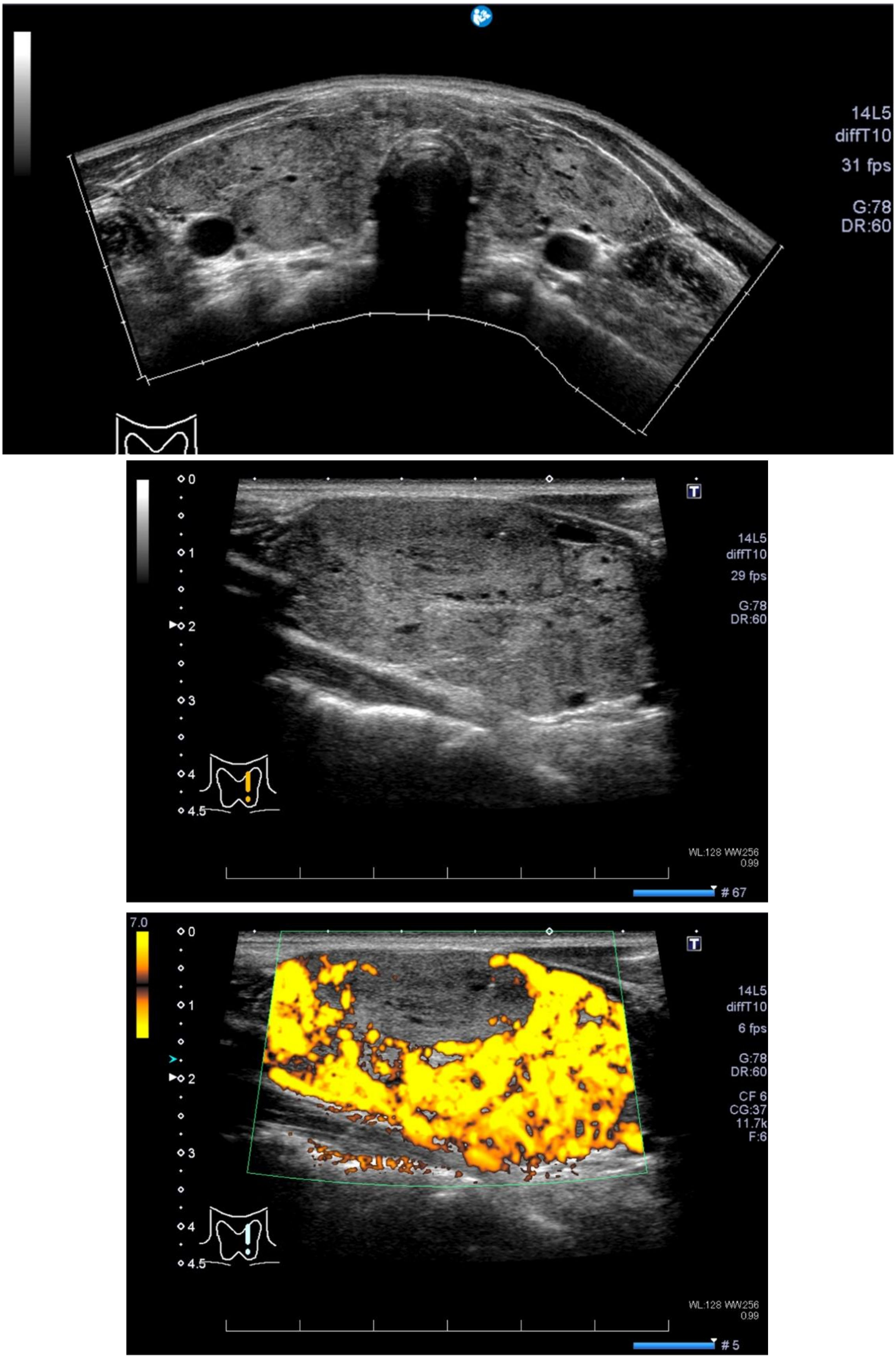

Supplement: Supplementary file 1 — Supplementary Methods and Figs. 1–4. [file 41588_2024_1735_MOESM1_ESM.pdf]
