# Supplementary material for: Functional variants in a TTTG microsatellite on 15q26.1 cause familial nonautoimmune thyroid abnormalities
Source: Nat Genet. 2024 May 7;56(5):869–76. doi: 10.1038/s41588-024-01735-5 (PMC11096107; doi:10.1038/s41588-024-01735-5)
Supplement: Supplementary file 2 — Reporting Summary [file 41588_2024_1735_MOESM2_ESM.pdf]

nature portfolio

Corresponding author(s): Satoshi Narumi

Last updated by author(s): Mar 15, 2024

Reporting Summary

Nature Portfolio wishes to improve the reproducibility of the work that we publish. This form provides structure for consistency and transparency in reporting. For further information on Nature Portfolio policies, see our Editorial Policies and the Editorial Policy Checklist.

Statistics

For all statistical analyses, confirm that the following items are present in the figure legend, table legend, main text, or Methods section.

n/a	Confirmed	
☐	☒	The exact sample size (*n*) for each experimental group/condition, given as a discrete number and unit of measurement
☐	☒	A statement on whether measurements were taken from distinct samples or whether the same sample was measured repeatedly
☐	☒	The statistical test(s) used AND whether they are one- or two-sided<br>*Only common tests should be described solely by name; describe more complex techniques in the Methods section.*
☒	☐	A description of all covariates tested
☐	☒	A description of any assumptions or corrections, such as tests of normality and adjustment for multiple comparisons
☐	☒	A full description of the statistical parameters including central tendency (e.g. means) or other basic estimates (e.g. regression coefficient) AND variation (e.g. standard deviation) or associated estimates of uncertainty (e.g. confidence intervals)
☐	☒	For null hypothesis testing, the test statistic (e.g. *F*, *t*, *r*) with confidence intervals, effect sizes, degrees of freedom and *P* value noted<br>*Give P values as exact values whenever suitable.*
☒	☐	For Bayesian analysis, information on the choice of priors and Markov chain Monte Carlo settings
☒	☐	For hierarchical and complex designs, identification of the appropriate level for tests and full reporting of outcomes
☒	☐	Estimates of effect sizes (e.g. Cohen's *d*, Pearson's *r*), indicating how they were calculated

Our web collection on statistics for biologists contains articles on many of the points above.

Software and code

Policy information about availability of computer code

Data collection	No software was used for data collection.
Data analysis	Superlink Online SNP version 1.1 (http://cbl-hap.cs.technion.ac.il/), Dragen v3.9.5, Juicebox Web App v2.3.5, Megadepth version 1.2.0, Microsoft Excel 2019

For manuscripts utilizing custom algorithms or software that are central to the research but not yet described in published literature, software must be made available to editors and reviewers. We strongly encourage code deposition in a community repository (e.g. GitHub). See the Nature Portfolio guidelines for submitting code & software for further information.

Data

Policy information about availability of data

All manuscripts must include a data availability statement. This statement should provide the following information, where applicable:

- Accession codes, unique identifiers, or web links for publicly available datasets
- A description of any restrictions on data availability
- For clinical datasets or third party data, please ensure that the statement adheres to our policy

Hi-C data of H1-hESC differentiated to definitive endoderm (accession number 4DNFIJWBWE41; 4D Nucleome Data Portal) were visualized using Juicebox Web App (https://aidenlab.org/juicebox/). snATAC-seq read count data were retrieved from Human Enhancer Atlas (http://catlas.org/humanenhancer/). Frequency data of genetic variants in 38,722 healthy Japanese individuals (38KJPN) were obtained from jMorp (https://jmorp.megabank.tohoku.ac.jp/). eQTL data (GTEx v8 dataset)

were downloaded from GTEx portal (https://gtexportal.org/home/datasets).
To preserve the confidentiality of the study participants, restrictions apply to the use of some of the data generated in this study. The corresponding author will, upon request, provide details of the restrictions and the conditions under which access to some of the data may be provided.

Research involving human participants, their data, or biological material

Policy information about studies with human participants or human data. See also policy information about sex, gender (identity/presentation), and sexual orientation and race, ethnicity and racism.

Reporting on sex and gender	Sex reported in this study is based on self-reported social sex. Except for thyroid ectopia, the contribution of sex to the onset and severity of congenital hypothyroidism has not been reported. To reduce complexity, this study does not present results stratified by sex.
Reporting on race, ethnicity, or other socially relevant groupings	This study was conducted in Japan, and all subjects were Japanese.
Population characteristics	The study subjects include patients with congenital hypothyroidism (female 53%; age <2 months). Adults with multinodular goiter (female 70%, median age 48 years, IQR 35 to 63 years) and relatives of the probands were also studied.
Recruitment	989 patients with congenital hypothyroidism were recruited at 119 institutions in Japan. The decision to recruit into the study was made by the primary physician. There was no participant compensation. The patient cohort may include patients with family history more preferentially.
Ethics oversight	This study was approved by ethics committees of Keio University School of Medicine (approval number, 20140289), National Center for Child Health and Development (approval number, 553), and Tohoku Medical Megabank Organization (approval number, 2022-4-186).

Note that full information on the approval of the study protocol must also be provided in the manuscript.

Field-specific reporting

Please select the one below that is the best fit for your research. If you are not sure, read the appropriate sections before making your selection.

☒ Life sciences ☐ Behavioural & social sciences ☐ Ecological, evolutionary & environmental sciences

For a reference copy of the document with all sections, see nature.com/documents/nr-reporting-summary-flat.pdf

Life sciences study design

All studies must disclose on these points even when the disclosure is negative.

Sample size	No statistical methods was used to determine sample size. We used one of the largest patient cohort of congenital hypothyroidism.
Data exclusions	No data were excluded from the study.
Replication	The luciferase assay was repeated independently for three times, each with hexaplicate, and all of those results were similar.
Randomization	This study is a cross-sectional observational study and therefore randomization was not conducted.
Blinding	Data collection and analysis were not performed blind to the conditions of the experiments.

Reporting for specific materials, systems and methods

We require information from authors about some types of materials, experimental systems and methods used in many studies. Here, indicate whether each material, system or method listed is relevant to your study. If you are not sure if a list item applies to your research, read the appropriate section before selecting a response.

Materials & experimental systems

n/a	Involved in the study	
☐	☒	Antibodies
☐	☒	Eukaryotic cell lines
☒	☐	Palaeontology and archaeology
☒	☐	Animals and other organisms
☐	☒	Clinical data
☒	☐	Dual use research of concern
☒	☐	Plants

Methods

n/a	Involved in the study	
☒	☐	ChIP-seq
☒	☐	Flow cytometry
☒	☐	MRI-based neuroimaging

Antibodies

Antibodies used: Rabbit anti-thyroglobulin (Tg) polyclonal antibody (A0251; Dako, Kyoto, Japan), diluted at 1:5,000 for immunohistochemistry.

Validation: The antibody labels human Tg, traces of contaminating antibodies have been removed by solid-phase absorption with human plasma proteins. In crossed immunoelectrophoresis using 12.5 µL antibody per cm2 gel area against 2 µL of human thyroid gland extract, only one precipitate corresponding to Tg appears. With 2 µL of human plasma, no precipitate appears. In human thyroid cell cultures, the antibody labels intracellular Tg (Kayser L et al., Histochem J. 1991;23:235-240).

Eukaryotic cell lines

Policy information about cell lines and Sex and Gender in Research

Cell line source(s): FRTL-5 (Interthyr Research Foundation) and HEK293 (ATCC)

Authentication: HEK293 cells were purchased from ATCC, and only early passage cells (<10) were used. As for FRTL-5 cells, we have confirmed that the thyroid-specific molecules (e.g., TSH receptor and thyroglobulin) are expressed at high levels, and retain the natures as differentiated thyroid follicular cells (Kiriya M. et al., Endocr J. 2022;69:1217-1225).

Mycoplasma contamination: Cells were not tested for mycoplasma contamination.

Commonly misidentified lines (See ICLAC register): No commonly misidentified cell lines were used.

Clinical data

Policy information about clinical studies

All manuscripts should comply with the ICMJE guidelines for publication of clinical research and a completed CONSORT checklist must be included with all submissions.

Clinical trial registration: This study is not a clinical trial.

Study protocol: This study is not a clinical trial. The protocol for genetic studies will be provided by the corresponding author upon request.

Data collection: From 2006 to 2022, clinical information and genetic samples were collected at 119 medical institutions in Japan.

Outcomes: Because this study is not a clinical trial, there are no pre-defined outcomes.
