# Supplementary material for: Functional variants in a TTTG microsatellite on 15q26.1 cause familial nonautoimmune thyroid abnormalities
Source: Nat Genet. 2024 May 7;56(5):869–76. doi: 10.1038/s41588-024-01735-5 (PMC11096107; doi:10.1038/s41588-024-01735-5)

Source data Fig. 3b

Ultrasonography

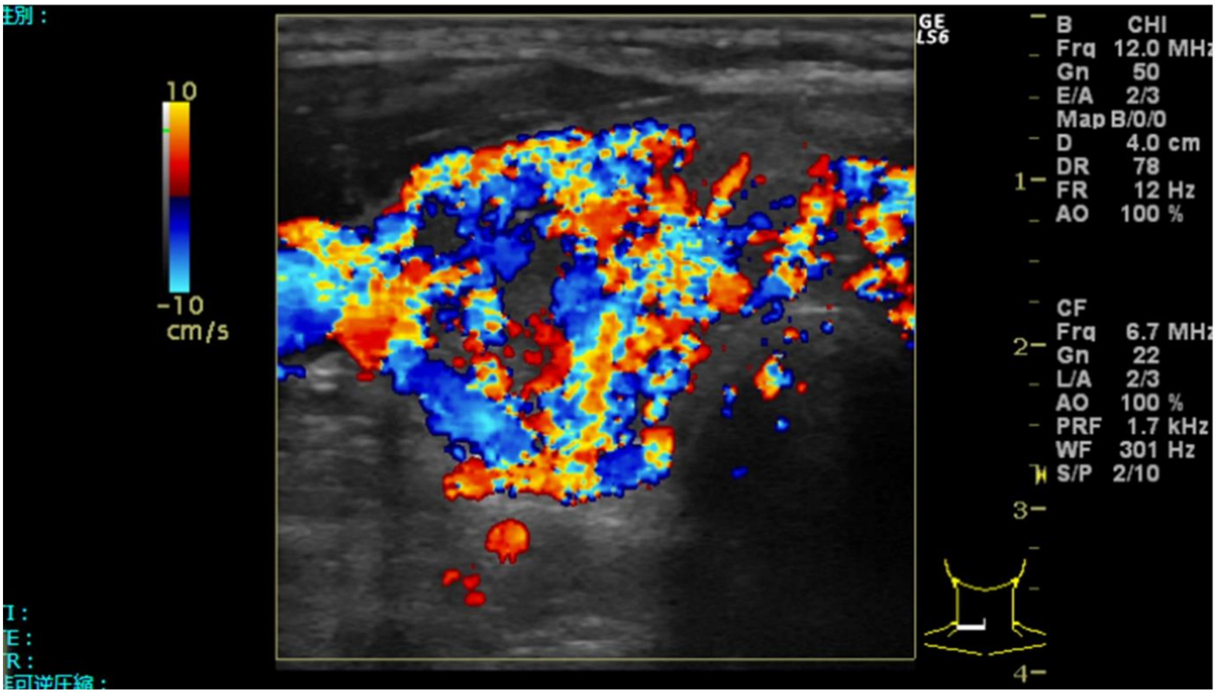

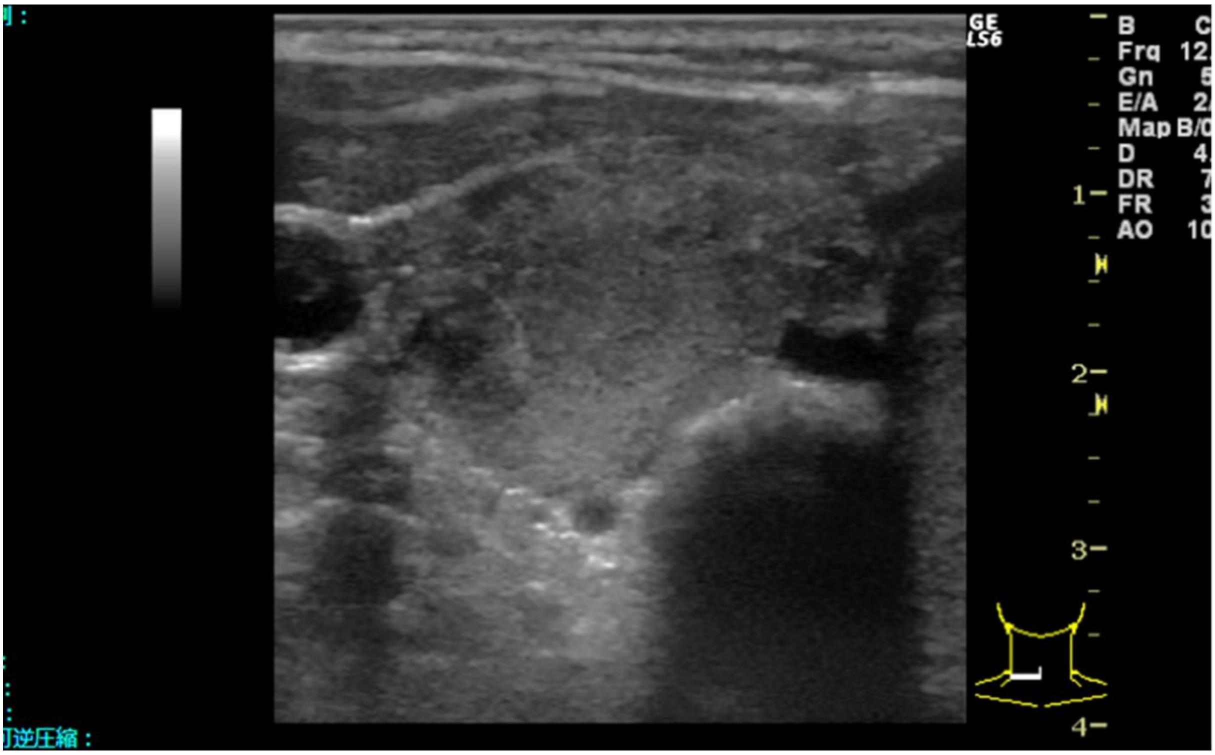

Fig. 3c

Histology

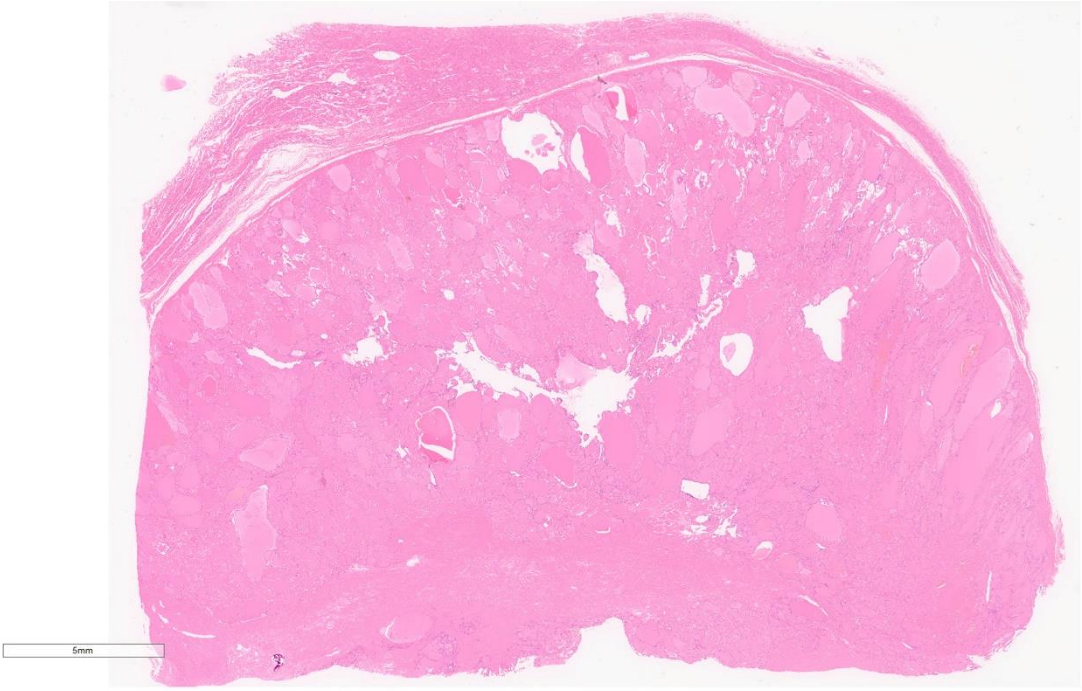

Normal tissue

H&E

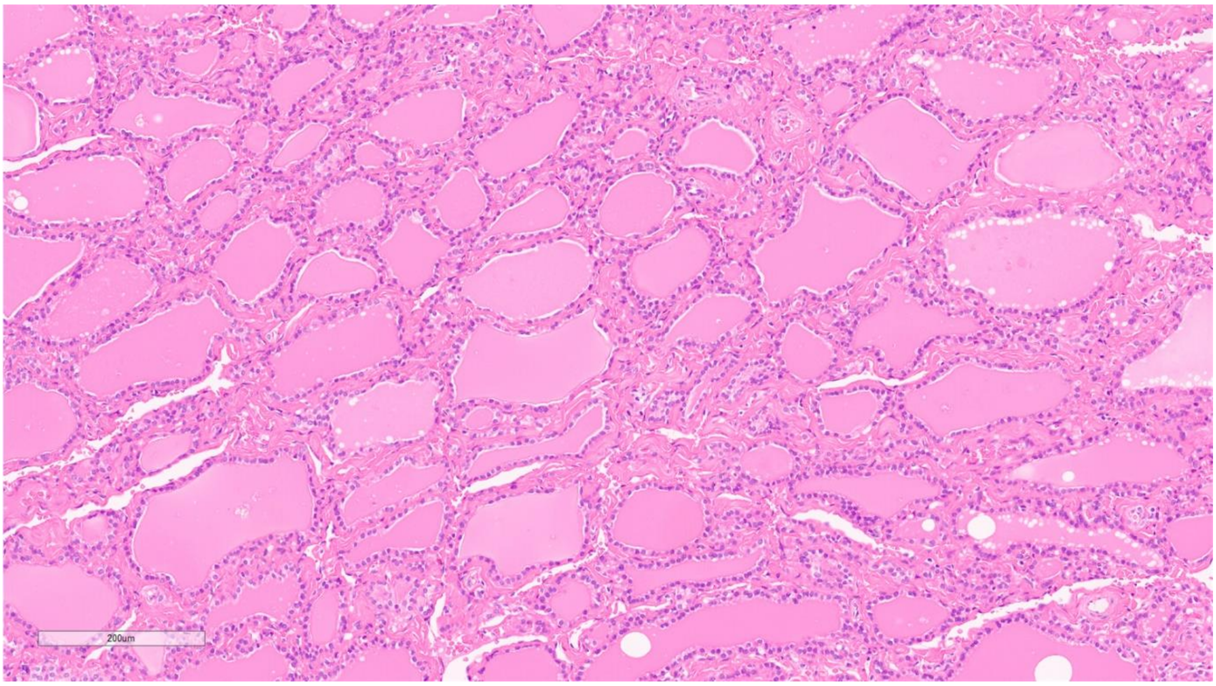

Thyroglobulin staining

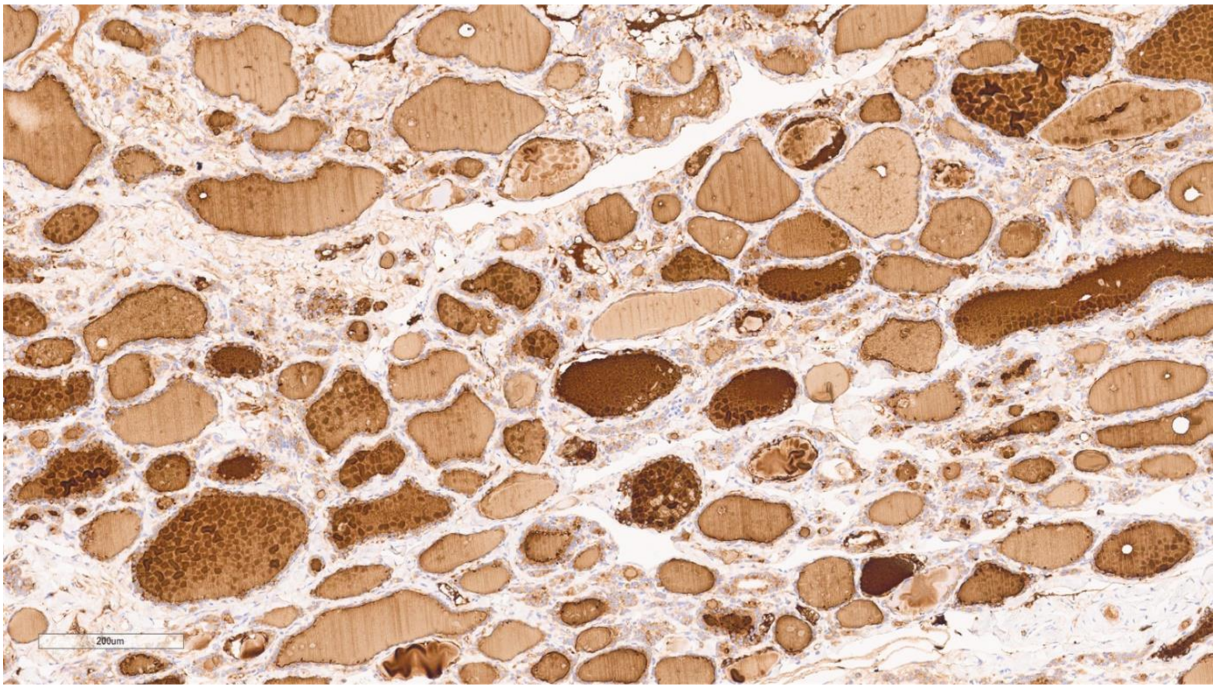

Nodule

H&E

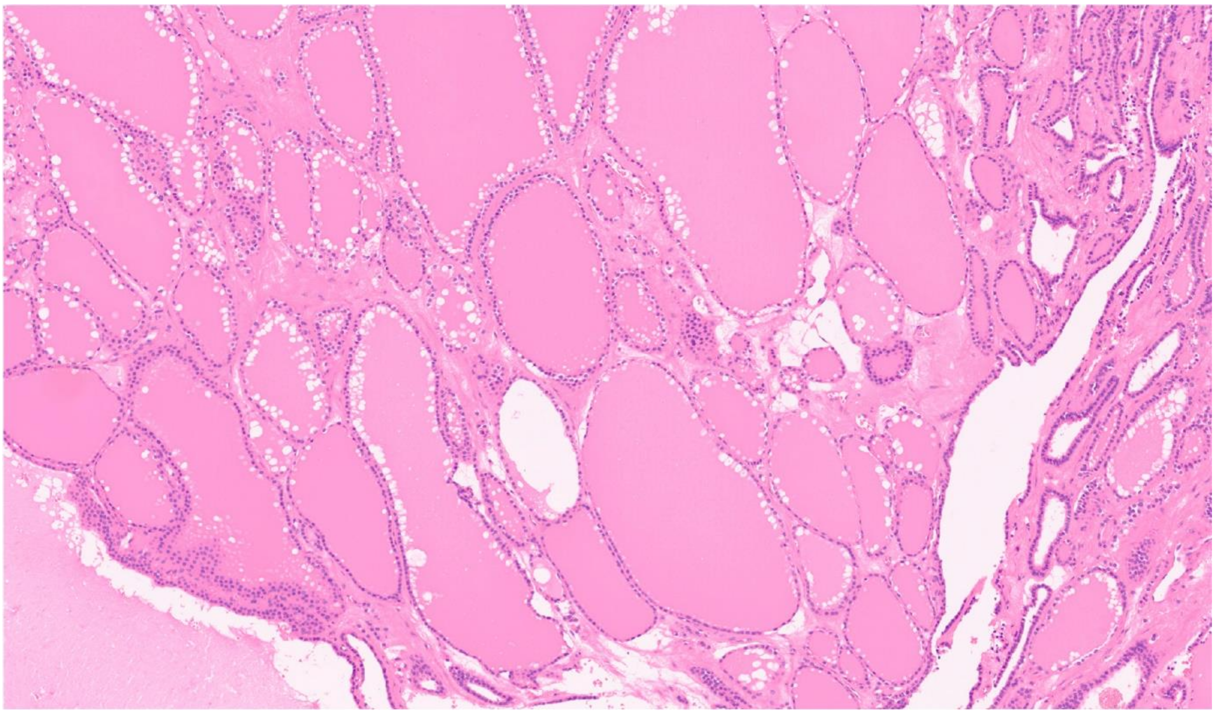

Thyroglobulin staining

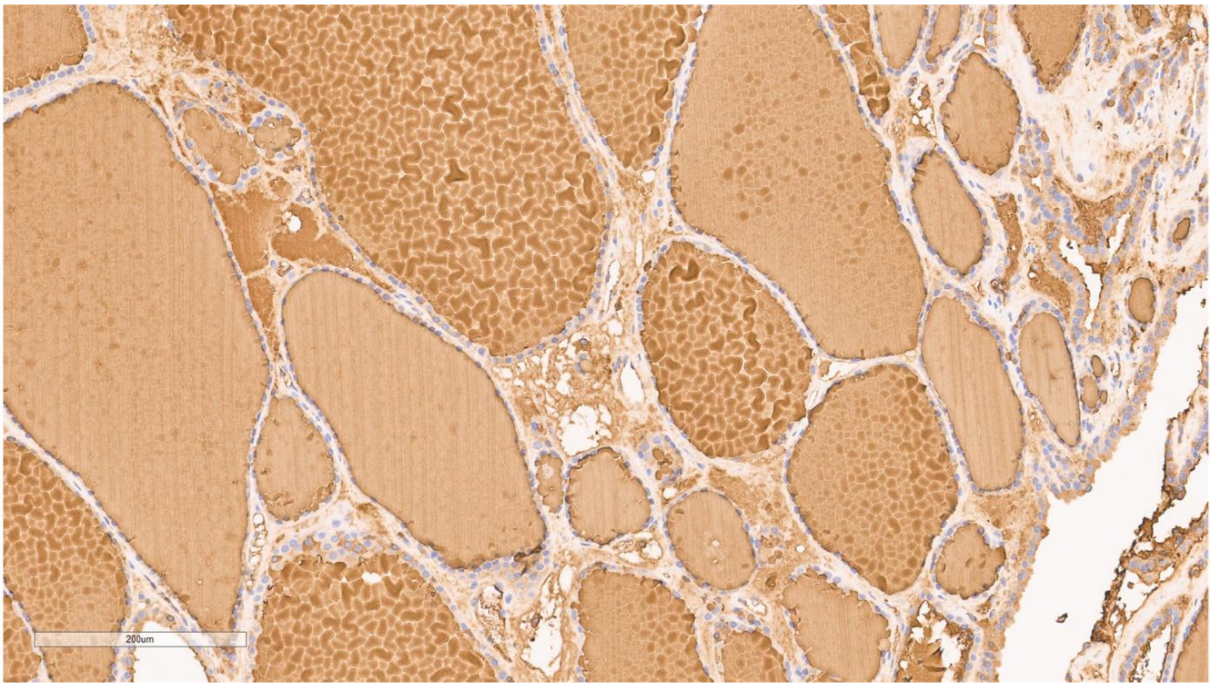

Supplement: Supplementary file 7 — Unprocessed images of ultrasonography and histology. [file 41588_2024_1735_MOESM7_ESM.pdf]
